# Supplementary material for: Spatiotemporal Patterns of Non-Communicable Disease Mortality in the Metropolitan Area of the Valley of Mexico, 2000–2019
Source: Diseases. 2025 Aug 1;13(8):241. doi: 10.3390/diseases13080241 (PMC12385793; doi:10.3390/diseases13080241)
Supplement: Supplementary file 1 [file diseases-13-00241-s001.zip › diseases-3741061 - Supplementary material.pdf]

Spatiotemporal patterns of non-communicable disease mortality in the Metropolitan Area of the Valley of Mexico, 2000-2019

Constantino González-Salazar, Kathia Gasca-Gómez and Omar Cordero-Saldierna

Supplementary material

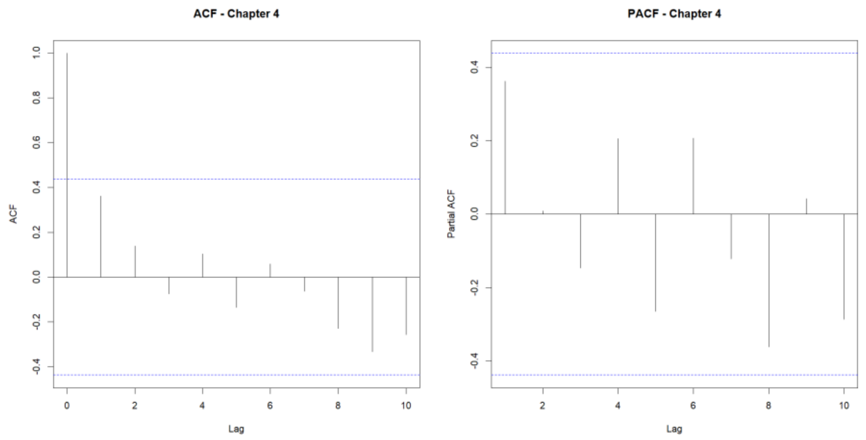

Figure S1. Autocorrelation (ACF) and partial autocorrelation (PACF) plots of residuals for linear trend models of ICD-10 chapter 4. Note: The dashed blue lines represent 95% confidence bounds.

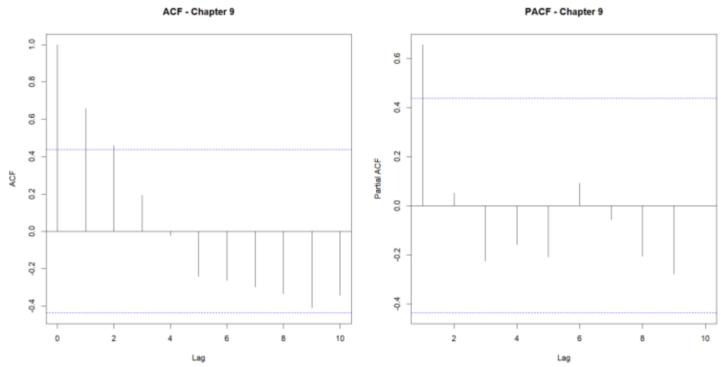

Figure S2. Autocorrelation (ACF) and partial autocorrelation (PACF) plots of residuals for linear trend models of ICD-10 chapter 9. Note: The dashed blue lines represent 95% confidence bounds.

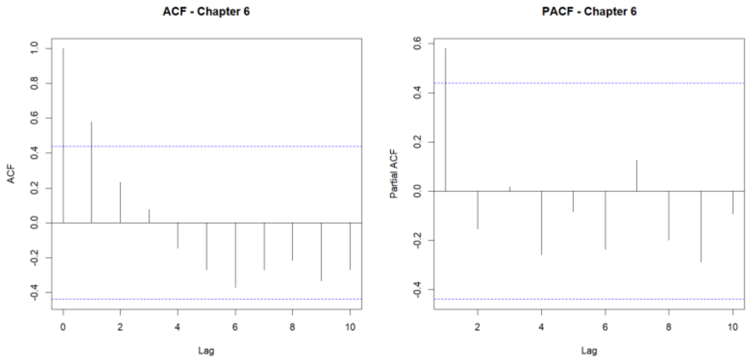

Figure S3. Autocorrelation (ACF) and partial autocorrelation (PACF) plots of residuals for linear trend models of ICD-10 chapter 6. Note: The dashed blue lines represent 95% confidence bounds.

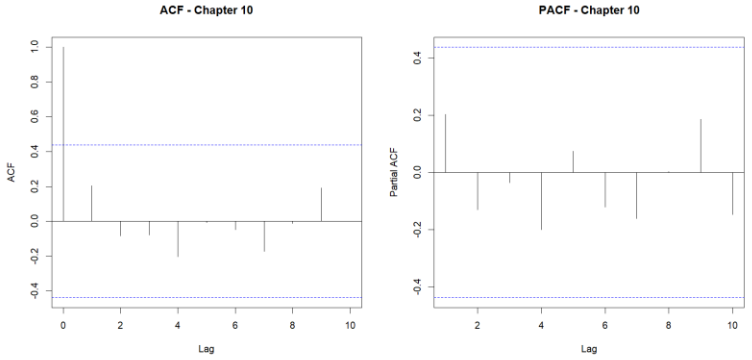

Figure S4. Autocorrelation (ACF) and partial autocorrelation (PACF) plots of residuals for linear trend models of ICD-10 chapter 10. Note: The dashed blue lines represent 95% confidence bounds.

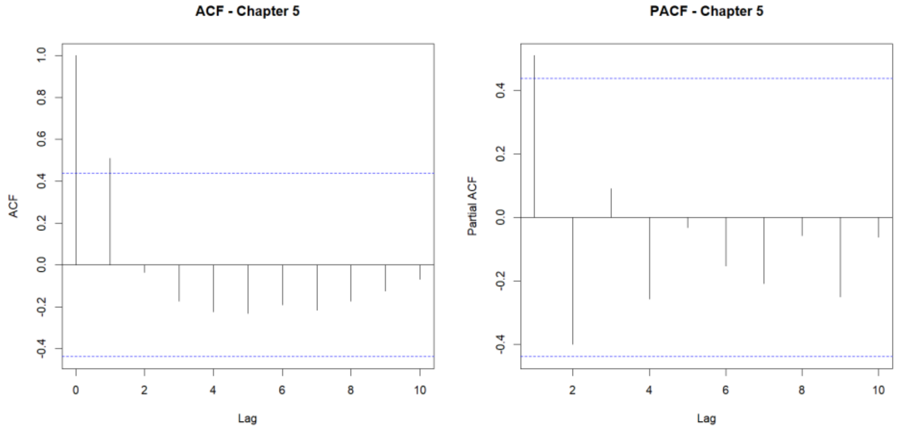

Figure S5. Autocorrelation (ACF) and partial autocorrelation (PACF) plots of residuals for linear trend models of ICD-10 chapter 5. Note: The dashed blue lines represent 95% confidence bounds.

Table S2. Ljung–Box test results for residual autocorrelation by ICD-10 chapter and sex at the MAVM level. (*) indicates statistically significant autocorrelation.

**ICD-10 Chapter**	**Sex**	**Ljung–Box *Q****	***df***	***p-value***
Chapter 4 (Endocrine, nutritional and metabolic diseases)	Men	15.3	10	0.12087
Chapter 4 (Endocrine, nutritional and metabolic diseases)	Women	12.1	10	0.27571
Chapter 5 (Mental and behavioural disorders)	Men	20.7	10	**0.02342***
Chapter 5 (Mental and behavioural disorders)	Women	17.9	10	0.05750
Chapter 6 (Diseases of the nervous system)	Men	14.8	10	0.13812
Chapter 6 (Diseases of the nervous system)	Women	29.9	10	**0.00088***
Chapter 9 (Diseases of the circulatory system)	Men	30.1	10	**0.00083***
Chapter 9 (Diseases of the circulatory system)	Women	42.1	10	**0.00001***
Chapter 10 (Diseases of the respiratory system)	Men	4.1	10	0.94153
Chapter 10 (Diseases of the respiratory system)	Women	6.1	10	0.80436

Table S3. Ljung–Box test results for residual autocorrelation by ICD-10 chapter and age group at the MAVM level. (*) indicates statistically significant autocorrelation.

**ICD-10 Chapter**	**Age gpo**	**Ljung–Box *Q****	***df***	***p-value***
Chapter 4 (Endocrine, nutritional and metabolic diseases)	0-4	8.1	10	0.62038
Chapter 4 (Endocrine, nutritional and metabolic diseases)	5-14	6.4	10	0.77954
Chapter 4 (Endocrine, nutritional and metabolic diseases)	15-24	8.3	10	0.59749
Chapter 4 (Endocrine, nutritional and metabolic diseases)	25-34	37.7	10	**0.00004***
Chapter 4 (Endocrine, nutritional and metabolic diseases)	35-44	24.6	10	**0.00622***
Chapter 4 (Endocrine, nutritional and metabolic diseases)	45-54	13.8	10	0.18341
Chapter 4 (Endocrine, nutritional and metabolic diseases)	55-64	32.0	10	**0.00041***
Chapter 4 (Endocrine, nutritional and metabolic diseases)	65+	38.8	10	**0.00003***
Chapter 5 (Mental and behavioural disorders)	15-24	10.7	10	0.37940
Chapter 5 (Mental and behavioural disorders)	25-34	18.6	10	**0.04520***
Chapter 5 (Mental and behavioural disorders)	35-44	21.2	10	**0.01986***
Chapter 5 (Mental and behavioural disorders)	45-54	14.0	10	0.17089
Chapter 5 (Mental and behavioural disorders)	55-64	26.5	10	**0.00308***
Chapter 5 (Mental and behavioural disorders)	65+	8.5	10	0.58146
Chapter 5 (Mental and behavioural disorders)	5-14	9.0	10	0.53520
Chapter 5 (Mental and behavioural disorders)	0-4	17.8	10	0.05795
Chapter 6 (Diseases of the nervous system)	0-4	10.2	10	0.42264
Chapter 6 (Diseases of the nervous system)	5-14	9.2	10	0.51510
Chapter 6 (Diseases of the nervous system)	15-24	7.1	10	0.71913
Chapter 6 (Diseases of the nervous system)	25-34	5.3	10	0.86881
Chapter 6 (Diseases of the nervous system)	35-44	7.4	10	0.68758
Chapter 6 (Diseases of the nervous system)	45-54	13.6	10	0.19050
Chapter 6 (Diseases of the nervous system)	55-64	10.9	10	0.36221
Chapter 6 (Diseases of the nervous system)	65+	23.4	10	**0.00940***
Chapter 9 (Diseases of the circulatory system)	0-4	26.3	10	**0.00333***
Chapter 9 (Diseases of the circulatory system)	5-14	11.8	10	0.29629

Chapter 9 (Diseases of the circulatory system)	15-24	24.7	10	**0.00599***
Chapter 9 (Diseases of the circulatory system)	25-34	6.0	10	0.81650
Chapter 9 (Diseases of the circulatory system)	35-44	14.1	10	0.16808
Chapter 9 (Diseases of the circulatory system)	45-54	7.6	10	0.66813
Chapter 9 (Diseases of the circulatory system)	55-64	7.5	10	0.67937
Chapter 9 (Diseases of the circulatory system)	65+	8.5	10	0.57788
Chapter 10 (Diseases of the respiratory system)	0-4	22.1	10	**0.01442***
Chapter 10 (Diseases of the respiratory system)	5-14	21.1	10	**0.02045***
Chapter 10 (Diseases of the respiratory system)	15-24	19.5	10	**0.03427***
Chapter 10 (Diseases of the respiratory system)	25-34	5.9	10	0.82665
Chapter 10 (Diseases of the respiratory system)	35-44	5.0	10	0.89103
Chapter 10 (Diseases of the respiratory system)	45-54	12.9	10	0.23020
Chapter 10 (Diseases of the respiratory system)	55-64	8.3	10	0.60097
Chapter 10 (Diseases of the respiratory system)	65+	9.9	10	0.44828

Table S4. Ljung–Box test results for residual autocorrelation by ICD-10 chapter at the municipality level. (*) indicates statistically significant autocorrelation.

**State**	**Municipality**	**ICD-10 Chapter**	**Ljung–Box Q***	**df**	**p-value**
Mexico City	Azcapotzalco	Chapter 9 (Diseases of the circulatory system)	8.3	10	0.59983
Mexico City	Azcapotzalco	Chapter 4 (Endocrine, nutritional and metabolic diseases)	9.5	10	0.48527
Mexico City	Azcapotzalco	Chapter 6 (Diseases of the nervous system)	16.2	10	0.09304
Mexico City	Azcapotzalco	Chapter 10 (Diseases of the respiratory system)	6.3	10	0.78767
Mexico City	Azcapotzalco	Chapter 5 (Mental and behavioural disorders)	8.1	10	0.61538
Mexico City	Coyoacán	Chapter 9 (Diseases of the circulatory system)	16.6	10	0.08473
Mexico City	Coyoacán	Chapter 4 (Endocrine, nutritional and metabolic diseases)	21.6	10	**0.01745***
Mexico City	Coyoacán	Chapter 6 (Diseases of the nervous system)	3.3	10	0.97230
Mexico City	Coyoacán	Chapter 10 (Diseases of the respiratory system)	6.6	10	0.76276
Mexico City	Coyoacán	Chapter 5 (Mental and behavioural disorders)	12.8	10	0.23752
Mexico City	Cuajimalpa de Morelos	Chapter 9 (Diseases of the circulatory system)	39.7	10	**0.00002***
Mexico City	Cuajimalpa de Morelos	Chapter 4 (Endocrine, nutritional and metabolic diseases)	34.9	10	**0.00013***
Mexico City	Cuajimalpa de Morelos	Chapter 6 (Diseases of the nervous system)	5.6	10	0.84713
Mexico City	Cuajimalpa de Morelos	Chapter 10 (Diseases of the respiratory system)	10.9	10	0.36525
Mexico City	Cuajimalpa de Morelos	Chapter 5 (Mental and behavioural disorders)	15.5	10	0.11334
Mexico City	Gustavo A. Madero	Chapter 9 (Diseases of the circulatory system)	25.7	10	**0.00421***
Mexico City	Gustavo A. Madero	Chapter 4 (Endocrine, nutritional and metabolic diseases)	16.3	10	0.09200
Mexico City	Gustavo A. Madero	Chapter 6 (Diseases of the nervous system)	19.1	10	**0.03959***
Mexico City	Gustavo A. Madero	Chapter 10 (Diseases of the respiratory system)	9.8	10	0.46162
Mexico City	Gustavo A. Madero	Chapter 5 (Mental and behavioural disorders)	15.5	10	0.11374
Mexico City	Iztacalco	Chapter 9 (Diseases of the circulatory system)	7.1	10	0.71262
Mexico City	Iztacalco	Chapter 4 (Endocrine, nutritional and metabolic diseases)	16.4	10	0.08767
Mexico City	Iztacalco	Chapter 6 (Diseases of the nervous system)	11.3	10	0.33632
Mexico City	Iztacalco	Chapter 10 (Diseases of the respiratory system)	4.0	10	0.94788
Mexico City	Iztacalco	Chapter 5 (Mental and behavioural disorders)	15.9	10	0.10286

Mexico City	Iztapalapa	Chapter 9 (Diseases of the circulatory system)	12.5	10	0.25222
Mexico City	Iztapalapa	Chapter 4 (Endocrine, nutritional and metabolic diseases)	6.6	10	0.76369
Mexico City	Iztapalapa	Chapter 6 (Diseases of the nervous system)	14.0	10	0.17334
Mexico City	Iztapalapa	Chapter 10 (Diseases of the respiratory system)	28.7	10	**0.00140***
Mexico City	Iztapalapa	Chapter 5 (Mental and behavioural disorders)	11.5	10	0.31922
Mexico City	La Magdalena Contreras	Chapter 9 (Diseases of the circulatory system)	28.0	10	**0.00182***
Mexico City	La Magdalena Contreras	Chapter 4 (Endocrine, nutritional and metabolic diseases)	16.9	10	0.07718
Mexico City	La Magdalena Contreras	Chapter 6 (Diseases of the nervous system)	23.4	10	**0.00923***
Mexico City	La Magdalena Contreras	Chapter 10 (Diseases of the respiratory system)	18.3	10	0.05016
Mexico City	La Magdalena Contreras	Chapter 5 (Mental and behavioural disorders)	7.3	10	0.70058
Mexico City	Milpa Alta	Chapter 9 (Diseases of the circulatory system)	34.9	10	**0.00013***
Mexico City	Milpa Alta	Chapter 4 (Endocrine, nutritional and metabolic diseases)	14.6	10	0.14656
Mexico City	Milpa Alta	Chapter 6 (Diseases of the nervous system)	12.9	10	0.22756
Mexico City	Milpa Alta	Chapter 10 (Diseases of the respiratory system)	7.4	10	0.68973
Mexico City	Milpa Alta	Chapter 5 (Mental and behavioural disorders)	13.5	10	0.19840
Mexico City	Álvaro Obregón	Chapter 9 (Diseases of the circulatory system)	11.9	10	0.29025
Mexico City	Álvaro Obregón	Chapter 4 (Endocrine, nutritional and metabolic diseases)	27.6	10	**0.00207***
Mexico City	Álvaro Obregón	Chapter 6 (Diseases of the nervous system)	9.8	10	0.45686
Mexico City	Álvaro Obregón	Chapter 10 (Diseases of the respiratory system)	31.7	10	**0.00044***
Mexico City	Álvaro Obregón	Chapter 5 (Mental and behavioural disorders)	12.2	10	0.27288
Mexico City	Tláhuac	Chapter 9 (Diseases of the circulatory system)	6.7	10	0.75128
Mexico City	Tláhuac	Chapter 4 (Endocrine, nutritional and metabolic diseases)	15.4	10	0.11810
Mexico City	Tláhuac	Chapter 6 (Diseases of the nervous system)	13.6	10	0.19022
Mexico City	Tláhuac	Chapter 10 (Diseases of the respiratory system)	14.1	10	0.16926
Mexico City	Tláhuac	Chapter 5 (Mental and behavioural disorders)	5.9	10	0.82361
Mexico City	Tlalpan	Chapter 9 (Diseases of the circulatory system)	14.1	10	0.17025
Mexico City	Tlalpan	Chapter 4 (Endocrine, nutritional and metabolic diseases)	4.4	10	0.92951
Mexico City	Tlalpan	Chapter 6 (Diseases of the nervous system)	8.2	10	0.61037
Mexico City	Tlalpan	Chapter 10 (Diseases of the respiratory system)	11.4	10	0.32510
Mexico City	Tlalpan	Chapter 5 (Mental and behavioural disorders)	15.1	10	0.13012
Mexico City	Xochimilco	Chapter 9 (Diseases of the circulatory system)	13.9	10	0.17841
Mexico City	Xochimilco	Chapter 4 (Endocrine, nutritional and metabolic diseases)	15.4	10	0.11751
Mexico City	Xochimilco	Chapter 6 (Diseases of the nervous system)	15.2	10	0.12415
Mexico City	Xochimilco	Chapter 10 (Diseases of the respiratory system)	9.2	10	0.51005
Mexico City	Xochimilco	Chapter 5 (Mental and behavioural disorders)	20.3	10	**0.02628***
Mexico City	Benito Juárez	Chapter 9 (Diseases of the circulatory system)	6.2	10	0.79606
Mexico City	Benito Juárez	Chapter 4 (Endocrine, nutritional and metabolic diseases)	3.7	10	0.96016
Mexico City	Benito Juárez	Chapter 6 (Diseases of the nervous system)	13.5	10	0.19618
Mexico City	Benito Juárez	Chapter 10 (Diseases of the respiratory system)	15.3	10	0.12306
Mexico City	Benito Juárez	Chapter 5 (Mental and behavioural disorders)	9.6	10	0.47248
Mexico City	Cuauhtémoc	Chapter 9 (Diseases of the circulatory system)	7.1	10	0.72023
Mexico City	Cuauhtémoc	Chapter 4 (Endocrine, nutritional and metabolic diseases)	15.8	10	0.10677
Mexico City	Cuauhtémoc	Chapter 6 (Diseases of the nervous system)	16.3	10	0.09034

Mexico City	Cuauhtémoc	Chapter 10 (Diseases of the respiratory system)	7.5	10	0.68027
Mexico City	Cuauhtémoc	Chapter 5 (Mental and behavioural disorders)	10.8	10	0.37363
Mexico City	Miguel Hidalgo	Chapter 9 (Diseases of the circulatory system)	14.7	10	0.14205
Mexico City	Miguel Hidalgo	Chapter 4 (Endocrine, nutritional and metabolic diseases)	12.0	10	0.28751
Mexico City	Miguel Hidalgo	Chapter 6 (Diseases of the nervous system)	12.9	10	0.22749
Mexico City	Miguel Hidalgo	Chapter 10 (Diseases of the respiratory system)	10.5	10	0.39733
Mexico City	Miguel Hidalgo	Chapter 5 (Mental and behavioural disorders)	38.3	10	**0.00003***
Mexico City	Venustiano Carranza	Chapter 9 (Diseases of the circulatory system)	17.1	10	0.07219
Mexico City	Venustiano Carranza	Chapter 4 (Endocrine, nutritional and metabolic diseases)	6.4	10	0.77979
Mexico City	Venustiano Carranza	Chapter 6 (Diseases of the nervous system)	8.7	10	0.55691
Mexico City	Venustiano Carranza	Chapter 10 (Diseases of the respiratory system)	9.1	10	0.52592
Mexico City	Venustiano Carranza	Chapter 5 (Mental and behavioural disorders)	18.8	10	**0.04269***
Hidalgo	Tizayuca	Chapter 9 (Diseases of the circulatory system)	8.4	10	0.58836
Hidalgo	Tizayuca	Chapter 4 (Endocrine, nutritional and metabolic diseases)	14.7	10	0.14504
Hidalgo	Tizayuca	Chapter 6 (Diseases of the nervous system)	7.0	10	0.72991
Hidalgo	Tizayuca	Chapter 10 (Diseases of the respiratory system)	10.8	10	0.37327
Hidalgo	Tizayuca	Chapter 5 (Mental and behavioural disorders)	8.3	10	0.60177
State of Mexico	Acolman	Chapter 9 (Diseases of the circulatory system)	30.6	10	**0.00068***
State of Mexico	Acolman	Chapter 4 (Endocrine, nutritional and metabolic diseases)	12.7	10	0.23934
State of Mexico	Acolman	Chapter 6 (Diseases of the nervous system)	10.7	10	0.37736
State of Mexico	Acolman	Chapter 10 (Diseases of the respiratory system)	29.1	10	**0.00119***
State of Mexico	Acolman	Chapter 5 (Mental and behavioural disorders)	8.5	10	0.58096
State of Mexico	Amecameca	Chapter 9 (Diseases of the circulatory system)	8.0	10	0.63060
State of Mexico	Amecameca	Chapter 4 (Endocrine, nutritional and metabolic diseases)	7.5	10	0.68143
State of Mexico	Amecameca	Chapter 6 (Diseases of the nervous system)	9.7	10	0.46564
State of Mexico	Amecameca	Chapter 10 (Diseases of the respiratory system)	5.7	10	0.84207
State of Mexico	Amecameca	Chapter 5 (Mental and behavioural disorders)	7.2	10	0.70367
State of Mexico	Apaxco	Chapter 9 (Diseases of the circulatory system)	14.7	10	0.14487
State of Mexico	Apaxco	Chapter 4 (Endocrine, nutritional and metabolic diseases)	6.0	10	0.81149
State of Mexico	Apaxco	Chapter 6 (Diseases of the nervous system)	8.2	10	0.61416
State of Mexico	Apaxco	Chapter 10 (Diseases of the respiratory system)	17.7	10	0.06029
State of Mexico	Apaxco	Chapter 5 (Mental and behavioural disorders)	1.5	10	0.99908
State of Mexico	Atenco	Chapter 9 (Diseases of the circulatory system)	13.3	10	0.20794
State of Mexico	Atenco	Chapter 4 (Endocrine, nutritional and metabolic diseases)	6.2	10	0.79849
State of Mexico	Atenco	Chapter 6 (Diseases of the nervous system)	13.9	10	0.17618
State of Mexico	Atenco	Chapter 10 (Diseases of the respiratory system)	13.1	10	0.21952
State of Mexico	Atenco	Chapter 5 (Mental and behavioural disorders)	16.0	9	0.06596
State of Mexico	Atizapán de Zaragoza	Chapter 9 (Diseases of the circulatory system)	9.7	10	0.46711
State of Mexico	Atizapán de Zaragoza	Chapter 4 (Endocrine, nutritional and metabolic diseases)	10.7	10	0.38226
State of Mexico	Atizapán de Zaragoza	Chapter 6 (Diseases of the nervous system)	10.1	10	0.43196
State of Mexico	Atizapán de Zaragoza	Chapter 10 (Diseases of the respiratory system)	7.5	10	0.67583
State of Mexico	Atizapán de Zaragoza	Chapter 5 (Mental and behavioural disorders)	15.0	10	0.13347
State of Mexico	Atlautla	Chapter 9 (Diseases of the circulatory system)	7.0	10	0.72633

State of Mexico	Atlautla	Chapter 4 (Endocrine, nutritional and metabolic diseases)	13.7	10	0.18679
State of Mexico	Atlautla	Chapter 6 (Diseases of the nervous system)	6.4	10	0.77922
State of Mexico	Atlautla	Chapter 10 (Diseases of the respiratory system)	10.1	10	0.43461
State of Mexico	Atlautla	Chapter 5 (Mental and behavioural disorders)	4.9	7	0.66790
State of Mexico	Axapusco	Chapter 9 (Diseases of the circulatory system)	33.6	10	**0.00022***
State of Mexico	Axapusco	Chapter 4 (Endocrine, nutritional and metabolic diseases)	8.9	10	0.53912
State of Mexico	Axapusco	Chapter 6 (Diseases of the nervous system)	8.3	10	0.59767
State of Mexico	Axapusco	Chapter 10 (Diseases of the respiratory system)	16.7	10	0.08090
State of Mexico	Axapusco	Chapter 5 (Mental and behavioural disorders)	10.5	10	0.40079
State of Mexico	Ayapango	Chapter 9 (Diseases of the circulatory system)	10.3	10	0.41388
State of Mexico	Ayapango	Chapter 4 (Endocrine, nutritional and metabolic diseases)	10.0	10	0.43717
State of Mexico	Ayapango	Chapter 6 (Diseases of the nervous system)	5.8	10	0.82893
State of Mexico	Ayapango	Chapter 10 (Diseases of the respiratory system)	3.1	10	0.97776
State of Mexico	Coacalco de Berriozábal	Chapter 9 (Diseases of the circulatory system)	11.7	10	0.30266
State of Mexico	Coacalco de Berriozábal	Chapter 4 (Endocrine, nutritional and metabolic diseases)	24.0	10	**0.00764***
State of Mexico	Coacalco de Berriozábal	Chapter 6 (Diseases of the nervous system)	16.1	10	0.09695
State of Mexico	Coacalco de Berriozábal	Chapter 10 (Diseases of the respiratory system)	7.9	10	0.63912
State of Mexico	Coacalco de Berriozábal	Chapter 5 (Mental and behavioural disorders)	14.8	10	0.13942
State of Mexico	Cocotitlán	Chapter 9 (Diseases of the circulatory system)	6.8	10	0.74177
State of Mexico	Cocotitlán	Chapter 4 (Endocrine, nutritional and metabolic diseases)	5.4	10	0.86409
State of Mexico	Cocotitlán	Chapter 6 (Diseases of the nervous system)	7.4	10	0.68929
State of Mexico	Cocotitlán	Chapter 10 (Diseases of the respiratory system)	14.6	10	0.14552
State of Mexico	Cocotitlán	Chapter 5 (Mental and behavioural disorders)	3.3	4	0.51105
State of Mexico	Coyotepec	Chapter 9 (Diseases of the circulatory system)	11.7	10	0.30777
State of Mexico	Coyotepec	Chapter 4 (Endocrine, nutritional and metabolic diseases)	4.9	10	0.89666
State of Mexico	Coyotepec	Chapter 6 (Diseases of the nervous system)	8.4	10	0.59298
State of Mexico	Coyotepec	Chapter 10 (Diseases of the respiratory system)	10.7	10	0.38166
State of Mexico	Coyotepec	Chapter 5 (Mental and behavioural disorders)	9.9	6	0.12993
State of Mexico	Cuautitlán	Chapter 9 (Diseases of the circulatory system)	43.8	10	**0.00000***
State of Mexico	Cuautitlán	Chapter 4 (Endocrine, nutritional and metabolic diseases)	5.1	10	0.88534
State of Mexico	Cuautitlán	Chapter 6 (Diseases of the nervous system)	5.0	10	0.89230
State of Mexico	Cuautitlán	Chapter 10 (Diseases of the respiratory system)	47.7	10	**0.00000***
State of Mexico	Cuautitlán	Chapter 5 (Mental and behavioural disorders)	9.5	10	0.48340
State of Mexico	Chalco	Chapter 9 (Diseases of the circulatory system)	9.7	10	0.46356
State of Mexico	Chalco	Chapter 4 (Endocrine, nutritional and metabolic diseases)	12.8	10	0.23645
State of Mexico	Chalco	Chapter 6 (Diseases of the nervous system)	10.2	10	0.42703
State of Mexico	Chalco	Chapter 10 (Diseases of the respiratory system)	10.3	10	0.41470
State of Mexico	Chalco	Chapter 5 (Mental and behavioural disorders)	20.9	10	**0.02206***
State of Mexico	Chiautla	Chapter 9 (Diseases of the circulatory system)	7.1	10	0.71661
State of Mexico	Chiautla	Chapter 4 (Endocrine, nutritional and metabolic diseases)	15.4	10	0.11890
State of Mexico	Chiautla	Chapter 6 (Diseases of the nervous system)	5.4	10	0.86075
State of Mexico	Chiautla	Chapter 10 (Diseases of the respiratory system)	10.4	10	0.40719
State of Mexico	Chiautla	Chapter 5 (Mental and behavioural disorders)	1.6	10	0.99849

State of Mexico	Chicoloapan	Chapter 9 (Diseases of the circulatory system)	7.0	10	0.72988
State of Mexico	Chicoloapan	Chapter 4 (Endocrine, nutritional and metabolic diseases)	9.9	10	0.44556
State of Mexico	Chicoloapan	Chapter 6 (Diseases of the nervous system)	8.4	10	0.59343
State of Mexico	Chicoloapan	Chapter 10 (Diseases of the respiratory system)	7.7	10	0.66283
State of Mexico	Chicoloapan	Chapter 5 (Mental and behavioural disorders)	7.0	10	0.72124
State of Mexico	Chiconcuac	Chapter 9 (Diseases of the circulatory system)	7.5	10	0.67271
State of Mexico	Chiconcuac	Chapter 4 (Endocrine, nutritional and metabolic diseases)	13.7	10	0.18708
State of Mexico	Chiconcuac	Chapter 6 (Diseases of the nervous system)	2.8	10	0.98596
State of Mexico	Chiconcuac	Chapter 10 (Diseases of the respiratory system)	19.8	10	**0.03151***
State of Mexico	Chiconcuac	Chapter 5 (Mental and behavioural disorders)	9.0	7	0.25448
State of Mexico	Chimalhuacán	Chapter 9 (Diseases of the circulatory system)	11.5	10	0.32275
State of Mexico	Chimalhuacán	Chapter 4 (Endocrine, nutritional and metabolic diseases)	8.0	10	0.62429
State of Mexico	Chimalhuacán	Chapter 6 (Diseases of the nervous system)	8.2	10	0.61138
State of Mexico	Chimalhuacán	Chapter 10 (Diseases of the respiratory system)	13.0	10	0.22516
State of Mexico	Chimalhuacán	Chapter 5 (Mental and behavioural disorders)	21.6	10	**0.01708***
State of Mexico	Ecatepec de Morelos	Chapter 9 (Diseases of the circulatory system)	17.9	10	0.05754
State of Mexico	Ecatepec de Morelos	Chapter 4 (Endocrine, nutritional and metabolic diseases)	5.7	10	0.84011
State of Mexico	Ecatepec de Morelos	Chapter 6 (Diseases of the nervous system)	18.2	10	0.05127
State of Mexico	Ecatepec de Morelos	Chapter 10 (Diseases of the respiratory system)	5.9	10	0.82269
State of Mexico	Ecatepec de Morelos	Chapter 5 (Mental and behavioural disorders)	9.2	10	0.51389
State of Mexico	Ecatzingo	Chapter 9 (Diseases of the circulatory system)	10.7	10	0.38120
State of Mexico	Ecatzingo	Chapter 4 (Endocrine, nutritional and metabolic diseases)	5.0	10	0.89209
State of Mexico	Ecatzingo	Chapter 6 (Diseases of the nervous system)	1.9	6	0.92499
State of Mexico	Ecatzingo	Chapter 10 (Diseases of the respiratory system)	10.0	10	0.44268
State of Mexico	Huehuetoca	Chapter 9 (Diseases of the circulatory system)	11.9	10	0.29196
State of Mexico	Huehuetoca	Chapter 4 (Endocrine, nutritional and metabolic diseases)	10.5	10	0.40142
State of Mexico	Huehuetoca	Chapter 6 (Diseases of the nervous system)	19.8	10	**0.03072***
State of Mexico	Huehuetoca	Chapter 10 (Diseases of the respiratory system)	8.8	10	0.55382
State of Mexico	Huehuetoca	Chapter 5 (Mental and behavioural disorders)	5.4	10	0.86285
State of Mexico	Hueypoxtla	Chapter 9 (Diseases of the circulatory system)	7.3	10	0.69657
State of Mexico	Hueypoxtla	Chapter 4 (Endocrine, nutritional and metabolic diseases)	17.4	10	0.06573
State of Mexico	Hueypoxtla	Chapter 6 (Diseases of the nervous system)	8.0	10	0.62891
State of Mexico	Hueypoxtla	Chapter 10 (Diseases of the respiratory system)	13.0	10	0.22443
State of Mexico	Hueypoxtla	Chapter 5 (Mental and behavioural disorders)	9.1	10	0.52307
State of Mexico	Huixquilucan	Chapter 9 (Diseases of the circulatory system)	41.6	10	**0.00001***
State of Mexico	Huixquilucan	Chapter 4 (Endocrine, nutritional and metabolic diseases)	14.2	10	0.16488
State of Mexico	Huixquilucan	Chapter 6 (Diseases of the nervous system)	7.7	10	0.65841
State of Mexico	Huixquilucan	Chapter 10 (Diseases of the respiratory system)	11.9	10	0.29169
State of Mexico	Huixquilucan	Chapter 5 (Mental and behavioural disorders)	12.9	10	0.23205
State of Mexico	Isidro Fabela	Chapter 9 (Diseases of the circulatory system)	8.1	10	0.62168
State of Mexico	Isidro Fabela	Chapter 4 (Endocrine, nutritional and metabolic diseases)	5.6	10	0.85071
State of Mexico	Isidro Fabela	Chapter 6 (Diseases of the nervous system)	15.9	8	**0.04390***
State of Mexico	Isidro Fabela	Chapter 10 (Diseases of the respiratory system)	5.8	10	0.83572

State of Mexico	Ixtapaluca	Chapter 9 (Diseases of the circulatory system)	23.7	10	**0.00837***
State of Mexico	Ixtapaluca	Chapter 4 (Endocrine, nutritional and metabolic diseases)	8.0	10	0.62783
State of Mexico	Ixtapaluca	Chapter 6 (Diseases of the nervous system)	7.6	10	0.66421
State of Mexico	Ixtapaluca	Chapter 10 (Diseases of the respiratory system)	16.6	10	0.08296
State of Mexico	Ixtapaluca	Chapter 5 (Mental and behavioural disorders)	4.8	10	0.90202
State of Mexico	Jaltenco	Chapter 9 (Diseases of the circulatory system)	17.1	10	0.07283
State of Mexico	Jaltenco	Chapter 4 (Endocrine, nutritional and metabolic diseases)	20.5	10	**0.02449***
State of Mexico	Jaltenco	Chapter 6 (Diseases of the nervous system)	12.9	10	0.22915
State of Mexico	Jaltenco	Chapter 10 (Diseases of the respiratory system)	8.1	10	0.61419
State of Mexico	Jilotzingo	Chapter 9 (Diseases of the circulatory system)	7.0	10	0.72256
State of Mexico	Jilotzingo	Chapter 4 (Endocrine, nutritional and metabolic diseases)	13.0	10	0.22605
State of Mexico	Jilotzingo	Chapter 6 (Diseases of the nervous system)	11.1	10	0.35004
State of Mexico	Jilotzingo	Chapter 10 (Diseases of the respiratory system)	11.2	10	0.33889
State of Mexico	Jilotzingo	Chapter 5 (Mental and behavioural disorders)	9.1	6	0.16862
State of Mexico	Juchitepec	Chapter 9 (Diseases of the circulatory system)	10.0	10	0.44480
State of Mexico	Juchitepec	Chapter 4 (Endocrine, nutritional and metabolic diseases)	8.7	10	0.56310
State of Mexico	Juchitepec	Chapter 6 (Diseases of the nervous system)	19.3	10	**0.03704***
State of Mexico	Juchitepec	Chapter 10 (Diseases of the respiratory system)	4.3	10	0.93300
State of Mexico	Juchitepec	Chapter 5 (Mental and behavioural disorders)	3.2	6	0.78437
State of Mexico	Melchor Ocampo	Chapter 9 (Diseases of the circulatory system)	12.9	10	0.22946
State of Mexico	Melchor Ocampo	Chapter 4 (Endocrine, nutritional and metabolic diseases)	16.7	10	0.08175
State of Mexico	Melchor Ocampo	Chapter 6 (Diseases of the nervous system)	8.9	10	0.54186
State of Mexico	Melchor Ocampo	Chapter 10 (Diseases of the respiratory system)	7.0	10	0.72788
State of Mexico	Melchor Ocampo	Chapter 5 (Mental and behavioural disorders)	10.8	8	0.21167
State of Mexico	Naucalpan de Juárez	Chapter 9 (Diseases of the circulatory system)	16.1	10	0.09718
State of Mexico	Naucalpan de Juárez	Chapter 4 (Endocrine, nutritional and metabolic diseases)	22.5	10	**0.01281***
State of Mexico	Naucalpan de Juárez	Chapter 6 (Diseases of the nervous system)	10.0	10	0.44241
State of Mexico	Naucalpan de Juárez	Chapter 10 (Diseases of the respiratory system)	11.3	10	0.33217
State of Mexico	Naucalpan de Juárez	Chapter 5 (Mental and behavioural disorders)	16.2	10	0.09317
State of Mexico	Nezahualcóyotl	Chapter 9 (Diseases of the circulatory system)	15.4	10	0.11705
State of Mexico	Nezahualcóyotl	Chapter 4 (Endocrine, nutritional and metabolic diseases)	15.8	10	0.10627
State of Mexico	Nezahualcóyotl	Chapter 6 (Diseases of the nervous system)	7.2	10	0.70599
State of Mexico	Nezahualcóyotl	Chapter 10 (Diseases of the respiratory system)	19.5	10	**0.03412***
State of Mexico	Nezahualcóyotl	Chapter 5 (Mental and behavioural disorders)	6.1	10	0.80408
State of Mexico	Nextlalpan	Chapter 9 (Diseases of the circulatory system)	7.9	10	0.64344
State of Mexico	Nextlalpan	Chapter 4 (Endocrine, nutritional and metabolic diseases)	8.0	10	0.63177
State of Mexico	Nextlalpan	Chapter 6 (Diseases of the nervous system)	6.4	6	0.37849
State of Mexico	Nextlalpan	Chapter 10 (Diseases of the respiratory system)	7.6	10	0.66685
State of Mexico	Nicolás Romero	Chapter 9 (Diseases of the circulatory system)	11.0	10	0.35554
State of Mexico	Nicolás Romero	Chapter 4 (Endocrine, nutritional and metabolic diseases)	11.2	10	0.34234
State of Mexico	Nicolás Romero	Chapter 6 (Diseases of the nervous system)	10.4	10	0.40913
State of Mexico	Nicolás Romero	Chapter 10 (Diseases of the respiratory system)	18.4	10	0.04886
State of Mexico	Nicolás Romero	Chapter 5 (Mental and behavioural disorders)	7.7	10	0.65919

State of Mexico	Nopaltepec	Chapter 9 (Diseases of the circulatory system)	13.8	10	0.18241
State of Mexico	Nopaltepec	Chapter 4 (Endocrine, nutritional and metabolic diseases)	28.2	10	**0.00166***
State of Mexico	Nopaltepec	Chapter 6 (Diseases of the nervous system)	2.3	4	0.68473
State of Mexico	Nopaltepec	Chapter 10 (Diseases of the respiratory system)	10.5	10	0.39799
State of Mexico	Otumba	Chapter 9 (Diseases of the circulatory system)	6.9	10	0.73126
State of Mexico	Otumba	Chapter 4 (Endocrine, nutritional and metabolic diseases)	13.0	10	0.22255
State of Mexico	Otumba	Chapter 6 (Diseases of the nervous system)	12.5	10	0.25027
State of Mexico	Otumba	Chapter 10 (Diseases of the respiratory system)	18.5	10	0.04726
State of Mexico	Otumba	Chapter 5 (Mental and behavioural disorders)	4.9	10	0.89933
State of Mexico	Ozumba	Chapter 9 (Diseases of the circulatory system)	6.7	10	0.74896
State of Mexico	Ozumba	Chapter 4 (Endocrine, nutritional and metabolic diseases)	6.3	10	0.78945
State of Mexico	Ozumba	Chapter 6 (Diseases of the nervous system)	11.0	10	0.36119
State of Mexico	Ozumba	Chapter 10 (Diseases of the respiratory system)	17.6	10	0.06130
State of Mexico	Ozumba	Chapter 5 (Mental and behavioural disorders)	3.5	10	0.96691
State of Mexico	Papalotla	Chapter 9 (Diseases of the circulatory system)	5.1	10	0.88568
State of Mexico	Papalotla	Chapter 4 (Endocrine, nutritional and metabolic diseases)	7.8	10	0.64925
State of Mexico	Papalotla	Chapter 6 (Diseases of the nervous system)	10.2	7	0.17490
State of Mexico	Papalotla	Chapter 10 (Diseases of the respiratory system)	11.9	10	0.28879
State of Mexico	La Paz	Chapter 9 (Diseases of the circulatory system)	11.4	10	0.32429
State of Mexico	La Paz	Chapter 4 (Endocrine, nutritional and metabolic diseases)	7.0	10	0.72599
State of Mexico	La Paz	Chapter 6 (Diseases of the nervous system)	8.1	10	0.61786
State of Mexico	La Paz	Chapter 10 (Diseases of the respiratory system)	13.4	10	0.20406
State of Mexico	La Paz	Chapter 5 (Mental and behavioural disorders)	14.1	10	0.16715
State of Mexico	San Martín de las Pirámides	Chapter 9 (Diseases of the circulatory system)	17.0	10	0.07441
State of Mexico	San Martín de las Pirámides	Chapter 4 (Endocrine, nutritional and metabolic diseases)	11.0	10	0.35377
State of Mexico	San Martín de las Pirámides	Chapter 6 (Diseases of the nervous system)	14.1	10	0.16812
State of Mexico	San Martín de las Pirámides	Chapter 10 (Diseases of the respiratory system)	7.0	10	0.72628
State of Mexico	San Martín de las Pirámides	Chapter 5 (Mental and behavioural disorders)	3.4	6	0.76341
State of Mexico	Tecámac	Chapter 9 (Diseases of the circulatory system)	26.6	10	**0.00299***
State of Mexico	Tecámac	Chapter 4 (Endocrine, nutritional and metabolic diseases)	9.5	10	0.48410
State of Mexico	Tecámac	Chapter 6 (Diseases of the nervous system)	15.6	10	0.11161
State of Mexico	Tecámac	Chapter 10 (Diseases of the respiratory system)	14.9	10	0.13695
State of Mexico	Tecámac	Chapter 5 (Mental and behavioural disorders)	6.2	10	0.79498
State of Mexico	Temamatla	Chapter 9 (Diseases of the circulatory system)	6.8	10	0.74569
State of Mexico	Temamatla	Chapter 4 (Endocrine, nutritional and metabolic diseases)	4.8	10	0.90287
State of Mexico	Temamatla	Chapter 10 (Diseases of the respiratory system)	6.1	10	0.80818
State of Mexico	Temascalapa	Chapter 9 (Diseases of the circulatory system)	10.3	10	0.41172
State of Mexico	Temascalapa	Chapter 4 (Endocrine, nutritional and metabolic diseases)	18.6	10	**0.04619***
State of Mexico	Temascalapa	Chapter 6 (Diseases of the nervous system)	17.2	10	0.07011
State of Mexico	Temascalapa	Chapter 10 (Diseases of the respiratory system)	7.4	10	0.69162
State of Mexico	Temascalapa	Chapter 5 (Mental and behavioural disorders)	6.0	5	0.30531
State of Mexico	Tenango del Aire	Chapter 9 (Diseases of the circulatory system)	8.0	10	0.63283
State of Mexico	Tenango del Aire	Chapter 4 (Endocrine, nutritional and metabolic diseases)	5.9	10	0.82281

State of Mexico	Tenango del Aire	Chapter 6 (Diseases of the nervous system)	11.7	6	0.06874
State of Mexico	Tenango del Aire	Chapter 10 (Diseases of the respiratory system)	37.7	10	**0.00004***
State of Mexico	Teoloyucan	Chapter 9 (Diseases of the circulatory system)	6.4	10	0.77855
State of Mexico	Teoloyucan	Chapter 4 (Endocrine, nutritional and metabolic diseases)	11.6	10	0.31473
State of Mexico	Teoloyucan	Chapter 6 (Diseases of the nervous system)	4.7	10	0.90743
State of Mexico	Teoloyucan	Chapter 10 (Diseases of the respiratory system)	5.9	10	0.82421
State of Mexico	Teoloyucan	Chapter 5 (Mental and behavioural disorders)	18.1	10	0.05282
State of Mexico	Teotihuacán	Chapter 9 (Diseases of the circulatory system)	9.7	10	0.46356
State of Mexico	Teotihuacán	Chapter 4 (Endocrine, nutritional and metabolic diseases)	18.6	10	**0.04546***
State of Mexico	Teotihuacán	Chapter 6 (Diseases of the nervous system)	11.2	10	0.34073
State of Mexico	Teotihuacán	Chapter 10 (Diseases of the respiratory system)	9.5	10	0.48250
State of Mexico	Teotihuacán	Chapter 5 (Mental and behavioural disorders)	11.0	9	0.27872
State of Mexico	Tepetlaoxtoc	Chapter 9 (Diseases of the circulatory system)	19.3	10	**0.03707***
State of Mexico	Tepetlaoxtoc	Chapter 4 (Endocrine, nutritional and metabolic diseases)	11.4	10	0.32746
State of Mexico	Tepetlaoxtoc	Chapter 6 (Diseases of the nervous system)	8.4	10	0.59427
State of Mexico	Tepetlaoxtoc	Chapter 10 (Diseases of the respiratory system)	15.9	10	0.10332
State of Mexico	Tepetlaoxtoc	Chapter 5 (Mental and behavioural disorders)	4.5	7	0.71838
State of Mexico	Tepetlixpa	Chapter 9 (Diseases of the circulatory system)	10.2	10	0.41992
State of Mexico	Tepetlixpa	Chapter 4 (Endocrine, nutritional and metabolic diseases)	6.1	10	0.80552
State of Mexico	Tepetlixpa	Chapter 6 (Diseases of the nervous system)	14.3	10	0.15796
State of Mexico	Tepetlixpa	Chapter 10 (Diseases of the respiratory system)	10.1	10	0.43409
State of Mexico	Tepetlixpa	Chapter 5 (Mental and behavioural disorders)	7.5	10	0.68198
State of Mexico	Tepotzotlán	Chapter 9 (Diseases of the circulatory system)	33.8	10	**0.00020***
State of Mexico	Tepotzotlán	Chapter 4 (Endocrine, nutritional and metabolic diseases)	4.6	10	0.91848
State of Mexico	Tepotzotlán	Chapter 6 (Diseases of the nervous system)	7.2	10	0.70485
State of Mexico	Tepotzotlán	Chapter 10 (Diseases of the respiratory system)	12.6	10	0.24657
State of Mexico	Tepotzotlán	Chapter 5 (Mental and behavioural disorders)	8.4	10	0.59426
State of Mexico	Tequixquiac	Chapter 9 (Diseases of the circulatory system)	11.6	10	0.31286
State of Mexico	Tequixquiac	Chapter 4 (Endocrine, nutritional and metabolic diseases)	7.1	10	0.71876
State of Mexico	Tequixquiac	Chapter 6 (Diseases of the nervous system)	17.0	10	0.07482
State of Mexico	Tequixquiac	Chapter 10 (Diseases of the respiratory system)	5.7	10	0.83821
State of Mexico	Tequixquiac	Chapter 5 (Mental and behavioural disorders)	9.0	6	0.17488
State of Mexico	Texcoco	Chapter 9 (Diseases of the circulatory system)	13.2	10	0.21462
State of Mexico	Texcoco	Chapter 4 (Endocrine, nutritional and metabolic diseases)	7.3	10	0.69324
State of Mexico	Texcoco	Chapter 6 (Diseases of the nervous system)	11.3	10	0.33823
State of Mexico	Texcoco	Chapter 10 (Diseases of the respiratory system)	27.4	10	**0.00229***
State of Mexico	Texcoco	Chapter 5 (Mental and behavioural disorders)	13.5	10	0.19477
State of Mexico	Tezoyuca	Chapter 9 (Diseases of the circulatory system)	5.6	10	0.84950
State of Mexico	Tezoyuca	Chapter 4 (Endocrine, nutritional and metabolic diseases)	8.2	10	0.61148
State of Mexico	Tezoyuca	Chapter 6 (Diseases of the nervous system)	12.5	10	0.25346
State of Mexico	Tezoyuca	Chapter 10 (Diseases of the respiratory system)	10.9	10	0.36730
State of Mexico	Tezoyuca	Chapter 5 (Mental and behavioural disorders)	8.7	8	0.36675
State of Mexico	Tlalmanalco	Chapter 9 (Diseases of the circulatory system)	11.4	10	0.32402

State of Mexico	Tlalmanalco	Chapter 4 (Endocrine, nutritional and metabolic diseases)	7.6	10	0.66560
State of Mexico	Tlalmanalco	Chapter 6 (Diseases of the nervous system)	11.7	10	0.30380
State of Mexico	Tlalmanalco	Chapter 10 (Diseases of the respiratory system)	10.2	10	0.42394
State of Mexico	Tlalmanalco	Chapter 5 (Mental and behavioural disorders)	17.3	10	0.06890
State of Mexico	Tlalnepantla de Baz	Chapter 9 (Diseases of the circulatory system)	19.1	10	**0.03962***
State of Mexico	Tlalnepantla de Baz	Chapter 4 (Endocrine, nutritional and metabolic diseases)	6.7	10	0.75108
State of Mexico	Tlalnepantla de Baz	Chapter 6 (Diseases of the nervous system)	6.8	10	0.74615
State of Mexico	Tlalnepantla de Baz	Chapter 10 (Diseases of the respiratory system)	23.5	10	**0.00909***
State of Mexico	Tlalnepantla de Baz	Chapter 5 (Mental and behavioural disorders)	6.5	10	0.77213
State of Mexico	Tultepec	Chapter 9 (Diseases of the circulatory system)	4.4	10	0.92917
State of Mexico	Tultepec	Chapter 4 (Endocrine, nutritional and metabolic diseases)	10.8	10	0.37644
State of Mexico	Tultepec	Chapter 6 (Diseases of the nervous system)	16.0	10	0.09867
State of Mexico	Tultepec	Chapter 10 (Diseases of the respiratory system)	9.2	10	0.51178
State of Mexico	Tultepec	Chapter 5 (Mental and behavioural disorders)	12.5	10	0.25010
State of Mexico	Tultitlán	Chapter 9 (Diseases of the circulatory system)	6.5	10	0.77336
State of Mexico	Tultitlán	Chapter 4 (Endocrine, nutritional and metabolic diseases)	17.7	10	0.06088
State of Mexico	Tultitlán	Chapter 6 (Diseases of the nervous system)	15.9	10	0.10262
State of Mexico	Tultitlán	Chapter 10 (Diseases of the respiratory system)	11.4	10	0.32370
State of Mexico	Tultitlán	Chapter 5 (Mental and behavioural disorders)	10.8	10	0.37232
State of Mexico	Villa del Carbón	Chapter 9 (Diseases of the circulatory system)	11.3	10	0.33625
State of Mexico	Villa del Carbón	Chapter 4 (Endocrine, nutritional and metabolic diseases)	14.5	10	0.15165
State of Mexico	Villa del Carbón	Chapter 6 (Diseases of the nervous system)	13.0	10	0.22382
State of Mexico	Villa del Carbón	Chapter 10 (Diseases of the respiratory system)	5.6	10	0.84570
State of Mexico	Villa del Carbón	Chapter 5 (Mental and behavioural disorders)	12.2	10	0.26992
State of Mexico	Zumpango	Chapter 9 (Diseases of the circulatory system)	13.0	10	0.22100
State of Mexico	Zumpango	Chapter 4 (Endocrine, nutritional and metabolic diseases)	7.6	10	0.67195
State of Mexico	Zumpango	Chapter 6 (Diseases of the nervous system)	11.3	10	0.33501
State of Mexico	Zumpango	Chapter 10 (Diseases of the respiratory system)	5.3	10	0.87051
State of Mexico	Zumpango	Chapter 5 (Mental and behavioural disorders)	14.7	10	0.14529
State of Mexico	Cuautitlán Izcalli	Chapter 9 (Diseases of the circulatory system)	21.5	10	**0.01771***
State of Mexico	Cuautitlán Izcalli	Chapter 4 (Endocrine, nutritional and metabolic diseases)	9.5	10	0.48934
State of Mexico	Cuautitlán Izcalli	Chapter 6 (Diseases of the nervous system)	11.6	10	0.31451
State of Mexico	Cuautitlán Izcalli	Chapter 10 (Diseases of the respiratory system)	11.5	10	0.32329
State of Mexico	Cuautitlán Izcalli	Chapter 5 (Mental and behavioural disorders)	9.3	10	0.50524
State of Mexico	Valle de Chalco Solidaridad	Chapter 9 (Diseases of the circulatory system)	18.0	10	0.05466
State of Mexico	Valle de Chalco Solidaridad	Chapter 4 (Endocrine, nutritional and metabolic diseases)	11.9	10	0.28935
State of Mexico	Valle de Chalco Solidaridad	Chapter 6 (Diseases of the nervous system)	9.5	10	0.48143
State of Mexico	Valle de Chalco Solidaridad	Chapter 10 (Diseases of the respiratory system)	20.8	10	**0.02260***
State of Mexico	Valle de Chalco Solidaridad	Chapter 5 (Mental and behavioural disorders)	8.2	10	0.61399
State of Mexico	Tonanitla	Chapter 9 (Diseases of the circulatory system)	7.4	10	0.69145
State of Mexico	Tonanitla	Chapter 4 (Endocrine, nutritional and metabolic diseases)	11.0	10	0.36105
State of Mexico	Tonanitla	Chapter 10 (Diseases of the respiratory system)	10.3	10	0.41843

Table S5. Ljung–Box test results for residual autocorrelation by ICD-10 chapter and sex at the municipality level. (*) indicates statistically significant autocorrelation.

State	Municipality	ICD-10 Chapter	Sex	Ljung–Box *Q**	*df*	*p-value*
Mexico City	Azcapotzalco	Chapter 9 (Diseases of the circulatory system)	Men	14.7	10	0.1438
Mexico City	Azcapotzalco	Chapter 9 (Diseases of the circulatory system)	Women	4.5	10	0.9223
Mexico City	Azcapotzalco	Chapter 4 (Endocrine, nutritional and metabolic diseases)	Men	7.2	10	0.7072
Mexico City	Azcapotzalco	Chapter 4 (Endocrine, nutritional and metabolic diseases)	Women	14.0	10	0.1711
Mexico City	Azcapotzalco	Chapter 6 (Diseases of the nervous system)	Men	7.6	10	0.6705
Mexico City	Azcapotzalco	Chapter 6 (Diseases of the nervous system)	Women	9.0	10	0.5307
Mexico City	Azcapotzalco	Chapter 10 (Diseases of the respiratory system)	Men	7.7	10	0.6559
Mexico City	Azcapotzalco	Chapter 10 (Diseases of the respiratory system)	Women	5.1	10	0.8848
Mexico City	Azcapotzalco	Chapter 5 (Mental and behavioural disorders)	Men	7.3	10	0.6997
Mexico City	Azcapotzalco	Chapter 5 (Mental and behavioural disorders)	Women	17.3	10	0.0684
Mexico City	Coyoacán	Chapter 9 (Diseases of the circulatory system)	Men	11.1	10	0.3531
Mexico City	Coyoacán	Chapter 9 (Diseases of the circulatory system)	Women	11.3	10	0.3330
Mexico City	Coyoacán	Chapter 4 (Endocrine, nutritional and metabolic diseases)	Men	9.1	10	0.5196
Mexico City	Coyoacán	Chapter 4 (Endocrine, nutritional and metabolic diseases)	Women	8.8	10	0.5547
Mexico City	Coyoacán	Chapter 6 (Diseases of the nervous system)	Men	6.5	10	0.7757
Mexico City	Coyoacán	Chapter 6 (Diseases of the nervous system)	Women	9.7	10	0.4673
Mexico City	Coyoacán	Chapter 10 (Diseases of the respiratory system)	Men	14.9	10	0.1360
Mexico City	Coyoacán	Chapter 10 (Diseases of the respiratory system)	Women	7.2	10	0.7052
Mexico City	Coyoacán	Chapter 5 (Mental and behavioural disorders)	Men	11.0	10	0.3574
Mexico City	Coyoacán	Chapter 5 (Mental and behavioural disorders)	Women	10.2	10	0.4252
Mexico City	Cuajimalpa de Morelos	Chapter 9 (Diseases of the circulatory system)	Men	21.2	10	**0.0197***
Mexico City	Cuajimalpa de Morelos	Chapter 9 (Diseases of the circulatory system)	Women	29.6	10	**0.0010***
Mexico City	Cuajimalpa de Morelos	Chapter 4 (Endocrine, nutritional and metabolic diseases)	Men	20.2	10	**0.0271***
Mexico City	Cuajimalpa de Morelos	Chapter 4 (Endocrine, nutritional and metabolic diseases)	Women	11.8	10	0.2991
Mexico City	Cuajimalpa de Morelos	Chapter 6 (Diseases of the nervous system)	Men	13.7	10	0.1866
Mexico City	Cuajimalpa de Morelos	Chapter 6 (Diseases of the nervous system)	Women	8.2	10	0.6067
Mexico City	Cuajimalpa de Morelos	Chapter 10 (Diseases of the respiratory system)	Men	4.2	10	0.9372
Mexico City	Cuajimalpa de Morelos	Chapter 10 (Diseases of the respiratory system)	Women	14.3	10	0.1586
Mexico City	Cuajimalpa de Morelos	Chapter 5 (Mental and behavioural disorders)	Men	13.3	10	0.2090
Mexico City	Cuajimalpa de Morelos	Chapter 5 (Mental and behavioural disorders)	Women	4.0	8	0.8602
Mexico City	Gustavo A. Madero	Chapter 9 (Diseases of the circulatory system)	Men	18.1	10	0.0527
Mexico City	Gustavo A. Madero	Chapter 9 (Diseases of the circulatory system)	Women	25.6	10	**0.0043***
Mexico City	Gustavo A. Madero	Chapter 4 (Endocrine, nutritional and metabolic diseases)	Men	11.4	10	0.3278
Mexico City	Gustavo A. Madero	Chapter 4 (Endocrine, nutritional and metabolic diseases)	Women	20.1	10	**0.0286***
Mexico City	Gustavo A. Madero	Chapter 6 (Diseases of the nervous system)	Men	18.2	10	0.0524
Mexico City	Gustavo A. Madero	Chapter 6 (Diseases of the nervous system)	Women	8.4	10	0.5862
Mexico City	Gustavo A. Madero	Chapter 10 (Diseases of the respiratory system)	Men	6.0	10	0.8147
Mexico City	Gustavo A. Madero	Chapter 10 (Diseases of the respiratory system)	Women	12.1	10	0.2798
Mexico City	Gustavo A. Madero	Chapter 5 (Mental and behavioural disorders)	Men	14.7	10	0.1431
Mexico City	Gustavo A. Madero	Chapter 5 (Mental and behavioural disorders)	Women	9.1	10	0.5249

Mexico City	Iztacalco	Chapter 9 (Diseases of the circulatory system)	Men	4.3	10	0.9339
Mexico City	Iztacalco	Chapter 9 (Diseases of the circulatory system)	Women	9.8	10	0.4575
Mexico City	Iztacalco	Chapter 4 (Endocrine, nutritional and metabolic diseases)	Men	14.0	10	0.1711
Mexico City	Iztacalco	Chapter 4 (Endocrine, nutritional and metabolic diseases)	Women	7.1	10	0.7155
Mexico City	Iztacalco	Chapter 6 (Diseases of the nervous system)	Men	8.6	10	0.5682
Mexico City	Iztacalco	Chapter 6 (Diseases of the nervous system)	Women	9.4	10	0.4920
Mexico City	Iztacalco	Chapter 10 (Diseases of the respiratory system)	Men	6.9	10	0.7304
Mexico City	Iztacalco	Chapter 10 (Diseases of the respiratory system)	Women	9.0	10	0.5366
Mexico City	Iztacalco	Chapter 5 (Mental and behavioural disorders)	Men	16.5	10	0.0862
Mexico City	Iztacalco	Chapter 5 (Mental and behavioural disorders)	Women	8.5	10	0.5777
Mexico City	Iztapalapa	Chapter 9 (Diseases of the circulatory system)	Men	19.4	10	**0.0356***
Mexico City	Iztapalapa	Chapter 9 (Diseases of the circulatory system)	Women	13.2	10	0.2123
Mexico City	Iztapalapa	Chapter 4 (Endocrine, nutritional and metabolic diseases)	Men	10.3	10	0.4161
Mexico City	Iztapalapa	Chapter 4 (Endocrine, nutritional and metabolic diseases)	Women	10.1	10	0.4351
Mexico City	Iztapalapa	Chapter 6 (Diseases of the nervous system)	Men	14.0	10	0.1746
Mexico City	Iztapalapa	Chapter 6 (Diseases of the nervous system)	Women	21.1	10	**0.0203***
Mexico City	Iztapalapa	Chapter 10 (Diseases of the respiratory system)	Men	16.8	10	0.0798
Mexico City	Iztapalapa	Chapter 10 (Diseases of the respiratory system)	Women	13.4	10	0.2027
Mexico City	Iztapalapa	Chapter 5 (Mental and behavioural disorders)	Men	16.4	10	0.0889
Mexico City	Iztapalapa	Chapter 5 (Mental and behavioural disorders)	Women	6.4	10	0.7802
Mexico City	La Magdalena Contreras	Chapter 9 (Diseases of the circulatory system)	Men	36.3	10	**0.0001***
Mexico City	La Magdalena Contreras	Chapter 9 (Diseases of the circulatory system)	Women	15.4	10	0.1188
Mexico City	La Magdalena Contreras	Chapter 4 (Endocrine, nutritional and metabolic diseases)	Men	8.0	10	0.6283
Mexico City	La Magdalena Contreras	Chapter 4 (Endocrine, nutritional and metabolic diseases)	Women	5.3	10	0.8713
Mexico City	La Magdalena Contreras	Chapter 6 (Diseases of the nervous system)	Men	10.6	10	0.3923
Mexico City	La Magdalena Contreras	Chapter 6 (Diseases of the nervous system)	Women	15.6	10	0.1132
Mexico City	La Magdalena Contreras	Chapter 10 (Diseases of the respiratory system)	Men	11.5	10	0.3179
Mexico City	La Magdalena Contreras	Chapter 10 (Diseases of the respiratory system)	Women	16.3	10	0.0914
Mexico City	La Magdalena Contreras	Chapter 5 (Mental and behavioural disorders)	Men	7.0	10	0.7234
Mexico City	La Magdalena Contreras	Chapter 5 (Mental and behavioural disorders)	Women	9.4	10	0.4978
Mexico City	Milpa Alta	Chapter 9 (Diseases of the circulatory system)	Men	11.7	10	0.3022
Mexico City	Milpa Alta	Chapter 9 (Diseases of the circulatory system)	Women	21.7	10	**0.0168***
Mexico City	Milpa Alta	Chapter 4 (Endocrine, nutritional and metabolic diseases)	Men	17.5	10	0.0637
Mexico City	Milpa Alta	Chapter 4 (Endocrine, nutritional and metabolic diseases)	Women	10.2	10	0.4260
Mexico City	Milpa Alta	Chapter 6 (Diseases of the nervous system)	Men	6.2	10	0.7947
Mexico City	Milpa Alta	Chapter 6 (Diseases of the nervous system)	Women	11.3	10	0.3343
Mexico City	Milpa Alta	Chapter 10 (Diseases of the respiratory system)	Men	8.6	10	0.5663
Mexico City	Milpa Alta	Chapter 10 (Diseases of the respiratory system)	Women	13.3	10	0.2064
Mexico City	Milpa Alta	Chapter 5 (Mental and behavioural disorders)	Men	9.1	10	0.5197
Mexico City	Milpa Alta	Chapter 5 (Mental and behavioural disorders)	Women	7.1	4	0.1300
Mexico City	Álvaro Obregón	Chapter 9 (Diseases of the circulatory system)	Men	8.2	10	0.6099
Mexico City	Álvaro Obregón	Chapter 9 (Diseases of the circulatory system)	Women	11.5	10	0.3227
Mexico City	Álvaro Obregón	Chapter 4 (Endocrine, nutritional and metabolic diseases)	Men	34.8	10	**0.0001***

Mexico City	Álvaro Obregón	Chapter 4 (Endocrine, nutritional and metabolic diseases)	Women	11.6	10	0.3104
Mexico City	Álvaro Obregón	Chapter 6 (Diseases of the nervous system)	Men	7.7	10	0.6573
Mexico City	Álvaro Obregón	Chapter 6 (Diseases of the nervous system)	Women	7.3	10	0.6943
Mexico City	Álvaro Obregón	Chapter 10 (Diseases of the respiratory system)	Men	18.3	10	0.0504
Mexico City	Álvaro Obregón	Chapter 10 (Diseases of the respiratory system)	Women	17.5	10	0.0647
Mexico City	Álvaro Obregón	Chapter 5 (Mental and behavioural disorders)	Men	17.3	10	0.0674
Mexico City	Álvaro Obregón	Chapter 5 (Mental and behavioural disorders)	Women	10.4	10	0.4057
Mexico City	Tláhuac	Chapter 9 (Diseases of the circulatory system)	Men	7.6	10	0.6673
Mexico City	Tláhuac	Chapter 9 (Diseases of the circulatory system)	Women	14.7	10	0.1422
Mexico City	Tláhuac	Chapter 4 (Endocrine, nutritional and metabolic diseases)	Men	16.7	10	0.0820
Mexico City	Tláhuac	Chapter 4 (Endocrine, nutritional and metabolic diseases)	Women	13.0	10	0.2260
Mexico City	Tláhuac	Chapter 6 (Diseases of the nervous system)	Men	8.1	10	0.6172
Mexico City	Tláhuac	Chapter 6 (Diseases of the nervous system)	Women	16.9	10	0.0767
Mexico City	Tláhuac	Chapter 10 (Diseases of the respiratory system)	Men	11.7	10	0.3057
Mexico City	Tláhuac	Chapter 10 (Diseases of the respiratory system)	Women	6.0	10	0.8130
Mexico City	Tláhuac	Chapter 5 (Mental and behavioural disorders)	Men	29.2	10	**0.0012***
Mexico City	Tláhuac	Chapter 5 (Mental and behavioural disorders)	Women	8.1	10	0.6197
Mexico City	Tlalpan	Chapter 9 (Diseases of the circulatory system)	Men	13.3	10	0.2055
Mexico City	Tlalpan	Chapter 9 (Diseases of the circulatory system)	Women	20.1	10	**0.0280***
Mexico City	Tlalpan	Chapter 4 (Endocrine, nutritional and metabolic diseases)	Men	13.5	10	0.1967
Mexico City	Tlalpan	Chapter 4 (Endocrine, nutritional and metabolic diseases)	Women	8.3	10	0.5986
Mexico City	Tlalpan	Chapter 6 (Diseases of the nervous system)	Men	9.6	10	0.4766
Mexico City	Tlalpan	Chapter 6 (Diseases of the nervous system)	Women	6.4	10	0.7764
Mexico City	Tlalpan	Chapter 10 (Diseases of the respiratory system)	Men	9.8	10	0.4624
Mexico City	Tlalpan	Chapter 10 (Diseases of the respiratory system)	Women	10.7	10	0.3838
Mexico City	Tlalpan	Chapter 5 (Mental and behavioural disorders)	Men	8.4	10	0.5855
Mexico City	Tlalpan	Chapter 5 (Mental and behavioural disorders)	Women	7.4	10	0.6859
Mexico City	Xochimilco	Chapter 9 (Diseases of the circulatory system)	Men	13.8	10	0.1815
Mexico City	Xochimilco	Chapter 9 (Diseases of the circulatory system)	Women	8.8	10	0.5501
Mexico City	Xochimilco	Chapter 4 (Endocrine, nutritional and metabolic diseases)	Men	9.8	10	0.4545
Mexico City	Xochimilco	Chapter 4 (Endocrine, nutritional and metabolic diseases)	Women	9.5	10	0.4881
Mexico City	Xochimilco	Chapter 6 (Diseases of the nervous system)	Men	19.9	10	**0.0305***
Mexico City	Xochimilco	Chapter 6 (Diseases of the nervous system)	Women	8.7	10	0.5645
Mexico City	Xochimilco	Chapter 10 (Diseases of the respiratory system)	Men	16.6	10	0.0829
Mexico City	Xochimilco	Chapter 10 (Diseases of the respiratory system)	Women	6.7	10	0.7551
Mexico City	Xochimilco	Chapter 5 (Mental and behavioural disorders)	Men	15.1	10	0.1284
Mexico City	Xochimilco	Chapter 5 (Mental and behavioural disorders)	Women	7.1	10	0.7165
Mexico City	Benito Juárez	Chapter 9 (Diseases of the circulatory system)	Men	10.7	10	0.3832
Mexico City	Benito Juárez	Chapter 9 (Diseases of the circulatory system)	Women	5.7	10	0.8414
Mexico City	Benito Juárez	Chapter 4 (Endocrine, nutritional and metabolic diseases)	Men	11.9	10	0.2925
Mexico City	Benito Juárez	Chapter 4 (Endocrine, nutritional and metabolic diseases)	Women	10.6	10	0.3919
Mexico City	Benito Juárez	Chapter 6 (Diseases of the nervous system)	Men	7.4	10	0.6838
Mexico City	Benito Juárez	Chapter 6 (Diseases of the nervous system)	Women	12.8	10	0.2323

Mexico City	Benito Juárez	Chapter 10 (Diseases of the respiratory system)	Men	7.3	10	0.6960
Mexico City	Benito Juárez	Chapter 10 (Diseases of the respiratory system)	Women	8.6	10	0.5735
Mexico City	Benito Juárez	Chapter 5 (Mental and behavioural disorders)	Men	10.3	10	0.4186
Mexico City	Benito Juárez	Chapter 5 (Mental and behavioural disorders)	Women	10.9	10	0.3668
Mexico City	Cuauhtémoc	Chapter 9 (Diseases of the circulatory system)	Men	9.5	10	0.4864
Mexico City	Cuauhtémoc	Chapter 9 (Diseases of the circulatory system)	Women	4.5	10	0.9237
Mexico City	Cuauhtémoc	Chapter 4 (Endocrine, nutritional and metabolic diseases)	Men	13.2	10	0.2106
Mexico City	Cuauhtémoc	Chapter 4 (Endocrine, nutritional and metabolic diseases)	Women	17.6	10	0.0629
Mexico City	Cuauhtémoc	Chapter 6 (Diseases of the nervous system)	Men	8.8	10	0.5476
Mexico City	Cuauhtémoc	Chapter 6 (Diseases of the nervous system)	Women	16.9	10	0.0764
Mexico City	Cuauhtémoc	Chapter 10 (Diseases of the respiratory system)	Men	6.7	10	0.7503
Mexico City	Cuauhtémoc	Chapter 10 (Diseases of the respiratory system)	Women	17.1	10	0.0729
Mexico City	Cuauhtémoc	Chapter 5 (Mental and behavioural disorders)	Men	8.6	10	0.5749
Mexico City	Cuauhtémoc	Chapter 5 (Mental and behavioural disorders)	Women	21.0	10	**0.0210***
Mexico City	Miguel Hidalgo	Chapter 9 (Diseases of the circulatory system)	Men	14.6	10	0.1459
Mexico City	Miguel Hidalgo	Chapter 9 (Diseases of the circulatory system)	Women	16.1	10	0.0977
Mexico City	Miguel Hidalgo	Chapter 4 (Endocrine, nutritional and metabolic diseases)	Men	9.7	10	0.4635
Mexico City	Miguel Hidalgo	Chapter 4 (Endocrine, nutritional and metabolic diseases)	Women	10.7	10	0.3797
Mexico City	Miguel Hidalgo	Chapter 6 (Diseases of the nervous system)	Men	9.4	10	0.4942
Mexico City	Miguel Hidalgo	Chapter 6 (Diseases of the nervous system)	Women	14.4	10	0.1561
Mexico City	Miguel Hidalgo	Chapter 10 (Diseases of the respiratory system)	Men	7.1	10	0.7130
Mexico City	Miguel Hidalgo	Chapter 10 (Diseases of the respiratory system)	Women	6.1	10	0.8078
Mexico City	Miguel Hidalgo	Chapter 5 (Mental and behavioural disorders)	Men	15.8	10	0.1059
Mexico City	Miguel Hidalgo	Chapter 5 (Mental and behavioural disorders)	Women	16.9	10	0.0772
Mexico City	Venustiano Carranza	Chapter 9 (Diseases of the circulatory system)	Men	8.4	10	0.5905
Mexico City	Venustiano Carranza	Chapter 9 (Diseases of the circulatory system)	Women	16.5	10	0.0854
Mexico City	Venustiano Carranza	Chapter 4 (Endocrine, nutritional and metabolic diseases)	Men	11.7	10	0.3077
Mexico City	Venustiano Carranza	Chapter 4 (Endocrine, nutritional and metabolic diseases)	Women	13.2	10	0.2140
Mexico City	Venustiano Carranza	Chapter 6 (Diseases of the nervous system)	Men	10.0	10	0.4406
Mexico City	Venustiano Carranza	Chapter 6 (Diseases of the nervous system)	Women	11.2	10	0.3437
Mexico City	Venustiano Carranza	Chapter 10 (Diseases of the respiratory system)	Men	7.3	10	0.6990
Mexico City	Venustiano Carranza	Chapter 10 (Diseases of the respiratory system)	Women	4.8	10	0.9065
Mexico City	Venustiano Carranza	Chapter 5 (Mental and behavioural disorders)	Men	14.5	10	0.1499
Mexico City	Venustiano Carranza	Chapter 5 (Mental and behavioural disorders)	Women	13.1	10	0.2170
Hidalgo	Tizayuca	Chapter 9 (Diseases of the circulatory system)	Men	4.6	10	0.9139
Hidalgo	Tizayuca	Chapter 9 (Diseases of the circulatory system)	Women	5.4	10	0.8651
Hidalgo	Tizayuca	Chapter 4 (Endocrine, nutritional and metabolic diseases)	Men	8.1	10	0.6201
Hidalgo	Tizayuca	Chapter 4 (Endocrine, nutritional and metabolic diseases)	Women	16.0	10	0.0995
Hidalgo	Tizayuca	Chapter 6 (Diseases of the nervous system)	Men	16.6	10	0.0828
Hidalgo	Tizayuca	Chapter 6 (Diseases of the nervous system)	Women	19.1	10	**0.0385***
Hidalgo	Tizayuca	Chapter 10 (Diseases of the respiratory system)	Men	6.4	10	0.7782
Hidalgo	Tizayuca	Chapter 10 (Diseases of the respiratory system)	Women	7.1	10	0.7142
Hidalgo	Tizayuca	Chapter 5 (Mental and behavioural disorders)	Men	6.5	10	0.7702

Hidalgo	Tizayuca	Chapter 5 (Mental and behavioural disorders)	Women	2.5	4	0.6372
State of Mexico	Acolman	Chapter 9 (Diseases of the circulatory system)	Men	14.5	10	0.1496
State of Mexico	Acolman	Chapter 9 (Diseases of the circulatory system)	Women	22.2	10	**0.0141***
State of Mexico	Acolman	Chapter 4 (Endocrine, nutritional and metabolic diseases)	Men	5.2	10	0.8743
State of Mexico	Acolman	Chapter 4 (Endocrine, nutritional and metabolic diseases)	Women	8.9	10	0.5372
State of Mexico	Acolman	Chapter 6 (Diseases of the nervous system)	Men	9.8	10	0.4570
State of Mexico	Acolman	Chapter 6 (Diseases of the nervous system)	Women	6.7	10	0.7556
State of Mexico	Acolman	Chapter 10 (Diseases of the respiratory system)	Men	20.2	10	**0.0275***
State of Mexico	Acolman	Chapter 10 (Diseases of the respiratory system)	Women	7.5	10	0.6806
State of Mexico	Acolman	Chapter 5 (Mental and behavioural disorders)	Men	5.6	10	0.8504
State of Mexico	Acolman	Chapter 5 (Mental and behavioural disorders)	Women	8.9	9	0.4488
State of Mexico	Amecameca	Chapter 9 (Diseases of the circulatory system)	Men	8.8	10	0.5527
State of Mexico	Amecameca	Chapter 9 (Diseases of the circulatory system)	Women	7.9	10	0.6387
State of Mexico	Amecameca	Chapter 4 (Endocrine, nutritional and metabolic diseases)	Men	5.2	10	0.8777
State of Mexico	Amecameca	Chapter 4 (Endocrine, nutritional and metabolic diseases)	Women	9.6	10	0.4775
State of Mexico	Amecameca	Chapter 6 (Diseases of the nervous system)	Men	4.1	10	0.9419
State of Mexico	Amecameca	Chapter 6 (Diseases of the nervous system)	Women	14.0	10	0.1733
State of Mexico	Amecameca	Chapter 10 (Diseases of the respiratory system)	Men	11.6	10	0.3159
State of Mexico	Amecameca	Chapter 10 (Diseases of the respiratory system)	Women	10.3	10	0.4152
State of Mexico	Amecameca	Chapter 5 (Mental and behavioural disorders)	Men	8.8	10	0.5506
State of Mexico	Amecameca	Chapter 5 (Mental and behavioural disorders)	Women	4.0	6	0.6786
State of Mexico	Apaxco	Chapter 9 (Diseases of the circulatory system)	Men	14.3	10	0.1603
State of Mexico	Apaxco	Chapter 9 (Diseases of the circulatory system)	Women	8.6	10	0.5727
State of Mexico	Apaxco	Chapter 4 (Endocrine, nutritional and metabolic diseases)	Men	12.5	10	0.2532
State of Mexico	Apaxco	Chapter 4 (Endocrine, nutritional and metabolic diseases)	Women	11.6	10	0.3124
State of Mexico	Apaxco	Chapter 6 (Diseases of the nervous system)	Women	6.8	10	0.7439
State of Mexico	Apaxco	Chapter 6 (Diseases of the nervous system)	Men	10.6	10	0.3873
State of Mexico	Apaxco	Chapter 10 (Diseases of the respiratory system)	Men	9.3	10	0.5073
State of Mexico	Apaxco	Chapter 10 (Diseases of the respiratory system)	Women	9.2	10	0.5093
State of Mexico	Apaxco	Chapter 5 (Mental and behavioural disorders)	Men	12.1	10	0.2804
State of Mexico	Atenco	Chapter 9 (Diseases of the circulatory system)	Men	19.8	10	**0.0316***
State of Mexico	Atenco	Chapter 9 (Diseases of the circulatory system)	Women	9.4	10	0.4939
State of Mexico	Atenco	Chapter 4 (Endocrine, nutritional and metabolic diseases)	Men	5.0	10	0.8881
State of Mexico	Atenco	Chapter 4 (Endocrine, nutritional and metabolic diseases)	Women	8.7	10	0.5627
State of Mexico	Atenco	Chapter 6 (Diseases of the nervous system)	Men	20.0	10	**0.0290***
State of Mexico	Atenco	Chapter 6 (Diseases of the nervous system)	Women	12.9	10	0.2308
State of Mexico	Atenco	Chapter 10 (Diseases of the respiratory system)	Men	19.6	10	**0.0333***
State of Mexico	Atenco	Chapter 10 (Diseases of the respiratory system)	Women	15.6	10	0.1105
State of Mexico	Atenco	Chapter 5 (Mental and behavioural disorders)	Men	11.6	8	0.1690
State of Mexico	Atizapán de Zaragoza	Chapter 9 (Diseases of the circulatory system)	Men	13.4	10	0.2007
State of Mexico	Atizapán de Zaragoza	Chapter 9 (Diseases of the circulatory system)	Women	11.9	10	0.2931
State of Mexico	Atizapán de Zaragoza	Chapter 4 (Endocrine, nutritional and metabolic diseases)	Men	26.3	10	**0.0033***
State of Mexico	Atizapán de Zaragoza	Chapter 4 (Endocrine, nutritional and metabolic diseases)	Women	9.7	10	0.4683

State of Mexico	Atizapán de Zaragoza	Chapter 6 (Diseases of the nervous system)	Men	15.3	10	0.1204
State of Mexico	Atizapán de Zaragoza	Chapter 6 (Diseases of the nervous system)	Women	7.4	10	0.6892
State of Mexico	Atizapán de Zaragoza	Chapter 10 (Diseases of the respiratory system)	Men	17.7	10	0.0610
State of Mexico	Atizapán de Zaragoza	Chapter 10 (Diseases of the respiratory system)	Women	5.0	10	0.8937
State of Mexico	Atizapán de Zaragoza	Chapter 5 (Mental and behavioural disorders)	Men	9.0	10	0.5333
State of Mexico	Atizapán de Zaragoza	Chapter 5 (Mental and behavioural disorders)	Women	12.6	10	0.2446
State of Mexico	Atlautla	Chapter 9 (Diseases of the circulatory system)	Men	6.6	10	0.7601
State of Mexico	Atlautla	Chapter 9 (Diseases of the circulatory system)	Women	10.2	10	0.4223
State of Mexico	Atlautla	Chapter 4 (Endocrine, nutritional and metabolic diseases)	Men	30.2	10	**0.0008***
State of Mexico	Atlautla	Chapter 4 (Endocrine, nutritional and metabolic diseases)	Women	6.2	10	0.7974
State of Mexico	Atlautla	Chapter 6 (Diseases of the nervous system)	Men	23.1	10	**0.0105***
State of Mexico	Atlautla	Chapter 6 (Diseases of the nervous system)	Women	6.7	5	0.2430
State of Mexico	Atlautla	Chapter 10 (Diseases of the respiratory system)	Men	11.6	10	0.3123
State of Mexico	Atlautla	Chapter 10 (Diseases of the respiratory system)	Women	25.6	10	**0.0043***
State of Mexico	Atlautla	Chapter 5 (Mental and behavioural disorders)	Men	4.9	7	0.6682
State of Mexico	Axapusco	Chapter 9 (Diseases of the circulatory system)	Men	16.6	10	0.0849
State of Mexico	Axapusco	Chapter 9 (Diseases of the circulatory system)	Women	10.5	10	0.3997
State of Mexico	Axapusco	Chapter 4 (Endocrine, nutritional and metabolic diseases)	Men	9.3	10	0.5020
State of Mexico	Axapusco	Chapter 4 (Endocrine, nutritional and metabolic diseases)	Women	14.6	10	0.1484
State of Mexico	Axapusco	Chapter 6 (Diseases of the nervous system)	Men	8.0	9	0.5392
State of Mexico	Axapusco	Chapter 6 (Diseases of the nervous system)	Women	8.6	9	0.4703
State of Mexico	Axapusco	Chapter 10 (Diseases of the respiratory system)	Men	6.5	10	0.7685
State of Mexico	Axapusco	Chapter 10 (Diseases of the respiratory system)	Women	8.3	10	0.6010
State of Mexico	Axapusco	Chapter 5 (Mental and behavioural disorders)	Men	10.4	10	0.4098
State of Mexico	Ayapango	Chapter 9 (Diseases of the circulatory system)	Women	11.1	10	0.3507
State of Mexico	Ayapango	Chapter 9 (Diseases of the circulatory system)	Men	13.0	10	0.2244
State of Mexico	Ayapango	Chapter 4 (Endocrine, nutritional and metabolic diseases)	Men	11.1	10	0.3531
State of Mexico	Ayapango	Chapter 4 (Endocrine, nutritional and metabolic diseases)	Women	11.6	10	0.3127
State of Mexico	Ayapango	Chapter 6 (Diseases of the nervous system)	Men	13.1	8	0.1093
State of Mexico	Ayapango	Chapter 6 (Diseases of the nervous system)	Women	1.9	5	0.8576
State of Mexico	Ayapango	Chapter 10 (Diseases of the respiratory system)	Men	8.4	10	0.5857
State of Mexico	Ayapango	Chapter 10 (Diseases of the respiratory system)	Women	7.8	10	0.6499
State of Mexico	Coacalco de Berriozábal	Chapter 9 (Diseases of the circulatory system)	Men	13.6	10	0.1908
State of Mexico	Coacalco de Berriozábal	Chapter 9 (Diseases of the circulatory system)	Women	3.4	10	0.9714
State of Mexico	Coacalco de Berriozábal	Chapter 4 (Endocrine, nutritional and metabolic diseases)	Men	3.9	10	0.9512
State of Mexico	Coacalco de Berriozábal	Chapter 4 (Endocrine, nutritional and metabolic diseases)	Women	6.1	10	0.8089
State of Mexico	Coacalco de Berriozábal	Chapter 6 (Diseases of the nervous system)	Men	12.1	10	0.2810
State of Mexico	Coacalco de Berriozábal	Chapter 6 (Diseases of the nervous system)	Women	9.8	10	0.4625
State of Mexico	Coacalco de Berriozábal	Chapter 10 (Diseases of the respiratory system)	Men	5.8	10	0.8302
State of Mexico	Coacalco de Berriozábal	Chapter 10 (Diseases of the respiratory system)	Women	16.5	10	0.0851
State of Mexico	Coacalco de Berriozábal	Chapter 5 (Mental and behavioural disorders)	Men	3.8	10	0.9541
State of Mexico	Coacalco de Berriozábal	Chapter 5 (Mental and behavioural disorders)	Women	5.9	10	0.8216
State of Mexico	Cocotitlán	Chapter 9 (Diseases of the circulatory system)	Men	11.9	10	0.2897

State of Mexico	Cocotitlán	Chapter 9 (Diseases of the circulatory system)	Women	8.9	10	0.5419
State of Mexico	Cocotitlán	Chapter 4 (Endocrine, nutritional and metabolic diseases)	Men	7.8	10	0.6488
State of Mexico	Cocotitlán	Chapter 4 (Endocrine, nutritional and metabolic diseases)	Women	8.9	10	0.5424
State of Mexico	Cocotitlán	Chapter 6 (Diseases of the nervous system)	Men	2.2	4	0.6977
State of Mexico	Cocotitlán	Chapter 6 (Diseases of the nervous system)	Women	5.0	7	0.6616
State of Mexico	Cocotitlán	Chapter 10 (Diseases of the respiratory system)	Men	6.6	10	0.7589
State of Mexico	Cocotitlán	Chapter 10 (Diseases of the respiratory system)	Women	10.9	10	0.3652
State of Mexico	Coyotepec	Chapter 9 (Diseases of the circulatory system)	Men	5.8	10	0.8299
State of Mexico	Coyotepec	Chapter 9 (Diseases of the circulatory system)	Women	32.2	10	**0.0004***
State of Mexico	Coyotepec	Chapter 4 (Endocrine, nutritional and metabolic diseases)	Men	14.6	10	0.1460
State of Mexico	Coyotepec	Chapter 4 (Endocrine, nutritional and metabolic diseases)	Women	24.4	10	**0.0067***
State of Mexico	Coyotepec	Chapter 6 (Diseases of the nervous system)	Men	13.4	10	0.2030
State of Mexico	Coyotepec	Chapter 6 (Diseases of the nervous system)	Women	4.1	4	0.3880
State of Mexico	Coyotepec	Chapter 10 (Diseases of the respiratory system)	Men	8.1	10	0.6231
State of Mexico	Coyotepec	Chapter 10 (Diseases of the respiratory system)	Women	9.9	10	0.4484
State of Mexico	Coyotepec	Chapter 5 (Mental and behavioural disorders)	Men	7.6	5	0.1817
State of Mexico	Cuautitlán	Chapter 9 (Diseases of the circulatory system)	Men	13.3	10	0.2072
State of Mexico	Cuautitlán	Chapter 9 (Diseases of the circulatory system)	Women	16.3	10	0.0920
State of Mexico	Cuautitlán	Chapter 4 (Endocrine, nutritional and metabolic diseases)	Men	9.9	10	0.4537
State of Mexico	Cuautitlán	Chapter 4 (Endocrine, nutritional and metabolic diseases)	Women	7.9	10	0.6410
State of Mexico	Cuautitlán	Chapter 6 (Diseases of the nervous system)	Men	9.8	10	0.4560
State of Mexico	Cuautitlán	Chapter 6 (Diseases of the nervous system)	Women	6.4	10	0.7764
State of Mexico	Cuautitlán	Chapter 10 (Diseases of the respiratory system)	Men	23.3	10	**0.0098***
State of Mexico	Cuautitlán	Chapter 10 (Diseases of the respiratory system)	Women	30.3	10	**0.0008***
State of Mexico	Cuautitlán	Chapter 5 (Mental and behavioural disorders)	Men	7.5	10	0.6757
State of Mexico	Cuautitlán	Chapter 5 (Mental and behavioural disorders)	Women	7.1	5	0.2102
State of Mexico	Chalco	Chapter 9 (Diseases of the circulatory system)	Men	10.2	10	0.4225
State of Mexico	Chalco	Chapter 9 (Diseases of the circulatory system)	Women	9.7	10	0.4639
State of Mexico	Chalco	Chapter 4 (Endocrine, nutritional and metabolic diseases)	Men	13.3	10	0.2097
State of Mexico	Chalco	Chapter 4 (Endocrine, nutritional and metabolic diseases)	Women	13.5	10	0.1946
State of Mexico	Chalco	Chapter 6 (Diseases of the nervous system)	Men	10.0	10	0.4385
State of Mexico	Chalco	Chapter 6 (Diseases of the nervous system)	Women	7.9	10	0.6351
State of Mexico	Chalco	Chapter 10 (Diseases of the respiratory system)	Men	12.7	10	0.2431
State of Mexico	Chalco	Chapter 10 (Diseases of the respiratory system)	Women	6.9	10	0.7373
State of Mexico	Chalco	Chapter 5 (Mental and behavioural disorders)	Men	14.9	10	0.1347
State of Mexico	Chalco	Chapter 5 (Mental and behavioural disorders)	Women	20.2	9	**0.0167***
State of Mexico	Chiautla	Chapter 9 (Diseases of the circulatory system)	Men	20.9	10	**0.0221***
State of Mexico	Chiautla	Chapter 9 (Diseases of the circulatory system)	Women	12.1	10	0.2803
State of Mexico	Chiautla	Chapter 4 (Endocrine, nutritional and metabolic diseases)	Men	8.5	10	0.5776
State of Mexico	Chiautla	Chapter 4 (Endocrine, nutritional and metabolic diseases)	Women	9.8	10	0.4570
State of Mexico	Chiautla	Chapter 6 (Diseases of the nervous system)	Men	5.1	7	0.6478
State of Mexico	Chiautla	Chapter 6 (Diseases of the nervous system)	Women	7.7	9	0.5656
State of Mexico	Chiautla	Chapter 10 (Diseases of the respiratory system)	Men	6.2	10	0.8002

State of Mexico	Chiautla	Chapter 10 (Diseases of the respiratory system)	Women	13.9	10	0.1765
State of Mexico	Chiautla	Chapter 5 (Mental and behavioural disorders)	Men	4.4	8	0.8170
State of Mexico	Chicoloapan	Chapter 9 (Diseases of the circulatory system)	Men	16.4	10	0.0886
State of Mexico	Chicoloapan	Chapter 9 (Diseases of the circulatory system)	Women	4.6	10	0.9137
State of Mexico	Chicoloapan	Chapter 4 (Endocrine, nutritional and metabolic diseases)	Men	8.4	10	0.5904
State of Mexico	Chicoloapan	Chapter 4 (Endocrine, nutritional and metabolic diseases)	Women	15.0	10	0.1332
State of Mexico	Chicoloapan	Chapter 6 (Diseases of the nervous system)	Men	13.3	10	0.2067
State of Mexico	Chicoloapan	Chapter 6 (Diseases of the nervous system)	Women	14.3	10	0.1582
State of Mexico	Chicoloapan	Chapter 10 (Diseases of the respiratory system)	Men	6.5	10	0.7739
State of Mexico	Chicoloapan	Chapter 10 (Diseases of the respiratory system)	Women	15.2	10	0.1234
State of Mexico	Chicoloapan	Chapter 5 (Mental and behavioural disorders)	Men	9.7	10	0.4707
State of Mexico	Chicoloapan	Chapter 5 (Mental and behavioural disorders)	Women	3.2	6	0.7881
State of Mexico	Chiconcuac	Chapter 9 (Diseases of the circulatory system)	Men	10.8	10	0.3747
State of Mexico	Chiconcuac	Chapter 9 (Diseases of the circulatory system)	Women	26.4	10	**0.0032***
State of Mexico	Chiconcuac	Chapter 4 (Endocrine, nutritional and metabolic diseases)	Men	13.7	10	0.1870
State of Mexico	Chiconcuac	Chapter 4 (Endocrine, nutritional and metabolic diseases)	Women	10.9	10	0.3633
State of Mexico	Chiconcuac	Chapter 6 (Diseases of the nervous system)	Men	13.6	9	0.1382
State of Mexico	Chiconcuac	Chapter 6 (Diseases of the nervous system)	Women	8.5	10	0.5829
State of Mexico	Chiconcuac	Chapter 10 (Diseases of the respiratory system)	Men	11.3	10	0.3383
State of Mexico	Chiconcuac	Chapter 10 (Diseases of the respiratory system)	Women	15.0	10	0.1311
State of Mexico	Chiconcuac	Chapter 5 (Mental and behavioural disorders)	Men	8.9	7	0.2623
State of Mexico	Chimalhuacán	Chapter 9 (Diseases of the circulatory system)	Men	11.4	10	0.3288
State of Mexico	Chimalhuacán	Chapter 9 (Diseases of the circulatory system)	Women	9.9	10	0.4490
State of Mexico	Chimalhuacán	Chapter 4 (Endocrine, nutritional and metabolic diseases)	Men	6.9	10	0.7312
State of Mexico	Chimalhuacán	Chapter 4 (Endocrine, nutritional and metabolic diseases)	Women	8.3	10	0.5975
State of Mexico	Chimalhuacán	Chapter 6 (Diseases of the nervous system)	Men	7.3	10	0.6994
State of Mexico	Chimalhuacán	Chapter 6 (Diseases of the nervous system)	Women	13.3	10	0.2099
State of Mexico	Chimalhuacán	Chapter 10 (Diseases of the respiratory system)	Men	4.7	10	0.9105
State of Mexico	Chimalhuacán	Chapter 10 (Diseases of the respiratory system)	Women	12.7	10	0.2391
State of Mexico	Chimalhuacán	Chapter 5 (Mental and behavioural disorders)	Men	19.6	10	**0.0338***
State of Mexico	Chimalhuacán	Chapter 5 (Mental and behavioural disorders)	Women	10.1	10	0.4330
State of Mexico	Ecatepec de Morelos	Chapter 9 (Diseases of the circulatory system)	Men	14.4	10	0.1535
State of Mexico	Ecatepec de Morelos	Chapter 9 (Diseases of the circulatory system)	Women	9.8	10	0.4593
State of Mexico	Ecatepec de Morelos	Chapter 4 (Endocrine, nutritional and metabolic diseases)	Men	4.2	10	0.9364
State of Mexico	Ecatepec de Morelos	Chapter 4 (Endocrine, nutritional and metabolic diseases)	Women	4.4	10	0.9249
State of Mexico	Ecatepec de Morelos	Chapter 6 (Diseases of the nervous system)	Men	7.0	10	0.7239
State of Mexico	Ecatepec de Morelos	Chapter 6 (Diseases of the nervous system)	Women	13.3	10	0.2053
State of Mexico	Ecatepec de Morelos	Chapter 10 (Diseases of the respiratory system)	Men	4.7	10	0.9100
State of Mexico	Ecatepec de Morelos	Chapter 10 (Diseases of the respiratory system)	Women	3.6	10	0.9620
State of Mexico	Ecatepec de Morelos	Chapter 5 (Mental and behavioural disorders)	Men	10.2	10	0.4191
State of Mexico	Ecatepec de Morelos	Chapter 5 (Mental and behavioural disorders)	Women	10.8	10	0.3728
State of Mexico	Ecatzingo	Chapter 9 (Diseases of the circulatory system)	Men	10.0	10	0.4406
State of Mexico	Ecatzingo	Chapter 9 (Diseases of the circulatory system)	Women	4.9	10	0.8979

State of Mexico	Ecatzingo	Chapter 4 (Endocrine, nutritional and metabolic diseases)	Men		6.1	10	0.8103
State of Mexico	Ecatzingo	Chapter 4 (Endocrine, nutritional and metabolic diseases)	Women		8.3	10	0.6017
State of Mexico	Ecatzingo	Chapter 10 (Diseases of the respiratory system)	Men		25.6	10	**0.0043***
State of Mexico	Ecatzingo	Chapter 10 (Diseases of the respiratory system)	Women		8.5	10	0.5762
State of Mexico	Huehuetoca	Chapter 9 (Diseases of the circulatory system)	Men		15.1	10	0.1290
State of Mexico	Huehuetoca	Chapter 9 (Diseases of the circulatory system)	Women		15.7	10	0.1092
State of Mexico	Huehuetoca	Chapter 4 (Endocrine, nutritional and metabolic diseases)	Men		23.1	10	**0.0105***
State of Mexico	Huehuetoca	Chapter 4 (Endocrine, nutritional and metabolic diseases)	Women		9.2	10	0.5121
State of Mexico	Huehuetoca	Chapter 6 (Diseases of the nervous system)	Women		16.6	10	0.0833
State of Mexico	Huehuetoca	Chapter 6 (Diseases of the nervous system)	Men		18.9	10	**0.0411***
State of Mexico	Huehuetoca	Chapter 10 (Diseases of the respiratory system)	Men		6.3	10	0.7858
State of Mexico	Huehuetoca	Chapter 10 (Diseases of the respiratory system)	Women		8.9	10	0.5406
State of Mexico	Huehuetoca	Chapter 5 (Mental and behavioural disorders)	Men		9.5	7	0.2167
State of Mexico	Huehuetoca	Chapter 5 (Mental and behavioural disorders)	Women		2.2	4	0.6954
State of Mexico	Hueypoxtla	Chapter 9 (Diseases of the circulatory system)	Men		9.2	10	0.5086
State of Mexico	Hueypoxtla	Chapter 9 (Diseases of the circulatory system)	Women		10.7	10	0.3779
State of Mexico	Hueypoxtla	Chapter 4 (Endocrine, nutritional and metabolic diseases)	Men		9.2	10	0.5146
State of Mexico	Hueypoxtla	Chapter 4 (Endocrine, nutritional and metabolic diseases)	Women		9.5	10	0.4887
State of Mexico	Hueypoxtla	Chapter 6 (Diseases of the nervous system)	Men		13.6	10	0.1917
State of Mexico	Hueypoxtla	Chapter 6 (Diseases of the nervous system)	Women		9.2	10	0.5170
State of Mexico	Hueypoxtla	Chapter 10 (Diseases of the respiratory system)	Men		17.5	10	0.0638
State of Mexico	Hueypoxtla	Chapter 10 (Diseases of the respiratory system)	Women		14.0	10	0.1752
State of Mexico	Hueypoxtla	Chapter 5 (Mental and behavioural disorders)	Men		11.6	10	0.3119
State of Mexico	Huixquilucan	Chapter 9 (Diseases of the circulatory system)	Men		15.1	10	0.1269
State of Mexico	Huixquilucan	Chapter 9 (Diseases of the circulatory system)	Women		53.5	10	**0.0000***
State of Mexico	Huixquilucan	Chapter 4 (Endocrine, nutritional and metabolic diseases)	Men		37.5	10	**0.0000***
State of Mexico	Huixquilucan	Chapter 4 (Endocrine, nutritional and metabolic diseases)	Women		18.2	10	0.0515
State of Mexico	Huixquilucan	Chapter 6 (Diseases of the nervous system)	Men		10.1	10	0.4292
State of Mexico	Huixquilucan	Chapter 6 (Diseases of the nervous system)	Women		7.2	10	0.7103
State of Mexico	Huixquilucan	Chapter 10 (Diseases of the respiratory system)	Men		8.6	10	0.5656
State of Mexico	Huixquilucan	Chapter 10 (Diseases of the respiratory system)	Women		14.9	10	0.1356
State of Mexico	Huixquilucan	Chapter 5 (Mental and behavioural disorders)	Men		21.6	10	**0.0175***
State of Mexico	Huixquilucan	Chapter 5 (Mental and behavioural disorders)	Women		6.8	7	0.4545
State of Mexico	Isidro Fabela	Chapter 9 (Diseases of the circulatory system)	Men		4.5	10	0.9196
State of Mexico	Isidro Fabela	Chapter 9 (Diseases of the circulatory system)	Women		7.3	10	0.6986
State of Mexico	Isidro Fabela	Chapter 4 (Endocrine, nutritional and metabolic diseases)	Men		10.7	10	0.3777
State of Mexico	Isidro Fabela	Chapter 4 (Endocrine, nutritional and metabolic diseases)	Women		6.9	10	0.7352
State of Mexico	Isidro Fabela	Chapter 6 (Diseases of the nervous system)	Men		9.5	5	0.0898
State of Mexico	Isidro Fabela	Chapter 10 (Diseases of the respiratory system)	Men		4.1	10	0.9428
State of Mexico	Isidro Fabela	Chapter 10 (Diseases of the respiratory system)	Women		6.8	10	0.7449
State of Mexico	Ixtapaluca	Chapter 9 (Diseases of the circulatory system)	Men		19.0	10	**0.0398***
State of Mexico	Ixtapaluca	Chapter 9 (Diseases of the circulatory system)	Women		20.8	10	**0.0226***
State of Mexico	Ixtapaluca	Chapter 4 (Endocrine, nutritional and metabolic diseases)	Men		3.5	10	0.9680

State of Mexico	Ixtapaluca	Chapter 4 (Endocrine, nutritional and metabolic diseases)	Women	7.3	10	0.6946
State of Mexico	Ixtapaluca	Chapter 6 (Diseases of the nervous system)	Men	11.3	10	0.3357
State of Mexico	Ixtapaluca	Chapter 6 (Diseases of the nervous system)	Women	8.4	10	0.5906
State of Mexico	Ixtapaluca	Chapter 10 (Diseases of the respiratory system)	Men	4.8	10	0.9072
State of Mexico	Ixtapaluca	Chapter 10 (Diseases of the respiratory system)	Women	19.9	10	**0.0307***
State of Mexico	Ixtapaluca	Chapter 5 (Mental and behavioural disorders)	Men	9.3	10	0.5061
State of Mexico	Ixtapaluca	Chapter 5 (Mental and behavioural disorders)	Women	12.1	10	0.2783
State of Mexico	Jaltenco	Chapter 9 (Diseases of the circulatory system)	Men	17.1	10	0.0717
State of Mexico	Jaltenco	Chapter 9 (Diseases of the circulatory system)	Women	7.6	10	0.6643
State of Mexico	Jaltenco	Chapter 4 (Endocrine, nutritional and metabolic diseases)	Men	15.3	10	0.1226
State of Mexico	Jaltenco	Chapter 4 (Endocrine, nutritional and metabolic diseases)	Women	16.1	10	0.0959
State of Mexico	Jaltenco	Chapter 6 (Diseases of the nervous system)	Women	6.4	8	0.6011
State of Mexico	Jaltenco	Chapter 6 (Diseases of the nervous system)	Men	4.0	6	0.6817
State of Mexico	Jaltenco	Chapter 10 (Diseases of the respiratory system)	Men	11.9	10	0.2944
State of Mexico	Jaltenco	Chapter 10 (Diseases of the respiratory system)	Women	6.3	10	0.7900
State of Mexico	Jilotzingo	Chapter 9 (Diseases of the circulatory system)	Men	6.2	10	0.8007
State of Mexico	Jilotzingo	Chapter 9 (Diseases of the circulatory system)	Women	3.5	10	0.9665
State of Mexico	Jilotzingo	Chapter 4 (Endocrine, nutritional and metabolic diseases)	Men	14.2	10	0.1642
State of Mexico	Jilotzingo	Chapter 4 (Endocrine, nutritional and metabolic diseases)	Women	12.4	10	0.2607
State of Mexico	Jilotzingo	Chapter 6 (Diseases of the nervous system)	Men	5.5	5	0.3540
State of Mexico	Jilotzingo	Chapter 6 (Diseases of the nervous system)	Women	3.9	7	0.7889
State of Mexico	Jilotzingo	Chapter 10 (Diseases of the respiratory system)	Men	11.8	10	0.3003
State of Mexico	Jilotzingo	Chapter 10 (Diseases of the respiratory system)	Women	4.8	10	0.9019
State of Mexico	Jilotzingo	Chapter 5 (Mental and behavioural disorders)	Men	2.0	4	0.7443
State of Mexico	Juchitepec	Chapter 9 (Diseases of the circulatory system)	Men	12.7	10	0.2408
State of Mexico	Juchitepec	Chapter 9 (Diseases of the circulatory system)	Women	12.4	10	0.2605
State of Mexico	Juchitepec	Chapter 4 (Endocrine, nutritional and metabolic diseases)	Men	9.8	10	0.4560
State of Mexico	Juchitepec	Chapter 4 (Endocrine, nutritional and metabolic diseases)	Women	19.1	10	**0.0394***
State of Mexico	Juchitepec	Chapter 6 (Diseases of the nervous system)	Men	2.6	10	0.9894
State of Mexico	Juchitepec	Chapter 6 (Diseases of the nervous system)	Women	5.6	5	0.3445
State of Mexico	Juchitepec	Chapter 10 (Diseases of the respiratory system)	Men	10.6	10	0.3862
State of Mexico	Juchitepec	Chapter 10 (Diseases of the respiratory system)	Women	8.4	10	0.5887
State of Mexico	Juchitepec	Chapter 5 (Mental and behavioural disorders)	Men	3.4	4	0.5001
State of Mexico	Melchor Ocampo	Chapter 9 (Diseases of the circulatory system)	Men	6.5	10	0.7690
State of Mexico	Melchor Ocampo	Chapter 9 (Diseases of the circulatory system)	Women	10.2	10	0.4241
State of Mexico	Melchor Ocampo	Chapter 4 (Endocrine, nutritional and metabolic diseases)	Men	13.2	10	0.2145
State of Mexico	Melchor Ocampo	Chapter 4 (Endocrine, nutritional and metabolic diseases)	Women	11.2	10	0.3441
State of Mexico	Melchor Ocampo	Chapter 6 (Diseases of the nervous system)	Men	11.7	10	0.3069
State of Mexico	Melchor Ocampo	Chapter 6 (Diseases of the nervous system)	Women	5.3	10	0.8735
State of Mexico	Melchor Ocampo	Chapter 10 (Diseases of the respiratory system)	Men	11.8	10	0.2969
State of Mexico	Melchor Ocampo	Chapter 10 (Diseases of the respiratory system)	Women	10.7	10	0.3812
State of Mexico	Melchor Ocampo	Chapter 5 (Mental and behavioural disorders)	Men	9.0	8	0.3426
State of Mexico	Naucalpan de Juárez	Chapter 9 (Diseases of the circulatory system)	Men	5.7	10	0.8432

State of Mexico	Naucalpan de Juárez	Chapter 9 (Diseases of the circulatory system)	Women	11.9	10	0.2887
State of Mexico	Naucalpan de Juárez	Chapter 4 (Endocrine, nutritional and metabolic diseases)	Men	10.9	10	0.3647
State of Mexico	Naucalpan de Juárez	Chapter 4 (Endocrine, nutritional and metabolic diseases)	Women	17.4	10	0.0655
State of Mexico	Naucalpan de Juárez	Chapter 6 (Diseases of the nervous system)	Men	8.5	10	0.5773
State of Mexico	Naucalpan de Juárez	Chapter 6 (Diseases of the nervous system)	Women	12.9	10	0.2306
State of Mexico	Naucalpan de Juárez	Chapter 10 (Diseases of the respiratory system)	Men	14.8	10	0.1409
State of Mexico	Naucalpan de Juárez	Chapter 10 (Diseases of the respiratory system)	Women	12.6	10	0.2441
State of Mexico	Naucalpan de Juárez	Chapter 5 (Mental and behavioural disorders)	Men	12.5	10	0.2500
State of Mexico	Naucalpan de Juárez	Chapter 5 (Mental and behavioural disorders)	Women	12.2	10	0.2718
State of Mexico	Nezahualcóyotl	Chapter 9 (Diseases of the circulatory system)	Men	13.1	10	0.2163
State of Mexico	Nezahualcóyotl	Chapter 9 (Diseases of the circulatory system)	Women	14.7	10	0.1425
State of Mexico	Nezahualcóyotl	Chapter 4 (Endocrine, nutritional and metabolic diseases)	Men	7.9	10	0.6389
State of Mexico	Nezahualcóyotl	Chapter 4 (Endocrine, nutritional and metabolic diseases)	Women	16.5	10	0.0851
State of Mexico	Nezahualcóyotl	Chapter 6 (Diseases of the nervous system)	Men	5.5	10	0.8564
State of Mexico	Nezahualcóyotl	Chapter 6 (Diseases of the nervous system)	Women	7.5	10	0.6790
State of Mexico	Nezahualcóyotl	Chapter 10 (Diseases of the respiratory system)	Men	7.2	10	0.7077
State of Mexico	Nezahualcóyotl	Chapter 10 (Diseases of the respiratory system)	Women	38.4	10	**0.0000***
State of Mexico	Nezahualcóyotl	Chapter 5 (Mental and behavioural disorders)	Men	6.7	10	0.7530
State of Mexico	Nezahualcóyotl	Chapter 5 (Mental and behavioural disorders)	Women	13.1	10	0.2206
State of Mexico	Nextlalpan	Chapter 9 (Diseases of the circulatory system)	Men	17.5	10	0.0646
State of Mexico	Nextlalpan	Chapter 9 (Diseases of the circulatory system)	Women	7.1	10	0.7132
State of Mexico	Nextlalpan	Chapter 4 (Endocrine, nutritional and metabolic diseases)	Men	9.5	10	0.4818
State of Mexico	Nextlalpan	Chapter 4 (Endocrine, nutritional and metabolic diseases)	Women	8.4	10	0.5856
State of Mexico	Nextlalpan	Chapter 6 (Diseases of the nervous system)	Men	6.3	6	0.3883
State of Mexico	Nextlalpan	Chapter 10 (Diseases of the respiratory system)	Men	9.4	10	0.4982
State of Mexico	Nextlalpan	Chapter 10 (Diseases of the respiratory system)	Women	13.8	10	0.1828
State of Mexico	Nicolás Romero	Chapter 9 (Diseases of the circulatory system)	Men	13.6	10	0.1910
State of Mexico	Nicolás Romero	Chapter 9 (Diseases of the circulatory system)	Women	12.0	10	0.2851
State of Mexico	Nicolás Romero	Chapter 4 (Endocrine, nutritional and metabolic diseases)	Men	11.8	10	0.2963
State of Mexico	Nicolás Romero	Chapter 4 (Endocrine, nutritional and metabolic diseases)	Women	11.5	10	0.3232
State of Mexico	Nicolás Romero	Chapter 6 (Diseases of the nervous system)	Men	12.9	10	0.2282
State of Mexico	Nicolás Romero	Chapter 6 (Diseases of the nervous system)	Women	7.8	10	0.6456
State of Mexico	Nicolás Romero	Chapter 10 (Diseases of the respiratory system)	Men	20.8	10	**0.0229***
State of Mexico	Nicolás Romero	Chapter 10 (Diseases of the respiratory system)	Women	5.4	10	0.8603
State of Mexico	Nicolás Romero	Chapter 5 (Mental and behavioural disorders)	Men	11.1	10	0.3520
State of Mexico	Nicolás Romero	Chapter 5 (Mental and behavioural disorders)	Women	3.3	10	0.9731
State of Mexico	Nopaltepec	Chapter 9 (Diseases of the circulatory system)	Men	12.4	10	0.2614
State of Mexico	Nopaltepec	Chapter 9 (Diseases of the circulatory system)	Women	16.5	10	0.0861
State of Mexico	Nopaltepec	Chapter 4 (Endocrine, nutritional and metabolic diseases)	Men	12.8	10	0.2352
State of Mexico	Nopaltepec	Chapter 4 (Endocrine, nutritional and metabolic diseases)	Women	10.6	10	0.3909
State of Mexico	Nopaltepec	Chapter 10 (Diseases of the respiratory system)	Men	8.1	10	0.6143
State of Mexico	Nopaltepec	Chapter 10 (Diseases of the respiratory system)	Women	7.7	10	0.6568
State of Mexico	Otumba	Chapter 9 (Diseases of the circulatory system)	Men	4.9	10	0.8982

State of Mexico	Otumba	Chapter 9 (Diseases of the circulatory system)	Women	9.0	10	0.5309
State of Mexico	Otumba	Chapter 4 (Endocrine, nutritional and metabolic diseases)	Men	7.4	10	0.6905
State of Mexico	Otumba	Chapter 4 (Endocrine, nutritional and metabolic diseases)	Women	10.3	10	0.4152
State of Mexico	Otumba	Chapter 6 (Diseases of the nervous system)	Women	10.3	8	0.2461
State of Mexico	Otumba	Chapter 6 (Diseases of the nervous system)	Men	6.4	7	0.4918
State of Mexico	Otumba	Chapter 10 (Diseases of the respiratory system)	Men	14.5	10	0.1510
State of Mexico	Otumba	Chapter 10 (Diseases of the respiratory system)	Women	5.4	10	0.8640
State of Mexico	Otumba	Chapter 5 (Mental and behavioural disorders)	Men	4.7	9	0.8628
State of Mexico	Otumba	Chapter 5 (Mental and behavioural disorders)	Women	9.2	5	0.0998
State of Mexico	Ozumba	Chapter 9 (Diseases of the circulatory system)	Men	10.8	10	0.3730
State of Mexico	Ozumba	Chapter 9 (Diseases of the circulatory system)	Women	2.7	10	0.9875
State of Mexico	Ozumba	Chapter 4 (Endocrine, nutritional and metabolic diseases)	Men	10.3	10	0.4107
State of Mexico	Ozumba	Chapter 4 (Endocrine, nutritional and metabolic diseases)	Women	9.8	10	0.4570
State of Mexico	Ozumba	Chapter 6 (Diseases of the nervous system)	Women	14.9	9	0.0934
State of Mexico	Ozumba	Chapter 6 (Diseases of the nervous system)	Men	9.2	10	0.5137
State of Mexico	Ozumba	Chapter 10 (Diseases of the respiratory system)	Men	13.8	10	0.1824
State of Mexico	Ozumba	Chapter 10 (Diseases of the respiratory system)	Women	14.0	10	0.1737
State of Mexico	Ozumba	Chapter 5 (Mental and behavioural disorders)	Men	4.1	10	0.9434
State of Mexico	Papalotla	Chapter 9 (Diseases of the circulatory system)	Men	7.3	10	0.7014
State of Mexico	Papalotla	Chapter 9 (Diseases of the circulatory system)	Women	9.4	10	0.4950
State of Mexico	Papalotla	Chapter 4 (Endocrine, nutritional and metabolic diseases)	Men	12.2	10	0.2731
State of Mexico	Papalotla	Chapter 4 (Endocrine, nutritional and metabolic diseases)	Women	19.5	10	**0.0348***
State of Mexico	Papalotla	Chapter 6 (Diseases of the nervous system)	Women	5.8	4	0.2182
State of Mexico	Papalotla	Chapter 10 (Diseases of the respiratory system)	Men	15.7	10	0.1077
State of Mexico	Papalotla	Chapter 10 (Diseases of the respiratory system)	Women	7.9	8	0.4449
State of Mexico	La Paz	Chapter 9 (Diseases of the circulatory system)	Men	8.1	10	0.6220
State of Mexico	La Paz	Chapter 9 (Diseases of the circulatory system)	Women	15.7	10	0.1098
State of Mexico	La Paz	Chapter 4 (Endocrine, nutritional and metabolic diseases)	Men	8.8	10	0.5477
State of Mexico	La Paz	Chapter 4 (Endocrine, nutritional and metabolic diseases)	Women	9.5	10	0.4858
State of Mexico	La Paz	Chapter 6 (Diseases of the nervous system)	Men	7.7	10	0.6562
State of Mexico	La Paz	Chapter 6 (Diseases of the nervous system)	Women	16.3	10	0.0922
State of Mexico	La Paz	Chapter 10 (Diseases of the respiratory system)	Men	6.7	10	0.7557
State of Mexico	La Paz	Chapter 10 (Diseases of the respiratory system)	Women	13.5	10	0.1947
State of Mexico	La Paz	Chapter 5 (Mental and behavioural disorders)	Men	7.0	10	0.7274
State of Mexico	La Paz	Chapter 5 (Mental and behavioural disorders)	Women	7.6	10	0.6721
State of Mexico	San Martín de las Pirámides	Chapter 9 (Diseases of the circulatory system)	Men	5.6	10	0.8452
State of Mexico	San Martín de las Pirámides	Chapter 9 (Diseases of the circulatory system)	Women	18.0	10	0.0547
State of Mexico	San Martín de las Pirámides	Chapter 4 (Endocrine, nutritional and metabolic diseases)	Men	12.0	10	0.2848
State of Mexico	San Martín de las Pirámides	Chapter 4 (Endocrine, nutritional and metabolic diseases)	Women	18.4	10	0.0491
State of Mexico	San Martín de las Pirámides	Chapter 6 (Diseases of the nervous system)	Women	7.7	10	0.6587
State of Mexico	San Martín de las Pirámides	Chapter 6 (Diseases of the nervous system)	Men	5.9	7	0.5509
State of Mexico	San Martín de las Pirámides	Chapter 10 (Diseases of the respiratory system)	Men	4.9	10	0.8968
State of Mexico	San Martín de las Pirámides	Chapter 10 (Diseases of the respiratory system)	Women	3.9	10	0.9522

State of Mexico	San Martín de las Pirámides	Chapter 5 (Mental and behavioural disorders)	Men	3.2	5	0.6654
State of Mexico	Tecámac	Chapter 9 (Diseases of the circulatory system)	Men	39.7	10	**0.0000***
State of Mexico	Tecámac	Chapter 9 (Diseases of the circulatory system)	Women	9.3	10	0.4994
State of Mexico	Tecámac	Chapter 4 (Endocrine, nutritional and metabolic diseases)	Men	4.7	10	0.9125
State of Mexico	Tecámac	Chapter 4 (Endocrine, nutritional and metabolic diseases)	Women	10.5	10	0.3996
State of Mexico	Tecámac	Chapter 6 (Diseases of the nervous system)	Men	19.0	10	**0.0402***
State of Mexico	Tecámac	Chapter 6 (Diseases of the nervous system)	Women	5.1	10	0.8848
State of Mexico	Tecámac	Chapter 10 (Diseases of the respiratory system)	Men	8.2	10	0.6074
State of Mexico	Tecámac	Chapter 10 (Diseases of the respiratory system)	Women	10.2	10	0.4207
State of Mexico	Tecámac	Chapter 5 (Mental and behavioural disorders)	Men	6.5	10	0.7737
State of Mexico	Tecámac	Chapter 5 (Mental and behavioural disorders)	Women	9.5	10	0.4835
State of Mexico	Temamatla	Chapter 9 (Diseases of the circulatory system)	Men	4.9	10	0.8946
State of Mexico	Temamatla	Chapter 9 (Diseases of the circulatory system)	Women	10.1	10	0.4354
State of Mexico	Temamatla	Chapter 4 (Endocrine, nutritional and metabolic diseases)	Women	9.5	10	0.4860
State of Mexico	Temamatla	Chapter 4 (Endocrine, nutritional and metabolic diseases)	Men	13.0	10	0.2251
State of Mexico	Temamatla	Chapter 10 (Diseases of the respiratory system)	Men	11.3	10	0.3365
State of Mexico	Temamatla	Chapter 10 (Diseases of the respiratory system)	Women	21.0	10	**0.0212***
State of Mexico	Temascalapa	Chapter 9 (Diseases of the circulatory system)	Men	12.4	10	0.2586
State of Mexico	Temascalapa	Chapter 9 (Diseases of the circulatory system)	Women	17.0	10	0.0747
State of Mexico	Temascalapa	Chapter 4 (Endocrine, nutritional and metabolic diseases)	Men	13.1	10	0.2208
State of Mexico	Temascalapa	Chapter 4 (Endocrine, nutritional and metabolic diseases)	Women	8.2	10	0.6122
State of Mexico	Temascalapa	Chapter 6 (Diseases of the nervous system)	Women	10.3	10	0.4124
State of Mexico	Temascalapa	Chapter 6 (Diseases of the nervous system)	Men	9.5	10	0.4820
State of Mexico	Temascalapa	Chapter 10 (Diseases of the respiratory system)	Men	9.5	10	0.4895
State of Mexico	Temascalapa	Chapter 10 (Diseases of the respiratory system)	Women	6.5	10	0.7715
State of Mexico	Temascalapa	Chapter 5 (Mental and behavioural disorders)	Men	6.0	5	0.3079
State of Mexico	Tenango del Aire	Chapter 9 (Diseases of the circulatory system)	Men	14.1	10	0.1668
State of Mexico	Tenango del Aire	Chapter 9 (Diseases of the circulatory system)	Women	5.1	10	0.8864
State of Mexico	Tenango del Aire	Chapter 4 (Endocrine, nutritional and metabolic diseases)	Men	4.1	10	0.9428
State of Mexico	Tenango del Aire	Chapter 4 (Endocrine, nutritional and metabolic diseases)	Women	11.3	10	0.3328
State of Mexico	Tenango del Aire	Chapter 10 (Diseases of the respiratory system)	Men	8.1	10	0.6184
State of Mexico	Tenango del Aire	Chapter 10 (Diseases of the respiratory system)	Women	9.5	10	0.4835
State of Mexico	Teoloyucan	Chapter 9 (Diseases of the circulatory system)	Men	6.9	10	0.7392
State of Mexico	Teoloyucan	Chapter 9 (Diseases of the circulatory system)	Women	6.2	10	0.8003
State of Mexico	Teoloyucan	Chapter 4 (Endocrine, nutritional and metabolic diseases)	Men	16.9	10	0.0766
State of Mexico	Teoloyucan	Chapter 4 (Endocrine, nutritional and metabolic diseases)	Women	14.3	10	0.1602
State of Mexico	Teoloyucan	Chapter 6 (Diseases of the nervous system)	Women	7.2	10	0.7092
State of Mexico	Teoloyucan	Chapter 6 (Diseases of the nervous system)	Men	13.3	10	0.2064
State of Mexico	Teoloyucan	Chapter 10 (Diseases of the respiratory system)	Men	6.8	10	0.7423
State of Mexico	Teoloyucan	Chapter 10 (Diseases of the respiratory system)	Women	7.2	10	0.7068
State of Mexico	Teoloyucan	Chapter 5 (Mental and behavioural disorders)	Men	19.7	10	**0.0322***
State of Mexico	Teotihuacán	Chapter 9 (Diseases of the circulatory system)	Men	10.6	10	0.3890
State of Mexico	Teotihuacán	Chapter 9 (Diseases of the circulatory system)	Women	11.3	10	0.3360

State of Mexico	Teotihuacán	Chapter 4 (Endocrine, nutritional and metabolic diseases)	Men	8.0	10	0.6272
State of Mexico	Teotihuacán	Chapter 4 (Endocrine, nutritional and metabolic diseases)	Women	4.5	10	0.9234
State of Mexico	Teotihuacán	Chapter 6 (Diseases of the nervous system)	Men	14.8	10	0.1388
State of Mexico	Teotihuacán	Chapter 6 (Diseases of the nervous system)	Women	7.2	7	0.4129
State of Mexico	Teotihuacán	Chapter 10 (Diseases of the respiratory system)	Men	10.3	10	0.4149
State of Mexico	Teotihuacán	Chapter 10 (Diseases of the respiratory system)	Women	8.8	10	0.5495
State of Mexico	Teotihuacán	Chapter 5 (Mental and behavioural disorders)	Men	5.4	8	0.7185
State of Mexico	Tepetlaoxtoc	Chapter 9 (Diseases of the circulatory system)	Men	18.6	10	**0.0462***
State of Mexico	Tepetlaoxtoc	Chapter 9 (Diseases of the circulatory system)	Women	13.4	10	0.1999
State of Mexico	Tepetlaoxtoc	Chapter 4 (Endocrine, nutritional and metabolic diseases)	Men	17.1	10	0.0722
State of Mexico	Tepetlaoxtoc	Chapter 4 (Endocrine, nutritional and metabolic diseases)	Women	6.7	10	0.7525
State of Mexico	Tepetlaoxtoc	Chapter 6 (Diseases of the nervous system)	Men	14.3	10	0.1608
State of Mexico	Tepetlaoxtoc	Chapter 6 (Diseases of the nervous system)	Women	6.6	7	0.4764
State of Mexico	Tepetlaoxtoc	Chapter 10 (Diseases of the respiratory system)	Men	15.2	10	0.1256
State of Mexico	Tepetlaoxtoc	Chapter 10 (Diseases of the respiratory system)	Women	10.3	10	0.4140
State of Mexico	Tepetlaoxtoc	Chapter 5 (Mental and behavioural disorders)	Men	4.7	6	0.5802
State of Mexico	Tepetlixpa	Chapter 9 (Diseases of the circulatory system)	Men	10.3	10	0.4186
State of Mexico	Tepetlixpa	Chapter 9 (Diseases of the circulatory system)	Women	13.1	10	0.2205
State of Mexico	Tepetlixpa	Chapter 4 (Endocrine, nutritional and metabolic diseases)	Men	6.8	10	0.7441
State of Mexico	Tepetlixpa	Chapter 4 (Endocrine, nutritional and metabolic diseases)	Women	11.8	10	0.3021
State of Mexico	Tepetlixpa	Chapter 6 (Diseases of the nervous system)	Men	4.8	10	0.9023
State of Mexico	Tepetlixpa	Chapter 6 (Diseases of the nervous system)	Women	2.1	6	0.9063
State of Mexico	Tepetlixpa	Chapter 10 (Diseases of the respiratory system)	Men	15.1	10	0.1269
State of Mexico	Tepetlixpa	Chapter 10 (Diseases of the respiratory system)	Women	27.5	10	**0.0022***
State of Mexico	Tepetlixpa	Chapter 5 (Mental and behavioural disorders)	Men	8.9	10	0.5396
State of Mexico	Tepotzotlán	Chapter 9 (Diseases of the circulatory system)	Men	18.9	10	**0.0414***
State of Mexico	Tepotzotlán	Chapter 9 (Diseases of the circulatory system)	Women	13.6	10	0.1942
State of Mexico	Tepotzotlán	Chapter 4 (Endocrine, nutritional and metabolic diseases)	Men	17.0	10	0.0741
State of Mexico	Tepotzotlán	Chapter 4 (Endocrine, nutritional and metabolic diseases)	Women	5.3	10	0.8679
State of Mexico	Tepotzotlán	Chapter 6 (Diseases of the nervous system)	Men	20.6	10	**0.0242***
State of Mexico	Tepotzotlán	Chapter 6 (Diseases of the nervous system)	Women	6.6	10	0.7608
State of Mexico	Tepotzotlán	Chapter 10 (Diseases of the respiratory system)	Men	10.8	10	0.3695
State of Mexico	Tepotzotlán	Chapter 10 (Diseases of the respiratory system)	Women	11.8	10	0.2975
State of Mexico	Tepotzotlán	Chapter 5 (Mental and behavioural disorders)	Men	6.9	10	0.7311
State of Mexico	Tequixquiac	Chapter 9 (Diseases of the circulatory system)	Men	12.5	10	0.2555
State of Mexico	Tequixquiac	Chapter 9 (Diseases of the circulatory system)	Women	12.4	10	0.2585
State of Mexico	Tequixquiac	Chapter 4 (Endocrine, nutritional and metabolic diseases)	Women	9.5	10	0.4856
State of Mexico	Tequixquiac	Chapter 4 (Endocrine, nutritional and metabolic diseases)	Men	10.8	10	0.3707
State of Mexico	Tequixquiac	Chapter 6 (Diseases of the nervous system)	Women	8.3	8	0.4067
State of Mexico	Tequixquiac	Chapter 6 (Diseases of the nervous system)	Men	13.2	9	0.1557
State of Mexico	Tequixquiac	Chapter 10 (Diseases of the respiratory system)	Men	8.8	10	0.5476
State of Mexico	Tequixquiac	Chapter 10 (Diseases of the respiratory system)	Women	29.8	10	**0.0009***
State of Mexico	Tequixquiac	Chapter 5 (Mental and behavioural disorders)	Men	6.0	5	0.3079

State of Mexico	Texcoco	Chapter 9 (Diseases of the circulatory system)	Men	10.4	10	0.4038
State of Mexico	Texcoco	Chapter 9 (Diseases of the circulatory system)	Women	16.0	10	0.0987
State of Mexico	Texcoco	Chapter 4 (Endocrine, nutritional and metabolic diseases)	Men	8.0	10	0.6303
State of Mexico	Texcoco	Chapter 4 (Endocrine, nutritional and metabolic diseases)	Women	16.4	10	0.0887
State of Mexico	Texcoco	Chapter 6 (Diseases of the nervous system)	Men	11.0	10	0.3562
State of Mexico	Texcoco	Chapter 6 (Diseases of the nervous system)	Women	8.1	10	0.6150
State of Mexico	Texcoco	Chapter 10 (Diseases of the respiratory system)	Men	12.8	10	0.2327
State of Mexico	Texcoco	Chapter 10 (Diseases of the respiratory system)	Women	7.0	10	0.7285
State of Mexico	Texcoco	Chapter 5 (Mental and behavioural disorders)	Men	11.5	10	0.3170
State of Mexico	Texcoco	Chapter 5 (Mental and behavioural disorders)	Women	10.9	10	0.3625
State of Mexico	Tezoyuca	Chapter 9 (Diseases of the circulatory system)	Men	8.6	10	0.5712
State of Mexico	Tezoyuca	Chapter 9 (Diseases of the circulatory system)	Women	8.2	10	0.6120
State of Mexico	Tezoyuca	Chapter 4 (Endocrine, nutritional and metabolic diseases)	Men	4.1	10	0.9435
State of Mexico	Tezoyuca	Chapter 4 (Endocrine, nutritional and metabolic diseases)	Women	9.9	10	0.4490
State of Mexico	Tezoyuca	Chapter 6 (Diseases of the nervous system)	Men	5.0	8	0.7564
State of Mexico	Tezoyuca	Chapter 6 (Diseases of the nervous system)	Women	3.0	8	0.9317
State of Mexico	Tezoyuca	Chapter 10 (Diseases of the respiratory system)	Men	11.2	10	0.3386
State of Mexico	Tezoyuca	Chapter 10 (Diseases of the respiratory system)	Women	13.2	10	0.2153
State of Mexico	Tezoyuca	Chapter 5 (Mental and behavioural disorders)	Men	4.6	7	0.7057
State of Mexico	Tlalmanalco	Chapter 9 (Diseases of the circulatory system)	Men	36.0	10	**0.0001***
State of Mexico	Tlalmanalco	Chapter 9 (Diseases of the circulatory system)	Women	13.2	10	0.2119
State of Mexico	Tlalmanalco	Chapter 4 (Endocrine, nutritional and metabolic diseases)	Men	9.0	10	0.5279
State of Mexico	Tlalmanalco	Chapter 4 (Endocrine, nutritional and metabolic diseases)	Women	8.1	10	0.6233
State of Mexico	Tlalmanalco	Chapter 6 (Diseases of the nervous system)	Men	10.7	10	0.3784
State of Mexico	Tlalmanalco	Chapter 6 (Diseases of the nervous system)	Women	5.2	10	0.8743
State of Mexico	Tlalmanalco	Chapter 10 (Diseases of the respiratory system)	Men	10.0	10	0.4373
State of Mexico	Tlalmanalco	Chapter 10 (Diseases of the respiratory system)	Women	8.0	10	0.6327
State of Mexico	Tlalmanalco	Chapter 5 (Mental and behavioural disorders)	Men	14.6	10	0.1460
State of Mexico	Tlalnepantla de Baz	Chapter 9 (Diseases of the circulatory system)	Men	9.7	10	0.4693
State of Mexico	Tlalnepantla de Baz	Chapter 9 (Diseases of the circulatory system)	Women	15.4	10	0.1197
State of Mexico	Tlalnepantla de Baz	Chapter 4 (Endocrine, nutritional and metabolic diseases)	Men	5.8	10	0.8293
State of Mexico	Tlalnepantla de Baz	Chapter 4 (Endocrine, nutritional and metabolic diseases)	Women	7.7	10	0.6539
State of Mexico	Tlalnepantla de Baz	Chapter 6 (Diseases of the nervous system)	Men	4.2	10	0.9375
State of Mexico	Tlalnepantla de Baz	Chapter 6 (Diseases of the nervous system)	Women	8.0	10	0.6251
State of Mexico	Tlalnepantla de Baz	Chapter 10 (Diseases of the respiratory system)	Men	12.7	10	0.2389
State of Mexico	Tlalnepantla de Baz	Chapter 10 (Diseases of the respiratory system)	Women	20.6	10	**0.0244***
State of Mexico	Tlalnepantla de Baz	Chapter 5 (Mental and behavioural disorders)	Men	9.0	10	0.5347
State of Mexico	Tlalnepantla de Baz	Chapter 5 (Mental and behavioural disorders)	Women	10.1	10	0.4358
State of Mexico	Tultepec	Chapter 9 (Diseases of the circulatory system)	Men	13.6	10	0.1907
State of Mexico	Tultepec	Chapter 9 (Diseases of the circulatory system)	Women	12.8	10	0.2367
State of Mexico	Tultepec	Chapter 4 (Endocrine, nutritional and metabolic diseases)	Men	14.9	10	0.1358
State of Mexico	Tultepec	Chapter 4 (Endocrine, nutritional and metabolic diseases)	Women	10.8	10	0.3700
State of Mexico	Tultepec	Chapter 6 (Diseases of the nervous system)	Men	8.5	10	0.5764

State of Mexico	Tultepec	Chapter 6 (Diseases of the nervous system)	Women	13.8	10	0.1803
State of Mexico	Tultepec	Chapter 10 (Diseases of the respiratory system)	Men	10.3	10	0.4163
State of Mexico	Tultepec	Chapter 10 (Diseases of the respiratory system)	Women	9.5	10	0.4887
State of Mexico	Tultepec	Chapter 5 (Mental and behavioural disorders)	Men	7.1	10	0.7173
State of Mexico	Tultepec	Chapter 5 (Mental and behavioural disorders)	Women	5.8	4	0.2185
State of Mexico	Tultitlán	Chapter 9 (Diseases of the circulatory system)	Men	3.2	10	0.9763
State of Mexico	Tultitlán	Chapter 9 (Diseases of the circulatory system)	Women	8.3	10	0.5998
State of Mexico	Tultitlán	Chapter 4 (Endocrine, nutritional and metabolic diseases)	Men	2.9	10	0.9832
State of Mexico	Tultitlán	Chapter 4 (Endocrine, nutritional and metabolic diseases)	Women	17.4	10	0.0651
State of Mexico	Tultitlán	Chapter 6 (Diseases of the nervous system)	Men	7.1	10	0.7177
State of Mexico	Tultitlán	Chapter 6 (Diseases of the nervous system)	Women	11.6	10	0.3100
State of Mexico	Tultitlán	Chapter 10 (Diseases of the respiratory system)	Men	10.5	10	0.3994
State of Mexico	Tultitlán	Chapter 10 (Diseases of the respiratory system)	Women	12.2	10	0.2716
State of Mexico	Tultitlán	Chapter 5 (Mental and behavioural disorders)	Men	12.5	10	0.2523
State of Mexico	Tultitlán	Chapter 5 (Mental and behavioural disorders)	Women	3.7	10	0.9608
State of Mexico	Villa del Carbón	Chapter 9 (Diseases of the circulatory system)	Men	10.8	10	0.3697
State of Mexico	Villa del Carbón	Chapter 9 (Diseases of the circulatory system)	Women	4.7	10	0.9098
State of Mexico	Villa del Carbón	Chapter 4 (Endocrine, nutritional and metabolic diseases)	Men	9.3	10	0.5050
State of Mexico	Villa del Carbón	Chapter 4 (Endocrine, nutritional and metabolic diseases)	Women	11.0	10	0.3544
State of Mexico	Villa del Carbón	Chapter 6 (Diseases of the nervous system)	Men	11.2	10	0.3440
State of Mexico	Villa del Carbón	Chapter 6 (Diseases of the nervous system)	Women	5.4	10	0.8655
State of Mexico	Villa del Carbón	Chapter 10 (Diseases of the respiratory system)	Men	14.9	10	0.1341
State of Mexico	Villa del Carbón	Chapter 10 (Diseases of the respiratory system)	Women	9.0	10	0.5304
State of Mexico	Villa del Carbón	Chapter 5 (Mental and behavioural disorders)	Men	11.4	10	0.3243
State of Mexico	Villa del Carbón	Chapter 5 (Mental and behavioural disorders)	Women	5.2	4	0.2637
State of Mexico	Zumpango	Chapter 9 (Diseases of the circulatory system)	Men	6.7	10	0.7496
State of Mexico	Zumpango	Chapter 9 (Diseases of the circulatory system)	Women	7.6	10	0.6703
State of Mexico	Zumpango	Chapter 4 (Endocrine, nutritional and metabolic diseases)	Men	5.7	10	0.8411
State of Mexico	Zumpango	Chapter 4 (Endocrine, nutritional and metabolic diseases)	Women	9.3	10	0.5067
State of Mexico	Zumpango	Chapter 6 (Diseases of the nervous system)	Men	16.0	10	0.0986
State of Mexico	Zumpango	Chapter 6 (Diseases of the nervous system)	Women	7.7	10	0.6550
State of Mexico	Zumpango	Chapter 10 (Diseases of the respiratory system)	Men	27.2	10	**0.0025***
State of Mexico	Zumpango	Chapter 10 (Diseases of the respiratory system)	Women	7.3	10	0.6994
State of Mexico	Zumpango	Chapter 5 (Mental and behavioural disorders)	Men	8.9	10	0.5429
State of Mexico	Zumpango	Chapter 5 (Mental and behavioural disorders)	Women	7.6	8	0.4756
State of Mexico	Cuautitlán Izcalli	Chapter 9 (Diseases of the circulatory system)	Men	16.5	10	0.0859
State of Mexico	Cuautitlán Izcalli	Chapter 9 (Diseases of the circulatory system)	Women	18.3	10	0.0506
State of Mexico	Cuautitlán Izcalli	Chapter 4 (Endocrine, nutritional and metabolic diseases)	Men	8.1	10	0.6227
State of Mexico	Cuautitlán Izcalli	Chapter 4 (Endocrine, nutritional and metabolic diseases)	Women	5.6	10	0.8448
State of Mexico	Cuautitlán Izcalli	Chapter 6 (Diseases of the nervous system)	Men	22.2	10	**0.0140***
State of Mexico	Cuautitlán Izcalli	Chapter 6 (Diseases of the nervous system)	Women	12.1	10	0.2812
State of Mexico	Cuautitlán Izcalli	Chapter 10 (Diseases of the respiratory system)	Men	7.8	10	0.6487
State of Mexico	Cuautitlán Izcalli	Chapter 10 (Diseases of the respiratory system)	Women	9.2	10	0.5145

State of Mexico	Cuautitlán Izcalli	Chapter 5 (Mental and behavioural disorders)	Men	1.8	10	0.9977
State of Mexico	Cuautitlán Izcalli	Chapter 5 (Mental and behavioural disorders)	Women	9.1	10	0.5204
State of Mexico	Valle de Chalco Solidaridad	Chapter 9 (Diseases of the circulatory system)	Men	6.9	10	0.7372
State of Mexico	Valle de Chalco Solidaridad	Chapter 9 (Diseases of the circulatory system)	Women	18.1	10	0.0533
State of Mexico	Valle de Chalco Solidaridad	Chapter 4 (Endocrine, nutritional and metabolic diseases)	Men	7.0	10	0.7218
State of Mexico	Valle de Chalco Solidaridad	Chapter 4 (Endocrine, nutritional and metabolic diseases)	Women	13.6	10	0.1935
State of Mexico	Valle de Chalco Solidaridad	Chapter 6 (Diseases of the nervous system)	Men	17.7	10	0.0610
State of Mexico	Valle de Chalco Solidaridad	Chapter 6 (Diseases of the nervous system)	Women	4.5	10	0.9205
State of Mexico	Valle de Chalco Solidaridad	Chapter 10 (Diseases of the respiratory system)	Men	8.5	10	0.5820
State of Mexico	Valle de Chalco Solidaridad	Chapter 10 (Diseases of the respiratory system)	Women	9.5	10	0.4881
State of Mexico	Valle de Chalco Solidaridad	Chapter 5 (Mental and behavioural disorders)	Men	9.1	10	0.5265
State of Mexico	Valle de Chalco Solidaridad	Chapter 5 (Mental and behavioural disorders)	Women	11.0	9	0.2782
State of Mexico	Tonanitla	Chapter 9 (Diseases of the circulatory system)	Men	15.3	10	0.1217
State of Mexico	Tonanitla	Chapter 9 (Diseases of the circulatory system)	Women	19.0	10	**0.0399***
State of Mexico	Tonanitla	Chapter 4 (Endocrine, nutritional and metabolic diseases)	Men	5.7	10	0.8390
State of Mexico	Tonanitla	Chapter 4 (Endocrine, nutritional and metabolic diseases)	Women	8.0	10	0.6277
State of Mexico	Tonanitla	Chapter 10 (Diseases of the respiratory system)	Men	6.5	10	0.7720
State of Mexico	Tonanitla	Chapter 10 (Diseases of the respiratory system)	Women	9.6	10	0.4760

Table S6. Ljung–Box test results for residual autocorrelation by ICD-10 chapter and age group at the municipality level. (*) indicates statistically significant autocorrelation.

State	Municipality	ICD-10 Chapter	Age gp	Ljung–Box *Q**	*df*	*p-value*
Mexico City	Azcapotzalco	Chapter 9 (Diseases of the circulatory system)	0-4	11.3	10	0.3376
Mexico City	Azcapotzalco	Chapter 9 (Diseases of the circulatory system)	5-14	10.1	10	0.4314
Mexico City	Azcapotzalco	Chapter 9 (Diseases of the circulatory system)	15-24	13.2	10	0.2145
Mexico City	Azcapotzalco	Chapter 9 (Diseases of the circulatory system)	25-34	8.2	10	0.6047
Mexico City	Azcapotzalco	Chapter 9 (Diseases of the circulatory system)	35-44	10.3	10	0.4113
Mexico City	Azcapotzalco	Chapter 9 (Diseases of the circulatory system)	45-54	7.5	10	0.6764
Mexico City	Azcapotzalco	Chapter 9 (Diseases of the circulatory system)	55-64	6.3	10	0.7852
Mexico City	Azcapotzalco	Chapter 9 (Diseases of the circulatory system)	65+	9.8	10	0.4547
Mexico City	Azcapotzalco	Chapter 4 (Endocrine, nutritional and metabolic diseases)	0-4	13.4	10	0.2027
Mexico City	Azcapotzalco	Chapter 4 (Endocrine, nutritional and metabolic diseases)	15-24	10.3	10	0.4158
Mexico City	Azcapotzalco	Chapter 4 (Endocrine, nutritional and metabolic diseases)	25-34	9.2	10	0.5125
Mexico City	Azcapotzalco	Chapter 4 (Endocrine, nutritional and metabolic diseases)	35-44	5.9	10	0.8223
Mexico City	Azcapotzalco	Chapter 4 (Endocrine, nutritional and metabolic diseases)	45-54	20.0	10	**0.0293***
Mexico City	Azcapotzalco	Chapter 4 (Endocrine, nutritional and metabolic diseases)	55-64	17.4	10	0.0659
Mexico City	Azcapotzalco	Chapter 4 (Endocrine, nutritional and metabolic diseases)	65+	7.5	10	0.6730
Mexico City	Azcapotzalco	Chapter 6 (Diseases of the nervous system)	0-4	8.8	10	0.5520
Mexico City	Azcapotzalco	Chapter 6 (Diseases of the nervous system)	25-34	15.6	10	0.1118
Mexico City	Azcapotzalco	Chapter 6 (Diseases of the nervous system)	35-44	4.7	10	0.9077
Mexico City	Azcapotzalco	Chapter 6 (Diseases of the nervous system)	55-64	8.6	10	0.5697
Mexico City	Azcapotzalco	Chapter 6 (Diseases of the nervous system)	65+	15.9	10	0.1034

Mexico City	Azcapotzalco	Chapter 6 (Diseases of the nervous system)	5-14	11.6	10	0.3121
Mexico City	Azcapotzalco	Chapter 6 (Diseases of the nervous system)	15-24	9.9	10	0.4515
Mexico City	Azcapotzalco	Chapter 6 (Diseases of the nervous system)	45-54	11.1	10	0.3482
Mexico City	Azcapotzalco	Chapter 10 (Diseases of the respiratory system)	0-4	3.6	10	0.9641
Mexico City	Azcapotzalco	Chapter 10 (Diseases of the respiratory system)	5-14	7.1	10	0.7127
Mexico City	Azcapotzalco	Chapter 10 (Diseases of the respiratory system)	15-24	12.3	10	0.2668
Mexico City	Azcapotzalco	Chapter 10 (Diseases of the respiratory system)	25-34	6.4	10	0.7805
Mexico City	Azcapotzalco	Chapter 10 (Diseases of the respiratory system)	35-44	14.9	10	0.1342
Mexico City	Azcapotzalco	Chapter 10 (Diseases of the respiratory system)	45-54	9.1	10	0.5180
Mexico City	Azcapotzalco	Chapter 10 (Diseases of the respiratory system)	55-64	23.6	10	**0.0087***
Mexico City	Azcapotzalco	Chapter 10 (Diseases of the respiratory system)	65+	5.1	10	0.8831
Mexico City	Azcapotzalco	Chapter 5 (Mental and behavioural disorders)	35-44	7.1	10	0.7117
Mexico City	Azcapotzalco	Chapter 5 (Mental and behavioural disorders)	55-64	10.6	10	0.3863
Mexico City	Azcapotzalco	Chapter 5 (Mental and behavioural disorders)	65+	12.4	10	0.2575
Mexico City	Azcapotzalco	Chapter 5 (Mental and behavioural disorders)	25-34	8.9	7	0.2617
Mexico City	Azcapotzalco	Chapter 5 (Mental and behavioural disorders)	45-54	15.2	10	0.1257
Mexico City	Coyoacán	Chapter 9 (Diseases of the circulatory system)	0-4	1.8	8	0.9865
Mexico City	Coyoacán	Chapter 9 (Diseases of the circulatory system)	15-24	11.8	10	0.2977
Mexico City	Coyoacán	Chapter 9 (Diseases of the circulatory system)	25-34	12.5	10	0.2510
Mexico City	Coyoacán	Chapter 9 (Diseases of the circulatory system)	35-44	11.7	10	0.3022
Mexico City	Coyoacán	Chapter 9 (Diseases of the circulatory system)	45-54	15.6	10	0.1125
Mexico City	Coyoacán	Chapter 9 (Diseases of the circulatory system)	55-64	8.4	10	0.5930
Mexico City	Coyoacán	Chapter 9 (Diseases of the circulatory system)	65+	14.1	10	0.1701
Mexico City	Coyoacán	Chapter 4 (Endocrine, nutritional and metabolic diseases)	25-34	14.8	10	0.1386
Mexico City	Coyoacán	Chapter 4 (Endocrine, nutritional and metabolic diseases)	35-44	11.7	10	0.3025
Mexico City	Coyoacán	Chapter 4 (Endocrine, nutritional and metabolic diseases)	45-54	12.9	10	0.2271
Mexico City	Coyoacán	Chapter 4 (Endocrine, nutritional and metabolic diseases)	55-64	8.2	10	0.6071
Mexico City	Coyoacán	Chapter 4 (Endocrine, nutritional and metabolic diseases)	65+	27.0	10	**0.0026***
Mexico City	Coyoacán	Chapter 4 (Endocrine, nutritional and metabolic diseases)	0-4	2.0	10	0.9964
Mexico City	Coyoacán	Chapter 4 (Endocrine, nutritional and metabolic diseases)	5-14	11.6	6	0.0714
Mexico City	Coyoacán	Chapter 4 (Endocrine, nutritional and metabolic diseases)	15-24	4.3	9	0.8888
Mexico City	Coyoacán	Chapter 6 (Diseases of the nervous system)	0-4	8.6	10	0.5748
Mexico City	Coyoacán	Chapter 6 (Diseases of the nervous system)	5-14	18.0	10	0.0548
Mexico City	Coyoacán	Chapter 6 (Diseases of the nervous system)	25-34	7.9	10	0.6391
Mexico City	Coyoacán	Chapter 6 (Diseases of the nervous system)	35-44	10.0	10	0.4436
Mexico City	Coyoacán	Chapter 6 (Diseases of the nervous system)	45-54	7.9	10	0.6348
Mexico City	Coyoacán	Chapter 6 (Diseases of the nervous system)	55-64	8.9	10	0.5405
Mexico City	Coyoacán	Chapter 6 (Diseases of the nervous system)	65+	6.0	10	0.8165
Mexico City	Coyoacán	Chapter 6 (Diseases of the nervous system)	15-24	8.4	10	0.5905
Mexico City	Coyoacán	Chapter 10 (Diseases of the respiratory system)	0-4	18.3	10	0.0504
Mexico City	Coyoacán	Chapter 10 (Diseases of the respiratory system)	15-24	9.1	10	0.5244
Mexico City	Coyoacán	Chapter 10 (Diseases of the respiratory system)	35-44	18.0	10	0.0541
Mexico City	Coyoacán	Chapter 10 (Diseases of the respiratory system)	45-54	46.7	10	**0.0000***

Mexico City	Coyoacán	Chapter 10 (Diseases of the respiratory system)	55-64	8.0	10	0.6275
Mexico City	Coyoacán	Chapter 10 (Diseases of the respiratory system)	65+	5.1	10	0.8857
Mexico City	Coyoacán	Chapter 10 (Diseases of the respiratory system)	25-34	6.0	10	0.8140
Mexico City	Coyoacán	Chapter 10 (Diseases of the respiratory system)	5-14	4.6	8	0.7943
Mexico City	Coyoacán	Chapter 5 (Mental and behavioural disorders)	45-54	10.3	10	0.4142
Mexico City	Coyoacán	Chapter 5 (Mental and behavioural disorders)	55-64	9.1	10	0.5213
Mexico City	Coyoacán	Chapter 5 (Mental and behavioural disorders)	65+	21.3	10	**0.0188***
Mexico City	Coyoacán	Chapter 5 (Mental and behavioural disorders)	35-44	22.2	10	**0.0143***
Mexico City	Coyoacán	Chapter 5 (Mental and behavioural disorders)	25-34	1.4	7	0.9849
Mexico City	Cuajimalpa de Morelos	Chapter 9 (Diseases of the circulatory system)	15-24	12.2	10	0.2706
Mexico City	Cuajimalpa de Morelos	Chapter 9 (Diseases of the circulatory system)	35-44	11.9	10	0.2898
Mexico City	Cuajimalpa de Morelos	Chapter 9 (Diseases of the circulatory system)	45-54	9.1	10	0.5184
Mexico City	Cuajimalpa de Morelos	Chapter 9 (Diseases of the circulatory system)	55-64	14.7	10	0.1425
Mexico City	Cuajimalpa de Morelos	Chapter 9 (Diseases of the circulatory system)	65+	15.0	10	0.1327
Mexico City	Cuajimalpa de Morelos	Chapter 9 (Diseases of the circulatory system)	25-34	6.6	10	0.7664
Mexico City	Cuajimalpa de Morelos	Chapter 9 (Diseases of the circulatory system)	0-4	4.4	6	0.6283
Mexico City	Cuajimalpa de Morelos	Chapter 4 (Endocrine, nutritional and metabolic diseases)	0-4	8.2	10	0.6121
Mexico City	Cuajimalpa de Morelos	Chapter 4 (Endocrine, nutritional and metabolic diseases)	25-34	9.2	9	0.4196
Mexico City	Cuajimalpa de Morelos	Chapter 4 (Endocrine, nutritional and metabolic diseases)	35-44	21.4	10	**0.0186***
Mexico City	Cuajimalpa de Morelos	Chapter 4 (Endocrine, nutritional and metabolic diseases)	45-54	10.6	10	0.3914
Mexico City	Cuajimalpa de Morelos	Chapter 4 (Endocrine, nutritional and metabolic diseases)	55-64	15.6	10	0.1125
Mexico City	Cuajimalpa de Morelos	Chapter 4 (Endocrine, nutritional and metabolic diseases)	65+	27.0	10	**0.0026***
Mexico City	Cuajimalpa de Morelos	Chapter 4 (Endocrine, nutritional and metabolic diseases)	15-24	5.8	8	0.6640
Mexico City	Cuajimalpa de Morelos	Chapter 6 (Diseases of the nervous system)	5-14	9.8	7	0.2030
Mexico City	Cuajimalpa de Morelos	Chapter 6 (Diseases of the nervous system)	65+	19.0	10	**0.0405***
Mexico City	Cuajimalpa de Morelos	Chapter 6 (Diseases of the nervous system)	0-4	9.8	10	0.4610
Mexico City	Cuajimalpa de Morelos	Chapter 6 (Diseases of the nervous system)	15-24	2.5	7	0.9243
Mexico City	Cuajimalpa de Morelos	Chapter 6 (Diseases of the nervous system)	25-34	6.3	5	0.2783
Mexico City	Cuajimalpa de Morelos	Chapter 6 (Diseases of the nervous system)	35-44	1.8	6	0.9375
Mexico City	Cuajimalpa de Morelos	Chapter 6 (Diseases of the nervous system)	55-64	1.9	10	0.9970
Mexico City	Cuajimalpa de Morelos	Chapter 6 (Diseases of the nervous system)	45-54	9.4	9	0.4014
Mexico City	Cuajimalpa de Morelos	Chapter 10 (Diseases of the respiratory system)	0-4	35.4	10	**0.0001***
Mexico City	Cuajimalpa de Morelos	Chapter 10 (Diseases of the respiratory system)	35-44	9.8	8	0.2829
Mexico City	Cuajimalpa de Morelos	Chapter 10 (Diseases of the respiratory system)	45-54	15.9	10	0.1016
Mexico City	Cuajimalpa de Morelos	Chapter 10 (Diseases of the respiratory system)	55-64	25.5	10	**0.0044***
Mexico City	Cuajimalpa de Morelos	Chapter 10 (Diseases of the respiratory system)	65+	12.0	10	0.2850
Mexico City	Cuajimalpa de Morelos	Chapter 10 (Diseases of the respiratory system)	25-34	13.4	7	0.0638
Mexico City	Cuajimalpa de Morelos	Chapter 10 (Diseases of the respiratory system)	15-24	10.0	6	0.1258
Mexico City	Cuajimalpa de Morelos	Chapter 5 (Mental and behavioural disorders)	25-34	2.6	5	0.7641
Mexico City	Cuajimalpa de Morelos	Chapter 5 (Mental and behavioural disorders)	55-64	9.2	8	0.3280
Mexico City	Cuajimalpa de Morelos	Chapter 5 (Mental and behavioural disorders)	35-44	21.3	10	**0.0189***
Mexico City	Cuajimalpa de Morelos	Chapter 5 (Mental and behavioural disorders)	65+	7.3	10	0.6977
Mexico City	Cuajimalpa de Morelos	Chapter 5 (Mental and behavioural disorders)	45-54	6.7	5	0.2465

Mexico City	Gustavo A. Madero	Chapter 9 (Diseases of the circulatory system)	0-4	18.4	10	0.0483
Mexico City	Gustavo A. Madero	Chapter 9 (Diseases of the circulatory system)	5-14	3.6	10	0.9632
Mexico City	Gustavo A. Madero	Chapter 9 (Diseases of the circulatory system)	15-24	13.4	10	0.2022
Mexico City	Gustavo A. Madero	Chapter 9 (Diseases of the circulatory system)	25-34	9.0	10	0.5315
Mexico City	Gustavo A. Madero	Chapter 9 (Diseases of the circulatory system)	35-44	10.9	10	0.3622
Mexico City	Gustavo A. Madero	Chapter 9 (Diseases of the circulatory system)	45-54	11.1	10	0.3489
Mexico City	Gustavo A. Madero	Chapter 9 (Diseases of the circulatory system)	55-64	8.1	10	0.6207
Mexico City	Gustavo A. Madero	Chapter 9 (Diseases of the circulatory system)	65+	4.1	10	0.9446
Mexico City	Gustavo A. Madero	Chapter 4 (Endocrine, nutritional and metabolic diseases)	0-4	21.6	10	**0.0173***
Mexico City	Gustavo A. Madero	Chapter 4 (Endocrine, nutritional and metabolic diseases)	5-14	8.1	10	0.6216
Mexico City	Gustavo A. Madero	Chapter 4 (Endocrine, nutritional and metabolic diseases)	15-24	9.0	10	0.5280
Mexico City	Gustavo A. Madero	Chapter 4 (Endocrine, nutritional and metabolic diseases)	25-34	13.7	10	0.1881
Mexico City	Gustavo A. Madero	Chapter 4 (Endocrine, nutritional and metabolic diseases)	35-44	28.6	10	**0.0015***
Mexico City	Gustavo A. Madero	Chapter 4 (Endocrine, nutritional and metabolic diseases)	45-54	10.0	10	0.4407
Mexico City	Gustavo A. Madero	Chapter 4 (Endocrine, nutritional and metabolic diseases)	55-64	5.1	10	0.8828
Mexico City	Gustavo A. Madero	Chapter 4 (Endocrine, nutritional and metabolic diseases)	65+	20.7	10	**0.0234***
Mexico City	Gustavo A. Madero	Chapter 6 (Diseases of the nervous system)	0-4	14.4	10	0.1540
Mexico City	Gustavo A. Madero	Chapter 6 (Diseases of the nervous system)	5-14	11.4	10	0.3240
Mexico City	Gustavo A. Madero	Chapter 6 (Diseases of the nervous system)	15-24	5.4	10	0.8660
Mexico City	Gustavo A. Madero	Chapter 6 (Diseases of the nervous system)	25-34	2.4	10	0.9924
Mexico City	Gustavo A. Madero	Chapter 6 (Diseases of the nervous system)	35-44	7.5	10	0.6805
Mexico City	Gustavo A. Madero	Chapter 6 (Diseases of the nervous system)	45-54	3.3	10	0.9729
Mexico City	Gustavo A. Madero	Chapter 6 (Diseases of the nervous system)	55-64	13.8	10	0.1838
Mexico City	Gustavo A. Madero	Chapter 6 (Diseases of the nervous system)	65+	7.6	10	0.6657
Mexico City	Gustavo A. Madero	Chapter 10 (Diseases of the respiratory system)	0-4	18.7	10	**0.0437***
Mexico City	Gustavo A. Madero	Chapter 10 (Diseases of the respiratory system)	5-14	13.6	10	0.1913
Mexico City	Gustavo A. Madero	Chapter 10 (Diseases of the respiratory system)	15-24	9.6	10	0.4740
Mexico City	Gustavo A. Madero	Chapter 10 (Diseases of the respiratory system)	25-34	25.0	10	**0.0054***
Mexico City	Gustavo A. Madero	Chapter 10 (Diseases of the respiratory system)	35-44	6.8	10	0.7486
Mexico City	Gustavo A. Madero	Chapter 10 (Diseases of the respiratory system)	45-54	10.2	10	0.4265
Mexico City	Gustavo A. Madero	Chapter 10 (Diseases of the respiratory system)	55-64	13.5	10	0.1957
Mexico City	Gustavo A. Madero	Chapter 10 (Diseases of the respiratory system)	65+	9.2	10	0.5163
Mexico City	Gustavo A. Madero	Chapter 5 (Mental and behavioural disorders)	15-24	2.2	8	0.9739
Mexico City	Gustavo A. Madero	Chapter 5 (Mental and behavioural disorders)	25-34	11.9	10	0.2926
Mexico City	Gustavo A. Madero	Chapter 5 (Mental and behavioural disorders)	35-44	8.4	10	0.5865
Mexico City	Gustavo A. Madero	Chapter 5 (Mental and behavioural disorders)	45-54	15.8	10	0.1070
Mexico City	Gustavo A. Madero	Chapter 5 (Mental and behavioural disorders)	55-64	17.7	10	0.0595
Mexico City	Gustavo A. Madero	Chapter 5 (Mental and behavioural disorders)	65+	5.2	10	0.8787
Mexico City	Iztacalco	Chapter 9 (Diseases of the circulatory system)	0-4	17.0	8	**0.0301***
Mexico City	Iztacalco	Chapter 9 (Diseases of the circulatory system)	25-34	10.1	10	0.4288
Mexico City	Iztacalco	Chapter 9 (Diseases of the circulatory system)	35-44	6.9	10	0.7325
Mexico City	Iztacalco	Chapter 9 (Diseases of the circulatory system)	45-54	5.2	10	0.8796
Mexico City	Iztacalco	Chapter 9 (Diseases of the circulatory system)	55-64	9.9	10	0.4452

Mexico City	Iztacalco	Chapter 9 (Diseases of the circulatory system)	65+	7.2	10	0.7062
Mexico City	Iztacalco	Chapter 9 (Diseases of the circulatory system)	15-24	8.1	10	0.6171
Mexico City	Iztacalco	Chapter 9 (Diseases of the circulatory system)	5-14	4.3	4	0.3695
Mexico City	Iztacalco	Chapter 4 (Endocrine, nutritional and metabolic diseases)	5-14	3.3	4	0.5107
Mexico City	Iztacalco	Chapter 4 (Endocrine, nutritional and metabolic diseases)	15-24	11.6	10	0.3094
Mexico City	Iztacalco	Chapter 4 (Endocrine, nutritional and metabolic diseases)	25-34	17.8	10	0.0591
Mexico City	Iztacalco	Chapter 4 (Endocrine, nutritional and metabolic diseases)	35-44	9.3	10	0.5066
Mexico City	Iztacalco	Chapter 4 (Endocrine, nutritional and metabolic diseases)	45-54	7.0	10	0.7242
Mexico City	Iztacalco	Chapter 4 (Endocrine, nutritional and metabolic diseases)	55-64	9.5	10	0.4810
Mexico City	Iztacalco	Chapter 4 (Endocrine, nutritional and metabolic diseases)	65+	12.2	10	0.2712
Mexico City	Iztacalco	Chapter 4 (Endocrine, nutritional and metabolic diseases)	0-4	17.9	10	0.0572
Mexico City	Iztacalco	Chapter 6 (Diseases of the nervous system)	0-4	1.9	10	0.9970
Mexico City	Iztacalco	Chapter 6 (Diseases of the nervous system)	5-14	10.8	10	0.3744
Mexico City	Iztacalco	Chapter 6 (Diseases of the nervous system)	15-24	8.3	10	0.5982
Mexico City	Iztacalco	Chapter 6 (Diseases of the nervous system)	25-34	7.5	10	0.6761
Mexico City	Iztacalco	Chapter 6 (Diseases of the nervous system)	35-44	17.5	10	0.0634
Mexico City	Iztacalco	Chapter 6 (Diseases of the nervous system)	45-54	4.8	10	0.9037
Mexico City	Iztacalco	Chapter 6 (Diseases of the nervous system)	55-64	13.9	10	0.1764
Mexico City	Iztacalco	Chapter 6 (Diseases of the nervous system)	65+	28.9	10	**0.0013***
Mexico City	Iztacalco	Chapter 10 (Diseases of the respiratory system)	0-4	9.4	10	0.4918
Mexico City	Iztacalco	Chapter 10 (Diseases of the respiratory system)	5-14	8.6	5	0.1255
Mexico City	Iztacalco	Chapter 10 (Diseases of the respiratory system)	25-34	7.6	10	0.6704
Mexico City	Iztacalco	Chapter 10 (Diseases of the respiratory system)	35-44	26.2	10	**0.0035***
Mexico City	Iztacalco	Chapter 10 (Diseases of the respiratory system)	45-54	18.1	10	0.0531
Mexico City	Iztacalco	Chapter 10 (Diseases of the respiratory system)	55-64	12.0	10	0.2842
Mexico City	Iztacalco	Chapter 10 (Diseases of the respiratory system)	65+	15.1	10	0.1286
Mexico City	Iztacalco	Chapter 10 (Diseases of the respiratory system)	15-24	9.5	9	0.3959
Mexico City	Iztacalco	Chapter 5 (Mental and behavioural disorders)	35-44	9.3	10	0.5077
Mexico City	Iztacalco	Chapter 5 (Mental and behavioural disorders)	45-54	5.7	10	0.8399
Mexico City	Iztacalco	Chapter 5 (Mental and behavioural disorders)	55-64	5.1	10	0.8854
Mexico City	Iztacalco	Chapter 5 (Mental and behavioural disorders)	65+	6.3	10	0.7910
Mexico City	Iztacalco	Chapter 5 (Mental and behavioural disorders)	25-34	2.3	7	0.9401
Mexico City	Iztapalapa	Chapter 9 (Diseases of the circulatory system)	0-4	19.9	10	**0.0298***
Mexico City	Iztapalapa	Chapter 9 (Diseases of the circulatory system)	5-14	8.6	10	0.5703
Mexico City	Iztapalapa	Chapter 9 (Diseases of the circulatory system)	15-24	6.6	10	0.7596
Mexico City	Iztapalapa	Chapter 9 (Diseases of the circulatory system)	25-34	20.7	10	**0.0234***
Mexico City	Iztapalapa	Chapter 9 (Diseases of the circulatory system)	35-44	23.4	10	**0.0092***
Mexico City	Iztapalapa	Chapter 9 (Diseases of the circulatory system)	45-54	11.0	10	0.3590
Mexico City	Iztapalapa	Chapter 9 (Diseases of the circulatory system)	55-64	11.0	10	0.3587
Mexico City	Iztapalapa	Chapter 9 (Diseases of the circulatory system)	65+	25.4	10	**0.0047***
Mexico City	Iztapalapa	Chapter 4 (Endocrine, nutritional and metabolic diseases)	0-4	7.0	10	0.7235
Mexico City	Iztapalapa	Chapter 4 (Endocrine, nutritional and metabolic diseases)	5-14	11.5	10	0.3174
Mexico City	Iztapalapa	Chapter 4 (Endocrine, nutritional and metabolic diseases)	15-24	8.7	10	0.5588

Mexico City	Iztapalapa	Chapter 4 (Endocrine, nutritional and metabolic diseases)	25-34	6.7	10	0.7530
Mexico City	Iztapalapa	Chapter 4 (Endocrine, nutritional and metabolic diseases)	35-44	14.4	10	0.1542
Mexico City	Iztapalapa	Chapter 4 (Endocrine, nutritional and metabolic diseases)	45-54	12.0	10	0.2821
Mexico City	Iztapalapa	Chapter 4 (Endocrine, nutritional and metabolic diseases)	55-64	5.8	10	0.8301
Mexico City	Iztapalapa	Chapter 4 (Endocrine, nutritional and metabolic diseases)	65+	15.2	10	0.1236
Mexico City	Iztapalapa	Chapter 6 (Diseases of the nervous system)	0-4	9.6	10	0.4794
Mexico City	Iztapalapa	Chapter 6 (Diseases of the nervous system)	5-14	6.3	10	0.7909
Mexico City	Iztapalapa	Chapter 6 (Diseases of the nervous system)	15-24	15.8	10	0.1061
Mexico City	Iztapalapa	Chapter 6 (Diseases of the nervous system)	25-34	6.4	10	0.7797
Mexico City	Iztapalapa	Chapter 6 (Diseases of the nervous system)	35-44	10.8	10	0.3725
Mexico City	Iztapalapa	Chapter 6 (Diseases of the nervous system)	45-54	26.8	10	**0.0028***
Mexico City	Iztapalapa	Chapter 6 (Diseases of the nervous system)	55-64	3.9	10	0.9504
Mexico City	Iztapalapa	Chapter 6 (Diseases of the nervous system)	65+	8.4	10	0.5880
Mexico City	Iztapalapa	Chapter 10 (Diseases of the respiratory system)	0-4	14.2	10	0.1662
Mexico City	Iztapalapa	Chapter 10 (Diseases of the respiratory system)	5-14	15.3	10	0.1212
Mexico City	Iztapalapa	Chapter 10 (Diseases of the respiratory system)	15-24	21.2	10	**0.0195***
Mexico City	Iztapalapa	Chapter 10 (Diseases of the respiratory system)	25-34	6.8	10	0.7467
Mexico City	Iztapalapa	Chapter 10 (Diseases of the respiratory system)	35-44	5.8	10	0.8300
Mexico City	Iztapalapa	Chapter 10 (Diseases of the respiratory system)	45-54	14.7	10	0.1439
Mexico City	Iztapalapa	Chapter 10 (Diseases of the respiratory system)	55-64	10.0	10	0.4425
Mexico City	Iztapalapa	Chapter 10 (Diseases of the respiratory system)	65+	34.0	10	**0.0002***
Mexico City	Iztapalapa	Chapter 5 (Mental and behavioural disorders)	15-24	20.1	10	**0.0285***
Mexico City	Iztapalapa	Chapter 5 (Mental and behavioural disorders)	25-34	6.8	10	0.7446
Mexico City	Iztapalapa	Chapter 5 (Mental and behavioural disorders)	35-44	12.2	10	0.2688
Mexico City	Iztapalapa	Chapter 5 (Mental and behavioural disorders)	45-54	8.0	10	0.6304
Mexico City	Iztapalapa	Chapter 5 (Mental and behavioural disorders)	55-64	13.2	10	0.2140
Mexico City	Iztapalapa	Chapter 5 (Mental and behavioural disorders)	65+	11.9	10	0.2911
Mexico City	La Magdalena Contreras	Chapter 9 (Diseases of the circulatory system)	0-4	1.8	5	0.8825
Mexico City	La Magdalena Contreras	Chapter 9 (Diseases of the circulatory system)	25-34	5.7	10	0.8386
Mexico City	La Magdalena Contreras	Chapter 9 (Diseases of the circulatory system)	35-44	9.8	10	0.4624
Mexico City	La Magdalena Contreras	Chapter 9 (Diseases of the circulatory system)	45-54	13.7	10	0.1890
Mexico City	La Magdalena Contreras	Chapter 9 (Diseases of the circulatory system)	55-64	13.6	10	0.1899
Mexico City	La Magdalena Contreras	Chapter 9 (Diseases of the circulatory system)	65+	14.4	10	0.1544
Mexico City	La Magdalena Contreras	Chapter 9 (Diseases of the circulatory system)	5-14	2.2	6	0.9031
Mexico City	La Magdalena Contreras	Chapter 9 (Diseases of the circulatory system)	15-24	14.3	10	0.1614
Mexico City	La Magdalena Contreras	Chapter 4 (Endocrine, nutritional and metabolic diseases)	0-4	2.2	4	0.6936
Mexico City	La Magdalena Contreras	Chapter 4 (Endocrine, nutritional and metabolic diseases)	15-24	5.9	6	0.4385
Mexico City	La Magdalena Contreras	Chapter 4 (Endocrine, nutritional and metabolic diseases)	25-34	20.7	10	**0.0234***
Mexico City	La Magdalena Contreras	Chapter 4 (Endocrine, nutritional and metabolic diseases)	35-44	3.6	10	0.9618
Mexico City	La Magdalena Contreras	Chapter 4 (Endocrine, nutritional and metabolic diseases)	45-54	6.6	10	0.7660
Mexico City	La Magdalena Contreras	Chapter 4 (Endocrine, nutritional and metabolic diseases)	55-64	11.3	10	0.3312
Mexico City	La Magdalena Contreras	Chapter 4 (Endocrine, nutritional and metabolic diseases)	65+	8.0	10	0.6325
Mexico City	La Magdalena Contreras	Chapter 6 (Diseases of the nervous system)	15-24	7.5	10	0.6772

Mexico City	La Magdalena Contreras	Chapter 6 (Diseases of the nervous system)	45-54	5.5	10	0.8540
Mexico City	La Magdalena Contreras	Chapter 6 (Diseases of the nervous system)	55-64	8.8	8	0.3567
Mexico City	La Magdalena Contreras	Chapter 6 (Diseases of the nervous system)	65+	13.3	10	0.2067
Mexico City	La Magdalena Contreras	Chapter 6 (Diseases of the nervous system)	0-4	3.9	8	0.8638
Mexico City	La Magdalena Contreras	Chapter 6 (Diseases of the nervous system)	5-14	12.1	6	0.0604
Mexico City	La Magdalena Contreras	Chapter 6 (Diseases of the nervous system)	25-34	15.5	6	**0.0170**
Mexico City	La Magdalena Contreras	Chapter 6 (Diseases of the nervous system)	35-44	6.8	10	0.7473
Mexico City	La Magdalena Contreras	Chapter 10 (Diseases of the respiratory system)	0-4	17.8	10	0.0584
Mexico City	La Magdalena Contreras	Chapter 10 (Diseases of the respiratory system)	35-44	7.7	10	0.6555
Mexico City	La Magdalena Contreras	Chapter 10 (Diseases of the respiratory system)	45-54	14.8	10	0.1380
Mexico City	La Magdalena Contreras	Chapter 10 (Diseases of the respiratory system)	55-64	5.8	10	0.8288
Mexico City	La Magdalena Contreras	Chapter 10 (Diseases of the respiratory system)	65+	14.4	10	0.1568
Mexico City	La Magdalena Contreras	Chapter 10 (Diseases of the respiratory system)	25-34	10.0	10	0.4409
Mexico City	La Magdalena Contreras	Chapter 10 (Diseases of the respiratory system)	15-24	5.2	5	0.3916
Mexico City	La Magdalena Contreras	Chapter 5 (Mental and behavioural disorders)	35-44	5.5	8	0.6987
Mexico City	La Magdalena Contreras	Chapter 5 (Mental and behavioural disorders)	55-64	17.6	9	**0.0406***
Mexico City	La Magdalena Contreras	Chapter 5 (Mental and behavioural disorders)	65+	8.0	10	0.6294
Mexico City	La Magdalena Contreras	Chapter 5 (Mental and behavioural disorders)	45-54	10.9	8	0.2059
Mexico City	Milpa Alta	Chapter 9 (Diseases of the circulatory system)	25-34	9.8	10	0.4539
Mexico City	Milpa Alta	Chapter 9 (Diseases of the circulatory system)	35-44	6.3	10	0.7854
Mexico City	Milpa Alta	Chapter 9 (Diseases of the circulatory system)	45-54	6.0	10	0.8164
Mexico City	Milpa Alta	Chapter 9 (Diseases of the circulatory system)	55-64	18.1	10	0.0525
Mexico City	Milpa Alta	Chapter 9 (Diseases of the circulatory system)	65+	35.6	10	**0.0001***
Mexico City	Milpa Alta	Chapter 9 (Diseases of the circulatory system)	15-24	8.2	5	0.1449
Mexico City	Milpa Alta	Chapter 4 (Endocrine, nutritional and metabolic diseases)	0-4	15.2	9	0.0867
Mexico City	Milpa Alta	Chapter 4 (Endocrine, nutritional and metabolic diseases)	25-34	16.5	10	0.0864
Mexico City	Milpa Alta	Chapter 4 (Endocrine, nutritional and metabolic diseases)	35-44	9.0	10	0.5349
Mexico City	Milpa Alta	Chapter 4 (Endocrine, nutritional and metabolic diseases)	45-54	15.4	10	0.1192
Mexico City	Milpa Alta	Chapter 4 (Endocrine, nutritional and metabolic diseases)	55-64	14.6	10	0.1468
Mexico City	Milpa Alta	Chapter 4 (Endocrine, nutritional and metabolic diseases)	65+	10.6	10	0.3877
Mexico City	Milpa Alta	Chapter 6 (Diseases of the nervous system)	65+	15.5	10	0.1157
Mexico City	Milpa Alta	Chapter 6 (Diseases of the nervous system)	5-14	1.9	10	0.9969
Mexico City	Milpa Alta	Chapter 6 (Diseases of the nervous system)	15-24	5.7	7	0.5757
Mexico City	Milpa Alta	Chapter 6 (Diseases of the nervous system)	0-4	3.7	6	0.7196
Mexico City	Milpa Alta	Chapter 6 (Diseases of the nervous system)	25-34	7.1	6	0.3133
Mexico City	Milpa Alta	Chapter 6 (Diseases of the nervous system)	55-64	3.8	6	0.7091
Mexico City	Milpa Alta	Chapter 6 (Diseases of the nervous system)	45-54	10.0	7	0.1871
Mexico City	Milpa Alta	Chapter 6 (Diseases of the nervous system)	35-44	11.1	6	0.0849
Mexico City	Milpa Alta	Chapter 10 (Diseases of the respiratory system)	0-4	8.3	10	0.5950
Mexico City	Milpa Alta	Chapter 10 (Diseases of the respiratory system)	45-54	9.7	10	0.4684
Mexico City	Milpa Alta	Chapter 10 (Diseases of the respiratory system)	55-64	16.1	10	0.0959
Mexico City	Milpa Alta	Chapter 10 (Diseases of the respiratory system)	65+	15.8	10	0.1064
Mexico City	Milpa Alta	Chapter 10 (Diseases of the respiratory system)	15-24	4.1	6	0.6594

Mexico City	Milpa Alta	Chapter 10 (Diseases of the respiratory system)	35-44	6.2	10	0.7963
Mexico City	Milpa Alta	Chapter 10 (Diseases of the respiratory system)	25-34	4.5	4	0.3411
Mexico City	Milpa Alta	Chapter 5 (Mental and behavioural disorders)	45-54	4.9	10	0.9003
Mexico City	Milpa Alta	Chapter 5 (Mental and behavioural disorders)	65+	11.3	10	0.3344
Mexico City	Milpa Alta	Chapter 5 (Mental and behavioural disorders)	25-34	4.3	5	0.5132
Mexico City	Milpa Alta	Chapter 5 (Mental and behavioural disorders)	35-44	17.6	8	**0.0244***
Mexico City	Milpa Alta	Chapter 5 (Mental and behavioural disorders)	55-64	8.4	8	0.3995
Mexico City	Álvaro Obregón	Chapter 9 (Diseases of the circulatory system)	0-4	13.2	10	0.2136
Mexico City	Álvaro Obregón	Chapter 9 (Diseases of the circulatory system)	5-14	5.5	6	0.4852
Mexico City	Álvaro Obregón	Chapter 9 (Diseases of the circulatory system)	15-24	11.0	10	0.3606
Mexico City	Álvaro Obregón	Chapter 9 (Diseases of the circulatory system)	25-34	14.3	10	0.1584
Mexico City	Álvaro Obregón	Chapter 9 (Diseases of the circulatory system)	35-44	13.0	10	0.2227
Mexico City	Álvaro Obregón	Chapter 9 (Diseases of the circulatory system)	45-54	10.5	10	0.3979
Mexico City	Álvaro Obregón	Chapter 9 (Diseases of the circulatory system)	55-64	16.4	10	0.0890
Mexico City	Álvaro Obregón	Chapter 9 (Diseases of the circulatory system)	65+	11.5	10	0.3183
Mexico City	Álvaro Obregón	Chapter 4 (Endocrine, nutritional and metabolic diseases)	0-4	11.3	10	0.3365
Mexico City	Álvaro Obregón	Chapter 4 (Endocrine, nutritional and metabolic diseases)	5-14	2.2	4	0.6988
Mexico City	Álvaro Obregón	Chapter 4 (Endocrine, nutritional and metabolic diseases)	25-34	9.9	10	0.4528
Mexico City	Álvaro Obregón	Chapter 4 (Endocrine, nutritional and metabolic diseases)	35-44	17.2	10	0.0706
Mexico City	Álvaro Obregón	Chapter 4 (Endocrine, nutritional and metabolic diseases)	45-54	20.3	10	**0.0267***
Mexico City	Álvaro Obregón	Chapter 4 (Endocrine, nutritional and metabolic diseases)	55-64	19.1	10	**0.0388***
Mexico City	Álvaro Obregón	Chapter 4 (Endocrine, nutritional and metabolic diseases)	65+	29.0	10	**0.0013***
Mexico City	Álvaro Obregón	Chapter 4 (Endocrine, nutritional and metabolic diseases)	15-24	18.9	10	**0.0413***
Mexico City	Álvaro Obregón	Chapter 6 (Diseases of the nervous system)	0-4	14.1	10	0.1666
Mexico City	Álvaro Obregón	Chapter 6 (Diseases of the nervous system)	5-14	11.8	10	0.3020
Mexico City	Álvaro Obregón	Chapter 6 (Diseases of the nervous system)	15-24	5.9	10	0.8266
Mexico City	Álvaro Obregón	Chapter 6 (Diseases of the nervous system)	25-34	9.3	10	0.5058
Mexico City	Álvaro Obregón	Chapter 6 (Diseases of the nervous system)	35-44	16.3	10	0.0922
Mexico City	Álvaro Obregón	Chapter 6 (Diseases of the nervous system)	45-54	12.0	10	0.2853
Mexico City	Álvaro Obregón	Chapter 6 (Diseases of the nervous system)	55-64	8.9	10	0.5374
Mexico City	Álvaro Obregón	Chapter 6 (Diseases of the nervous system)	65+	8.2	10	0.6060
Mexico City	Álvaro Obregón	Chapter 10 (Diseases of the respiratory system)	0-4	11.5	10	0.3224
Mexico City	Álvaro Obregón	Chapter 10 (Diseases of the respiratory system)	5-14	6.4	9	0.6983
Mexico City	Álvaro Obregón	Chapter 10 (Diseases of the respiratory system)	15-24	6.3	10	0.7919
Mexico City	Álvaro Obregón	Chapter 10 (Diseases of the respiratory system)	25-34	21.0	10	**0.0209***
Mexico City	Álvaro Obregón	Chapter 10 (Diseases of the respiratory system)	35-44	9.3	10	0.5021
Mexico City	Álvaro Obregón	Chapter 10 (Diseases of the respiratory system)	45-54	19.1	10	**0.0385***
Mexico City	Álvaro Obregón	Chapter 10 (Diseases of the respiratory system)	55-64	9.3	10	0.5020
Mexico City	Álvaro Obregón	Chapter 10 (Diseases of the respiratory system)	65+	24.0	10	**0.0076***
Mexico City	Álvaro Obregón	Chapter 5 (Mental and behavioural disorders)	25-34	5.6	7	0.5818
Mexico City	Álvaro Obregón	Chapter 5 (Mental and behavioural disorders)	45-54	9.5	10	0.4824
Mexico City	Álvaro Obregón	Chapter 5 (Mental and behavioural disorders)	55-64	10.4	10	0.4051
Mexico City	Álvaro Obregón	Chapter 5 (Mental and behavioural disorders)	65+	10.0	10	0.4406

Mexico City	Álvaro Obregón	Chapter 5 (Mental and behavioural disorders)	35-44	4.8	10	0.9010
Mexico City	Álvaro Obregón	Chapter 5 (Mental and behavioural disorders)	15-24	12.7	4	**0.0126***
Mexico City	Tláhuac	Chapter 9 (Diseases of the circulatory system)	25-34	12.0	10	0.2828
Mexico City	Tláhuac	Chapter 9 (Diseases of the circulatory system)	35-44	20.8	10	**0.0229***
Mexico City	Tláhuac	Chapter 9 (Diseases of the circulatory system)	45-54	8.4	10	0.5923
Mexico City	Tláhuac	Chapter 9 (Diseases of the circulatory system)	55-64	5.8	10	0.8334
Mexico City	Tláhuac	Chapter 9 (Diseases of the circulatory system)	65+	8.8	10	0.5525
Mexico City	Tláhuac	Chapter 9 (Diseases of the circulatory system)	0-4	7.2	7	0.4118
Mexico City	Tláhuac	Chapter 9 (Diseases of the circulatory system)	15-24	9.3	10	0.5012
Mexico City	Tláhuac	Chapter 9 (Diseases of the circulatory system)	5-14	6.5	5	0.2587
Mexico City	Tláhuac	Chapter 4 (Endocrine, nutritional and metabolic diseases)	5-14	4.5	4	0.3398
Mexico City	Tláhuac	Chapter 4 (Endocrine, nutritional and metabolic diseases)	25-34	14.8	10	0.1393
Mexico City	Tláhuac	Chapter 4 (Endocrine, nutritional and metabolic diseases)	35-44	8.7	10	0.5613
Mexico City	Tláhuac	Chapter 4 (Endocrine, nutritional and metabolic diseases)	45-54	20.7	10	**0.0233***
Mexico City	Tláhuac	Chapter 4 (Endocrine, nutritional and metabolic diseases)	55-64	9.7	10	0.4649
Mexico City	Tláhuac	Chapter 4 (Endocrine, nutritional and metabolic diseases)	65+	6.3	10	0.7879
Mexico City	Tláhuac	Chapter 4 (Endocrine, nutritional and metabolic diseases)	0-4	3.5	7	0.8383
Mexico City	Tláhuac	Chapter 4 (Endocrine, nutritional and metabolic diseases)	15-24	2.0	10	0.9963
Mexico City	Tláhuac	Chapter 6 (Diseases of the nervous system)	25-34	13.7	10	0.1891
Mexico City	Tláhuac	Chapter 6 (Diseases of the nervous system)	35-44	11.7	10	0.3033
Mexico City	Tláhuac	Chapter 6 (Diseases of the nervous system)	55-64	8.8	10	0.5552
Mexico City	Tláhuac	Chapter 6 (Diseases of the nervous system)	65+	14.1	10	0.1663
Mexico City	Tláhuac	Chapter 6 (Diseases of the nervous system)	0-4	9.6	10	0.4778
Mexico City	Tláhuac	Chapter 6 (Diseases of the nervous system)	45-54	17.0	10	0.0735
Mexico City	Tláhuac	Chapter 6 (Diseases of the nervous system)	15-24	10.7	10	0.3818
Mexico City	Tláhuac	Chapter 6 (Diseases of the nervous system)	5-14	6.2	9	0.7161
Mexico City	Tláhuac	Chapter 10 (Diseases of the respiratory system)	0-4	9.1	10	0.5262
Mexico City	Tláhuac	Chapter 10 (Diseases of the respiratory system)	5-14	5.2	5	0.3975
Mexico City	Tláhuac	Chapter 10 (Diseases of the respiratory system)	15-24	9.8	9	0.3677
Mexico City	Tláhuac	Chapter 10 (Diseases of the respiratory system)	25-34	9.0	10	0.5276
Mexico City	Tláhuac	Chapter 10 (Diseases of the respiratory system)	35-44	7.6	10	0.6726
Mexico City	Tláhuac	Chapter 10 (Diseases of the respiratory system)	45-54	10.9	10	0.3651
Mexico City	Tláhuac	Chapter 10 (Diseases of the respiratory system)	55-64	12.9	10	0.2315
Mexico City	Tláhuac	Chapter 10 (Diseases of the respiratory system)	65+	9.5	10	0.4878
Mexico City	Tláhuac	Chapter 5 (Mental and behavioural disorders)	25-34	2.2	5	0.8184
Mexico City	Tláhuac	Chapter 5 (Mental and behavioural disorders)	45-54	18.2	9	**0.0334***
Mexico City	Tláhuac	Chapter 5 (Mental and behavioural disorders)	65+	6.4	10	0.7826
Mexico City	Tláhuac	Chapter 5 (Mental and behavioural disorders)	35-44	11.7	10	0.3078
Mexico City	Tláhuac	Chapter 5 (Mental and behavioural disorders)	55-64	7.4	8	0.4983
Mexico City	Tlalpan	Chapter 9 (Diseases of the circulatory system)	0-4	7.1	10	0.7122
Mexico City	Tlalpan	Chapter 9 (Diseases of the circulatory system)	15-24	19.2	10	**0.0376***
Mexico City	Tlalpan	Chapter 9 (Diseases of the circulatory system)	25-34	7.3	10	0.6943
Mexico City	Tlalpan	Chapter 9 (Diseases of the circulatory system)	35-44	5.1	10	0.8840

Mexico City	Tlalpan	Chapter 9 (Diseases of the circulatory system)	45-54	6.7	10	0.7548
Mexico City	Tlalpan	Chapter 9 (Diseases of the circulatory system)	55-64	6.9	10	0.7388
Mexico City	Tlalpan	Chapter 9 (Diseases of the circulatory system)	65+	8.6	10	0.5748
Mexico City	Tlalpan	Chapter 9 (Diseases of the circulatory system)	5-14	12.9	7	0.0735
Mexico City	Tlalpan	Chapter 4 (Endocrine, nutritional and metabolic diseases)	0-4	11.1	10	0.3494
Mexico City	Tlalpan	Chapter 4 (Endocrine, nutritional and metabolic diseases)	15-24	5.8	10	0.8316
Mexico City	Tlalpan	Chapter 4 (Endocrine, nutritional and metabolic diseases)	25-34	16.2	10	0.0945
Mexico City	Tlalpan	Chapter 4 (Endocrine, nutritional and metabolic diseases)	35-44	5.3	10	0.8716
Mexico City	Tlalpan	Chapter 4 (Endocrine, nutritional and metabolic diseases)	45-54	9.5	10	0.4847
Mexico City	Tlalpan	Chapter 4 (Endocrine, nutritional and metabolic diseases)	55-64	12.0	10	0.2836
Mexico City	Tlalpan	Chapter 4 (Endocrine, nutritional and metabolic diseases)	65+	7.1	10	0.7126
Mexico City	Tlalpan	Chapter 4 (Endocrine, nutritional and metabolic diseases)	5-14	8.8	5	0.1159
Mexico City	Tlalpan	Chapter 6 (Diseases of the nervous system)	0-4	12.0	10	0.2863
Mexico City	Tlalpan	Chapter 6 (Diseases of the nervous system)	5-14	8.9	10	0.5459
Mexico City	Tlalpan	Chapter 6 (Diseases of the nervous system)	15-24	5.4	10	0.8657
Mexico City	Tlalpan	Chapter 6 (Diseases of the nervous system)	25-34	8.2	10	0.6116
Mexico City	Tlalpan	Chapter 6 (Diseases of the nervous system)	35-44	8.3	10	0.6030
Mexico City	Tlalpan	Chapter 6 (Diseases of the nervous system)	45-54	16.6	10	0.0828
Mexico City	Tlalpan	Chapter 6 (Diseases of the nervous system)	55-64	15.1	10	0.1285
Mexico City	Tlalpan	Chapter 6 (Diseases of the nervous system)	65+	13.3	10	0.2050
Mexico City	Tlalpan	Chapter 10 (Diseases of the respiratory system)	0-4	10.0	10	0.4405
Mexico City	Tlalpan	Chapter 10 (Diseases of the respiratory system)	15-24	3.8	10	0.9544
Mexico City	Tlalpan	Chapter 10 (Diseases of the respiratory system)	25-34	11.3	10	0.3346
Mexico City	Tlalpan	Chapter 10 (Diseases of the respiratory system)	35-44	9.6	10	0.4807
Mexico City	Tlalpan	Chapter 10 (Diseases of the respiratory system)	45-54	4.5	10	0.9225
Mexico City	Tlalpan	Chapter 10 (Diseases of the respiratory system)	55-64	22.0	10	**0.0150***
Mexico City	Tlalpan	Chapter 10 (Diseases of the respiratory system)	65+	6.8	10	0.7464
Mexico City	Tlalpan	Chapter 10 (Diseases of the respiratory system)	5-14	6.1	7	0.5258
Mexico City	Tlalpan	Chapter 5 (Mental and behavioural disorders)	25-34	11.1	10	0.3477
Mexico City	Tlalpan	Chapter 5 (Mental and behavioural disorders)	35-44	7.4	10	0.6826
Mexico City	Tlalpan	Chapter 5 (Mental and behavioural disorders)	45-54	8.8	10	0.5551
Mexico City	Tlalpan	Chapter 5 (Mental and behavioural disorders)	55-64	13.5	10	0.1965
Mexico City	Tlalpan	Chapter 5 (Mental and behavioural disorders)	65+	19.2	10	**0.0377***
Mexico City	Xochimilco	Chapter 9 (Diseases of the circulatory system)	0-4	4.1	9	0.9057
Mexico City	Xochimilco	Chapter 9 (Diseases of the circulatory system)	25-34	10.5	10	0.3963
Mexico City	Xochimilco	Chapter 9 (Diseases of the circulatory system)	35-44	7.0	10	0.7277
Mexico City	Xochimilco	Chapter 9 (Diseases of the circulatory system)	45-54	18.7	10	**0.0436***
Mexico City	Xochimilco	Chapter 9 (Diseases of the circulatory system)	55-64	10.1	10	0.4289
Mexico City	Xochimilco	Chapter 9 (Diseases of the circulatory system)	65+	10.8	10	0.3712
Mexico City	Xochimilco	Chapter 9 (Diseases of the circulatory system)	5-14	4.1	4	0.3878
Mexico City	Xochimilco	Chapter 9 (Diseases of the circulatory system)	15-24	15.8	10	0.1058
Mexico City	Xochimilco	Chapter 4 (Endocrine, nutritional and metabolic diseases)	25-34	19.7	10	**0.0325***
Mexico City	Xochimilco	Chapter 4 (Endocrine, nutritional and metabolic diseases)	35-44	9.8	10	0.4624

Mexico City	Xochimilco	Chapter 4 (Endocrine, nutritional and metabolic diseases)	45-54	9.6	10	0.4801
Mexico City	Xochimilco	Chapter 4 (Endocrine, nutritional and metabolic diseases)	55-64	7.1	10	0.7149
Mexico City	Xochimilco	Chapter 4 (Endocrine, nutritional and metabolic diseases)	65+	10.5	10	0.3966
Mexico City	Xochimilco	Chapter 4 (Endocrine, nutritional and metabolic diseases)	0-4	10.7	10	0.3830
Mexico City	Xochimilco	Chapter 4 (Endocrine, nutritional and metabolic diseases)	15-24	9.8	9	0.3668
Mexico City	Xochimilco	Chapter 6 (Diseases of the nervous system)	0-4	17.4	10	0.0661
Mexico City	Xochimilco	Chapter 6 (Diseases of the nervous system)	25-34	15.5	10	0.1151
Mexico City	Xochimilco	Chapter 6 (Diseases of the nervous system)	55-64	20.8	10	**0.0227***
Mexico City	Xochimilco	Chapter 6 (Diseases of the nervous system)	65+	17.4	10	0.0657
Mexico City	Xochimilco	Chapter 6 (Diseases of the nervous system)	5-14	14.4	10	0.1538
Mexico City	Xochimilco	Chapter 6 (Diseases of the nervous system)	15-24	20.6	10	**0.0244***
Mexico City	Xochimilco	Chapter 6 (Diseases of the nervous system)	35-44	13.4	10	0.2025
Mexico City	Xochimilco	Chapter 6 (Diseases of the nervous system)	45-54	12.2	10	0.2741
Mexico City	Xochimilco	Chapter 10 (Diseases of the respiratory system)	0-4	7.8	10	0.6522
Mexico City	Xochimilco	Chapter 10 (Diseases of the respiratory system)	25-34	11.8	10	0.3018
Mexico City	Xochimilco	Chapter 10 (Diseases of the respiratory system)	35-44	6.3	10	0.7891
Mexico City	Xochimilco	Chapter 10 (Diseases of the respiratory system)	45-54	17.5	10	0.0632
Mexico City	Xochimilco	Chapter 10 (Diseases of the respiratory system)	55-64	6.5	10	0.7690
Mexico City	Xochimilco	Chapter 10 (Diseases of the respiratory system)	65+	9.2	10	0.5137
Mexico City	Xochimilco	Chapter 10 (Diseases of the respiratory system)	5-14	9.9	7	0.1965
Mexico City	Xochimilco	Chapter 10 (Diseases of the respiratory system)	15-24	7.8	10	0.6462
Mexico City	Xochimilco	Chapter 5 (Mental and behavioural disorders)	25-34	11.8	10	0.2953
Mexico City	Xochimilco	Chapter 5 (Mental and behavioural disorders)	35-44	2.3	10	0.9933
Mexico City	Xochimilco	Chapter 5 (Mental and behavioural disorders)	45-54	15.8	10	0.1067
Mexico City	Xochimilco	Chapter 5 (Mental and behavioural disorders)	55-64	16.0	10	0.1001
Mexico City	Xochimilco	Chapter 5 (Mental and behavioural disorders)	65+	7.7	10	0.6571
Mexico City	Benito Juárez	Chapter 9 (Diseases of the circulatory system)	0-4	7.6	6	0.2694
Mexico City	Benito Juárez	Chapter 9 (Diseases of the circulatory system)	15-24	12.9	10	0.2313
Mexico City	Benito Juárez	Chapter 9 (Diseases of the circulatory system)	35-44	14.7	10	0.1434
Mexico City	Benito Juárez	Chapter 9 (Diseases of the circulatory system)	45-54	16.7	10	0.0812
Mexico City	Benito Juárez	Chapter 9 (Diseases of the circulatory system)	55-64	10.2	10	0.4220
Mexico City	Benito Juárez	Chapter 9 (Diseases of the circulatory system)	65+	7.9	10	0.6368
Mexico City	Benito Juárez	Chapter 9 (Diseases of the circulatory system)	25-34	5.2	10	0.8748
Mexico City	Benito Juárez	Chapter 4 (Endocrine, nutritional and metabolic diseases)	25-34	21.0	10	**0.0210***
Mexico City	Benito Juárez	Chapter 4 (Endocrine, nutritional and metabolic diseases)	35-44	4.5	10	0.9231
Mexico City	Benito Juárez	Chapter 4 (Endocrine, nutritional and metabolic diseases)	45-54	18.1	10	0.0528
Mexico City	Benito Juárez	Chapter 4 (Endocrine, nutritional and metabolic diseases)	55-64	21.7	10	**0.0168***
Mexico City	Benito Juárez	Chapter 4 (Endocrine, nutritional and metabolic diseases)	65+	7.3	10	0.6997
Mexico City	Benito Juárez	Chapter 4 (Endocrine, nutritional and metabolic diseases)	15-24	9.8	5	0.0819
Mexico City	Benito Juárez	Chapter 6 (Diseases of the nervous system)	5-14	16.4	9	0.0586
Mexico City	Benito Juárez	Chapter 6 (Diseases of the nervous system)	15-24	9.2	10	0.5141
Mexico City	Benito Juárez	Chapter 6 (Diseases of the nervous system)	25-34	6.0	10	0.8163
Mexico City	Benito Juárez	Chapter 6 (Diseases of the nervous system)	35-44	7.4	10	0.6878

Mexico City	Benito Juárez	Chapter 6 (Diseases of the nervous system)	55-64	8.9	10	0.5413
Mexico City	Benito Juárez	Chapter 6 (Diseases of the nervous system)	65+	10.2	10	0.4206
Mexico City	Benito Juárez	Chapter 6 (Diseases of the nervous system)	0-4	12.5	10	0.2559
Mexico City	Benito Juárez	Chapter 6 (Diseases of the nervous system)	45-54	9.1	10	0.5229
Mexico City	Benito Juárez	Chapter 10 (Diseases of the respiratory system)	0-4	8.5	10	0.5828
Mexico City	Benito Juárez	Chapter 10 (Diseases of the respiratory system)	15-24	5.8	10	0.8302
Mexico City	Benito Juárez	Chapter 10 (Diseases of the respiratory system)	35-44	4.6	10	0.9152
Mexico City	Benito Juárez	Chapter 10 (Diseases of the respiratory system)	45-54	12.5	10	0.2534
Mexico City	Benito Juárez	Chapter 10 (Diseases of the respiratory system)	55-64	5.2	10	0.8768
Mexico City	Benito Juárez	Chapter 10 (Diseases of the respiratory system)	65+	31.1	10	**0.0006***
Mexico City	Benito Juárez	Chapter 10 (Diseases of the respiratory system)	25-34	11.7	10	0.3040
Mexico City	Benito Juárez	Chapter 10 (Diseases of the respiratory system)	5-14	4.9	5	0.4259
Mexico City	Benito Juárez	Chapter 5 (Mental and behavioural disorders)	35-44	10.5	8	0.2303
Mexico City	Benito Juárez	Chapter 5 (Mental and behavioural disorders)	45-54	4.9	10	0.8952
Mexico City	Benito Juárez	Chapter 5 (Mental and behavioural disorders)	55-64	13.5	10	0.1976
Mexico City	Benito Juárez	Chapter 5 (Mental and behavioural disorders)	65+	10.8	10	0.3764
Mexico City	Cuauhtémoc	Chapter 9 (Diseases of the circulatory system)	0-4	8.9	10	0.5443
Mexico City	Cuauhtémoc	Chapter 9 (Diseases of the circulatory system)	15-24	10.0	10	0.4442
Mexico City	Cuauhtémoc	Chapter 9 (Diseases of the circulatory system)	25-34	7.6	10	0.6647
Mexico City	Cuauhtémoc	Chapter 9 (Diseases of the circulatory system)	35-44	7.7	10	0.6543
Mexico City	Cuauhtémoc	Chapter 9 (Diseases of the circulatory system)	45-54	12.3	10	0.2658
Mexico City	Cuauhtémoc	Chapter 9 (Diseases of the circulatory system)	55-64	5.3	10	0.8680
Mexico City	Cuauhtémoc	Chapter 9 (Diseases of the circulatory system)	65+	8.5	10	0.5771
Mexico City	Cuauhtémoc	Chapter 9 (Diseases of the circulatory system)	5-14	8.7	10	0.5626
Mexico City	Cuauhtémoc	Chapter 4 (Endocrine, nutritional and metabolic diseases)	5-14	2.9	6	0.8197
Mexico City	Cuauhtémoc	Chapter 4 (Endocrine, nutritional and metabolic diseases)	15-24	12.0	10	0.2864
Mexico City	Cuauhtémoc	Chapter 4 (Endocrine, nutritional and metabolic diseases)	25-34	8.2	10	0.6125
Mexico City	Cuauhtémoc	Chapter 4 (Endocrine, nutritional and metabolic diseases)	35-44	7.1	10	0.7189
Mexico City	Cuauhtémoc	Chapter 4 (Endocrine, nutritional and metabolic diseases)	45-54	17.0	10	0.0735
Mexico City	Cuauhtémoc	Chapter 4 (Endocrine, nutritional and metabolic diseases)	55-64	7.8	10	0.6452
Mexico City	Cuauhtémoc	Chapter 4 (Endocrine, nutritional and metabolic diseases)	65+	22.0	10	**0.0153***
Mexico City	Cuauhtémoc	Chapter 4 (Endocrine, nutritional and metabolic diseases)	0-4	7.0	8	0.5328
Mexico City	Cuauhtémoc	Chapter 6 (Diseases of the nervous system)	0-4	8.5	10	0.5837
Mexico City	Cuauhtémoc	Chapter 6 (Diseases of the nervous system)	5-14	5.8	10	0.8289
Mexico City	Cuauhtémoc	Chapter 6 (Diseases of the nervous system)	15-24	29.9	10	**0.0009***
Mexico City	Cuauhtémoc	Chapter 6 (Diseases of the nervous system)	25-34	13.1	10	0.2180
Mexico City	Cuauhtémoc	Chapter 6 (Diseases of the nervous system)	35-44	35.8	10	**0.0001***
Mexico City	Cuauhtémoc	Chapter 6 (Diseases of the nervous system)	45-54	11.0	10	0.3577
Mexico City	Cuauhtémoc	Chapter 6 (Diseases of the nervous system)	55-64	15.8	10	0.1041
Mexico City	Cuauhtémoc	Chapter 6 (Diseases of the nervous system)	65+	18.6	10	**0.0451***
Mexico City	Cuauhtémoc	Chapter 10 (Diseases of the respiratory system)	0-4	16.5	10	0.0853
Mexico City	Cuauhtémoc	Chapter 10 (Diseases of the respiratory system)	15-24	13.7	10	0.1895
Mexico City	Cuauhtémoc	Chapter 10 (Diseases of the respiratory system)	25-34	7.1	10	0.7176

Mexico City	Cuauhtémoc	Chapter 10 (Diseases of the respiratory system)	35-44	4.2	10	0.9395
Mexico City	Cuauhtémoc	Chapter 10 (Diseases of the respiratory system)	45-54	8.7	10	0.5579
Mexico City	Cuauhtémoc	Chapter 10 (Diseases of the respiratory system)	55-64	10.3	10	0.4140
Mexico City	Cuauhtémoc	Chapter 10 (Diseases of the respiratory system)	65+	21.9	10	**0.0158***
Mexico City	Cuauhtémoc	Chapter 10 (Diseases of the respiratory system)	5-14	17.5	9	**0.0421***
Mexico City	Cuauhtémoc	Chapter 5 (Mental and behavioural disorders)	25-34	32.0	10	**0.0004***
Mexico City	Cuauhtémoc	Chapter 5 (Mental and behavioural disorders)	35-44	11.2	10	0.3415
Mexico City	Cuauhtémoc	Chapter 5 (Mental and behavioural disorders)	45-54	10.8	10	0.3713
Mexico City	Cuauhtémoc	Chapter 5 (Mental and behavioural disorders)	55-64	11.8	10	0.3017
Mexico City	Cuauhtémoc	Chapter 5 (Mental and behavioural disorders)	65+	22.4	10	**0.0132***
Mexico City	Cuauhtémoc	Chapter 5 (Mental and behavioural disorders)	15-24	3.4	4	0.5005
Mexico City	Miguel Hidalgo	Chapter 9 (Diseases of the circulatory system)	0-4	7.1	10	0.7125
Mexico City	Miguel Hidalgo	Chapter 9 (Diseases of the circulatory system)	15-24	9.3	10	0.5079
Mexico City	Miguel Hidalgo	Chapter 9 (Diseases of the circulatory system)	25-34	14.8	10	0.1379
Mexico City	Miguel Hidalgo	Chapter 9 (Diseases of the circulatory system)	35-44	11.3	10	0.3369
Mexico City	Miguel Hidalgo	Chapter 9 (Diseases of the circulatory system)	45-54	8.3	10	0.5996
Mexico City	Miguel Hidalgo	Chapter 9 (Diseases of the circulatory system)	55-64	13.3	10	0.2096
Mexico City	Miguel Hidalgo	Chapter 9 (Diseases of the circulatory system)	65+	13.8	10	0.1824
Mexico City	Miguel Hidalgo	Chapter 9 (Diseases of the circulatory system)	5-14	5.8	5	0.3236
Mexico City	Miguel Hidalgo	Chapter 4 (Endocrine, nutritional and metabolic diseases)	0-4	18.5	9	**0.0303***
Mexico City	Miguel Hidalgo	Chapter 4 (Endocrine, nutritional and metabolic diseases)	5-14	3.8	4	0.4320
Mexico City	Miguel Hidalgo	Chapter 4 (Endocrine, nutritional and metabolic diseases)	25-34	16.0	10	0.1001
Mexico City	Miguel Hidalgo	Chapter 4 (Endocrine, nutritional and metabolic diseases)	35-44	32.3	10	**0.0004***
Mexico City	Miguel Hidalgo	Chapter 4 (Endocrine, nutritional and metabolic diseases)	45-54	7.5	10	0.6801
Mexico City	Miguel Hidalgo	Chapter 4 (Endocrine, nutritional and metabolic diseases)	55-64	6.5	10	0.7718
Mexico City	Miguel Hidalgo	Chapter 4 (Endocrine, nutritional and metabolic diseases)	65+	13.7	10	0.1874
Mexico City	Miguel Hidalgo	Chapter 4 (Endocrine, nutritional and metabolic diseases)	15-24	14.7	9	0.0985
Mexico City	Miguel Hidalgo	Chapter 6 (Diseases of the nervous system)	0-4	9.2	10	0.5150
Mexico City	Miguel Hidalgo	Chapter 6 (Diseases of the nervous system)	15-24	2.1	10	0.9952
Mexico City	Miguel Hidalgo	Chapter 6 (Diseases of the nervous system)	25-34	14.3	10	0.1591
Mexico City	Miguel Hidalgo	Chapter 6 (Diseases of the nervous system)	45-54	13.0	10	0.2220
Mexico City	Miguel Hidalgo	Chapter 6 (Diseases of the nervous system)	55-64	5.3	10	0.8706
Mexico City	Miguel Hidalgo	Chapter 6 (Diseases of the nervous system)	65+	8.6	10	0.5665
Mexico City	Miguel Hidalgo	Chapter 6 (Diseases of the nervous system)	5-14	12.1	10	0.2802
Mexico City	Miguel Hidalgo	Chapter 6 (Diseases of the nervous system)	35-44	6.2	10	0.7970
Mexico City	Miguel Hidalgo	Chapter 10 (Diseases of the respiratory system)	0-4	3.1	10	0.9778
Mexico City	Miguel Hidalgo	Chapter 10 (Diseases of the respiratory system)	35-44	6.8	10	0.7416
Mexico City	Miguel Hidalgo	Chapter 10 (Diseases of the respiratory system)	45-54	14.9	10	0.1363
Mexico City	Miguel Hidalgo	Chapter 10 (Diseases of the respiratory system)	55-64	17.4	10	0.0658
Mexico City	Miguel Hidalgo	Chapter 10 (Diseases of the respiratory system)	65+	11.6	10	0.3093
Mexico City	Miguel Hidalgo	Chapter 10 (Diseases of the respiratory system)	15-24	12.2	8	0.1439
Mexico City	Miguel Hidalgo	Chapter 10 (Diseases of the respiratory system)	25-34	7.0	10	0.7277
Mexico City	Miguel Hidalgo	Chapter 5 (Mental and behavioural disorders)	55-64	8.2	10	0.6064

Mexico City	Miguel Hidalgo	Chapter 5 (Mental and behavioural disorders)	65+	19.1	10	**0.0388***
Mexico City	Miguel Hidalgo	Chapter 5 (Mental and behavioural disorders)	45-54	18.2	10	0.0513
Mexico City	Miguel Hidalgo	Chapter 5 (Mental and behavioural disorders)	35-44	23.0	10	**0.0107***
Mexico City	Venustiano Carranza	Chapter 9 (Diseases of the circulatory system)	15-24	23.5	10	**0.0091***
Mexico City	Venustiano Carranza	Chapter 9 (Diseases of the circulatory system)	25-34	5.7	10	0.8373
Mexico City	Venustiano Carranza	Chapter 9 (Diseases of the circulatory system)	35-44	15.7	10	0.1077
Mexico City	Venustiano Carranza	Chapter 9 (Diseases of the circulatory system)	45-54	9.3	10	0.5073
Mexico City	Venustiano Carranza	Chapter 9 (Diseases of the circulatory system)	55-64	3.3	10	0.9746
Mexico City	Venustiano Carranza	Chapter 9 (Diseases of the circulatory system)	65+	8.2	10	0.6120
Mexico City	Venustiano Carranza	Chapter 9 (Diseases of the circulatory system)	5-14	2.3	4	0.6847
Mexico City	Venustiano Carranza	Chapter 9 (Diseases of the circulatory system)	0-4	1.5	6	0.9585
Mexico City	Venustiano Carranza	Chapter 4 (Endocrine, nutritional and metabolic diseases)	0-4	5.0	8	0.7616
Mexico City	Venustiano Carranza	Chapter 4 (Endocrine, nutritional and metabolic diseases)	25-34	25.5	10	**0.0045***
Mexico City	Venustiano Carranza	Chapter 4 (Endocrine, nutritional and metabolic diseases)	35-44	12.7	10	0.2422
Mexico City	Venustiano Carranza	Chapter 4 (Endocrine, nutritional and metabolic diseases)	45-54	11.9	10	0.2940
Mexico City	Venustiano Carranza	Chapter 4 (Endocrine, nutritional and metabolic diseases)	55-64	7.9	10	0.6363
Mexico City	Venustiano Carranza	Chapter 4 (Endocrine, nutritional and metabolic diseases)	65+	13.6	10	0.1926
Mexico City	Venustiano Carranza	Chapter 4 (Endocrine, nutritional and metabolic diseases)	15-24	10.4	8	0.2408
Mexico City	Venustiano Carranza	Chapter 6 (Diseases of the nervous system)	0-4	6.9	10	0.7368
Mexico City	Venustiano Carranza	Chapter 6 (Diseases of the nervous system)	25-34	11.4	10	0.3295
Mexico City	Venustiano Carranza	Chapter 6 (Diseases of the nervous system)	35-44	16.5	10	0.0859
Mexico City	Venustiano Carranza	Chapter 6 (Diseases of the nervous system)	45-54	16.6	10	0.0847
Mexico City	Venustiano Carranza	Chapter 6 (Diseases of the nervous system)	55-64	4.4	10	0.9259
Mexico City	Venustiano Carranza	Chapter 6 (Diseases of the nervous system)	65+	16.4	10	0.0892
Mexico City	Venustiano Carranza	Chapter 6 (Diseases of the nervous system)	5-14	19.9	9	**0.0184***
Mexico City	Venustiano Carranza	Chapter 6 (Diseases of the nervous system)	15-24	8.2	10	0.6059
Mexico City	Venustiano Carranza	Chapter 10 (Diseases of the respiratory system)	0-4	9.0	10	0.5302
Mexico City	Venustiano Carranza	Chapter 10 (Diseases of the respiratory system)	5-14	5.5	7	0.5996
Mexico City	Venustiano Carranza	Chapter 10 (Diseases of the respiratory system)	15-24	13.9	10	0.1790
Mexico City	Venustiano Carranza	Chapter 10 (Diseases of the respiratory system)	25-34	9.3	10	0.4993
Mexico City	Venustiano Carranza	Chapter 10 (Diseases of the respiratory system)	35-44	15.0	10	0.1313
Mexico City	Venustiano Carranza	Chapter 10 (Diseases of the respiratory system)	45-54	6.3	10	0.7866
Mexico City	Venustiano Carranza	Chapter 10 (Diseases of the respiratory system)	55-64	8.5	10	0.5841
Mexico City	Venustiano Carranza	Chapter 10 (Diseases of the respiratory system)	65+	8.5	10	0.5788
Mexico City	Venustiano Carranza	Chapter 5 (Mental and behavioural disorders)	35-44	4.3	10	0.9324
Mexico City	Venustiano Carranza	Chapter 5 (Mental and behavioural disorders)	45-54	9.4	10	0.4973
Mexico City	Venustiano Carranza	Chapter 5 (Mental and behavioural disorders)	55-64	9.7	10	0.4633
Mexico City	Venustiano Carranza	Chapter 5 (Mental and behavioural disorders)	65+	5.8	10	0.8321
Mexico City	Venustiano Carranza	Chapter 5 (Mental and behavioural disorders)	25-34	10.4	9	0.3159
Hidalgo	Tizayuca	Chapter 9 (Diseases of the circulatory system)	35-44	6.3	10	0.7909
Hidalgo	Tizayuca	Chapter 9 (Diseases of the circulatory system)	45-54	12.7	10	0.2381
Hidalgo	Tizayuca	Chapter 9 (Diseases of the circulatory system)	55-64	13.6	10	0.1929
Hidalgo	Tizayuca	Chapter 9 (Diseases of the circulatory system)	65+	5.7	10	0.8366

Hidalgo	Tizayuca	Chapter 9 (Diseases of the circulatory system)	25-34	4.6	9	0.8639
Hidalgo	Tizayuca	Chapter 9 (Diseases of the circulatory system)	15-24	7.9	6	0.2491
Hidalgo	Tizayuca	Chapter 4 (Endocrine, nutritional and metabolic diseases)	0-4	1.7	5	0.8936
Hidalgo	Tizayuca	Chapter 4 (Endocrine, nutritional and metabolic diseases)	35-44	8.3	10	0.5973
Hidalgo	Tizayuca	Chapter 4 (Endocrine, nutritional and metabolic diseases)	55-64	10.8	10	0.3763
Hidalgo	Tizayuca	Chapter 4 (Endocrine, nutritional and metabolic diseases)	65+	12.3	10	0.2666
Hidalgo	Tizayuca	Chapter 4 (Endocrine, nutritional and metabolic diseases)	45-54	15.0	10	0.1313
Hidalgo	Tizayuca	Chapter 4 (Endocrine, nutritional and metabolic diseases)	25-34	8.7	7	0.2752
Hidalgo	Tizayuca	Chapter 6 (Diseases of the nervous system)	15-24	3.4	7	0.8496
Hidalgo	Tizayuca	Chapter 6 (Diseases of the nervous system)	45-54	2.9	5	0.7077
Hidalgo	Tizayuca	Chapter 6 (Diseases of the nervous system)	0-4	5.0	4	0.2835
Hidalgo	Tizayuca	Chapter 6 (Diseases of the nervous system)	5-14	3.7	7	0.8148
Hidalgo	Tizayuca	Chapter 6 (Diseases of the nervous system)	25-34	3.7	4	0.4497
Hidalgo	Tizayuca	Chapter 6 (Diseases of the nervous system)	65+	7.6	10	0.6685
Hidalgo	Tizayuca	Chapter 6 (Diseases of the nervous system)	55-64	6.4	5	0.2735
Hidalgo	Tizayuca	Chapter 10 (Diseases of the respiratory system)	0-4	10.8	10	0.3718
Hidalgo	Tizayuca	Chapter 10 (Diseases of the respiratory system)	65+	23.9	10	**0.0078***
Hidalgo	Tizayuca	Chapter 10 (Diseases of the respiratory system)	5-14	9.2	5	0.1016
Hidalgo	Tizayuca	Chapter 10 (Diseases of the respiratory system)	15-24	9.6	5	0.0860
Hidalgo	Tizayuca	Chapter 10 (Diseases of the respiratory system)	35-44	2.4	9	0.9837
Hidalgo	Tizayuca	Chapter 10 (Diseases of the respiratory system)	55-64	9.7	10	0.4712
Hidalgo	Tizayuca	Chapter 10 (Diseases of the respiratory system)	25-34	7.3	5	0.1977
Hidalgo	Tizayuca	Chapter 10 (Diseases of the respiratory system)	45-54	12.1	10	0.2764
Hidalgo	Tizayuca	Chapter 5 (Mental and behavioural disorders)	65+	1.6	9	0.9961
State of Mexico	Acolman	Chapter 9 (Diseases of the circulatory system)	25-34	3.6	10	0.9638
State of Mexico	Acolman	Chapter 9 (Diseases of the circulatory system)	35-44	5.0	10	0.8900
State of Mexico	Acolman	Chapter 9 (Diseases of the circulatory system)	45-54	11.7	10	0.3042
State of Mexico	Acolman	Chapter 9 (Diseases of the circulatory system)	55-64	6.8	10	0.7424
State of Mexico	Acolman	Chapter 9 (Diseases of the circulatory system)	65+	12.1	10	0.2814
State of Mexico	Acolman	Chapter 4 (Endocrine, nutritional and metabolic diseases)	0-4	1.6	7	0.9797
State of Mexico	Acolman	Chapter 4 (Endocrine, nutritional and metabolic diseases)	15-24	10.9	6	0.0903
State of Mexico	Acolman	Chapter 4 (Endocrine, nutritional and metabolic diseases)	25-34	6.5	10	0.7720
State of Mexico	Acolman	Chapter 4 (Endocrine, nutritional and metabolic diseases)	35-44	8.9	10	0.5399
State of Mexico	Acolman	Chapter 4 (Endocrine, nutritional and metabolic diseases)	45-54	13.5	10	0.1978
State of Mexico	Acolman	Chapter 4 (Endocrine, nutritional and metabolic diseases)	55-64	7.2	10	0.7106
State of Mexico	Acolman	Chapter 4 (Endocrine, nutritional and metabolic diseases)	65+	9.8	10	0.4622
State of Mexico	Acolman	Chapter 6 (Diseases of the nervous system)	25-34	6.1	8	0.6390
State of Mexico	Acolman	Chapter 6 (Diseases of the nervous system)	35-44	19.1	10	**0.0386***
State of Mexico	Acolman	Chapter 6 (Diseases of the nervous system)	45-54	5.4	10	0.8639
State of Mexico	Acolman	Chapter 6 (Diseases of the nervous system)	15-24	4.5	5	0.4810
State of Mexico	Acolman	Chapter 6 (Diseases of the nervous system)	55-64	3.2	8	0.9209
State of Mexico	Acolman	Chapter 6 (Diseases of the nervous system)	65+	6.7	10	0.7529
State of Mexico	Acolman	Chapter 6 (Diseases of the nervous system)	0-4	2.2	4	0.7072

State of Mexico	Acolman	Chapter 10 (Diseases of the respiratory system)	0-4	4.3	10	0.9307
State of Mexico	Acolman	Chapter 10 (Diseases of the respiratory system)	25-34	2.2	4	0.7014
State of Mexico	Acolman	Chapter 10 (Diseases of the respiratory system)	35-44	1.5	9	0.9970
State of Mexico	Acolman	Chapter 10 (Diseases of the respiratory system)	55-64	10.6	10	0.3888
State of Mexico	Acolman	Chapter 10 (Diseases of the respiratory system)	65+	9.4	10	0.4958
State of Mexico	Acolman	Chapter 10 (Diseases of the respiratory system)	45-54	14.4	9	0.1084
State of Mexico	Acolman	Chapter 5 (Mental and behavioural disorders)	45-54	4.6	8	0.7995
State of Mexico	Acolman	Chapter 5 (Mental and behavioural disorders)	65+	21.5	10	**0.0180***
State of Mexico	Amecameca	Chapter 9 (Diseases of the circulatory system)	15-24	2.1	8	0.9776
State of Mexico	Amecameca	Chapter 9 (Diseases of the circulatory system)	35-44	13.7	10	0.1848
State of Mexico	Amecameca	Chapter 9 (Diseases of the circulatory system)	45-54	9.3	10	0.5074
State of Mexico	Amecameca	Chapter 9 (Diseases of the circulatory system)	55-64	4.7	10	0.9117
State of Mexico	Amecameca	Chapter 9 (Diseases of the circulatory system)	65+	10.8	10	0.3707
State of Mexico	Amecameca	Chapter 9 (Diseases of the circulatory system)	25-34	2.3	5	0.8128
State of Mexico	Amecameca	Chapter 4 (Endocrine, nutritional and metabolic diseases)	35-44	8.3	10	0.6010
State of Mexico	Amecameca	Chapter 4 (Endocrine, nutritional and metabolic diseases)	45-54	9.6	10	0.4789
State of Mexico	Amecameca	Chapter 4 (Endocrine, nutritional and metabolic diseases)	65+	5.2	10	0.8795
State of Mexico	Amecameca	Chapter 4 (Endocrine, nutritional and metabolic diseases)	0-4	8.7	8	0.3700
State of Mexico	Amecameca	Chapter 4 (Endocrine, nutritional and metabolic diseases)	55-64	10.4	10	0.4090
State of Mexico	Amecameca	Chapter 4 (Endocrine, nutritional and metabolic diseases)	25-34	7.2	5	0.2039
State of Mexico	Amecameca	Chapter 6 (Diseases of the nervous system)	35-44	3.8	4	0.4404
State of Mexico	Amecameca	Chapter 6 (Diseases of the nervous system)	55-64	2.2	4	0.6914
State of Mexico	Amecameca	Chapter 6 (Diseases of the nervous system)	65+	6.9	10	0.7389
State of Mexico	Amecameca	Chapter 6 (Diseases of the nervous system)	45-54	5.1	5	0.3995
State of Mexico	Amecameca	Chapter 6 (Diseases of the nervous system)	0-4	4.2	6	0.6492
State of Mexico	Amecameca	Chapter 6 (Diseases of the nervous system)	5-14	3.8	5	0.5795
State of Mexico	Amecameca	Chapter 6 (Diseases of the nervous system)	15-24	2.9	5	0.7224
State of Mexico	Amecameca	Chapter 10 (Diseases of the respiratory system)	0-4	6.9	10	0.7378
State of Mexico	Amecameca	Chapter 10 (Diseases of the respiratory system)	25-34	3.3	4	0.5079
State of Mexico	Amecameca	Chapter 10 (Diseases of the respiratory system)	55-64	6.9	10	0.7347
State of Mexico	Amecameca	Chapter 10 (Diseases of the respiratory system)	65+	10.2	10	0.4190
State of Mexico	Amecameca	Chapter 10 (Diseases of the respiratory system)	45-54	2.3	7	0.9418
State of Mexico	Amecameca	Chapter 10 (Diseases of the respiratory system)	15-24	7.0	4	0.1364
State of Mexico	Amecameca	Chapter 5 (Mental and behavioural disorders)	55-64	4.4	4	0.3546
State of Mexico	Amecameca	Chapter 5 (Mental and behavioural disorders)	65+	9.9	10	0.4449
State of Mexico	Amecameca	Chapter 5 (Mental and behavioural disorders)	45-54	24.5	10	**0.0064***
State of Mexico	Apaxco	Chapter 9 (Diseases of the circulatory system)	65+	14.1	10	0.1693
State of Mexico	Apaxco	Chapter 9 (Diseases of the circulatory system)	45-54	5.9	7	0.5519
State of Mexico	Apaxco	Chapter 9 (Diseases of the circulatory system)	55-64	6.2	9	0.7209
State of Mexico	Apaxco	Chapter 4 (Endocrine, nutritional and metabolic diseases)	35-44	12.8	6	**0.0471***
State of Mexico	Apaxco	Chapter 4 (Endocrine, nutritional and metabolic diseases)	45-54	9.9	10	0.4498
State of Mexico	Apaxco	Chapter 4 (Endocrine, nutritional and metabolic diseases)	65+	6.9	10	0.7391
State of Mexico	Apaxco	Chapter 4 (Endocrine, nutritional and metabolic diseases)	55-64	5.8	10	0.8326

State of Mexico	Apaxco	Chapter 6 (Diseases of the nervous system)	35-44	4.4	6	0.6164
State of Mexico	Apaxco	Chapter 6 (Diseases of the nervous system)	25-34	2.1	7	0.9570
State of Mexico	Apaxco	Chapter 6 (Diseases of the nervous system)	65+	9.1	9	0.4256
State of Mexico	Apaxco	Chapter 10 (Diseases of the respiratory system)	65+	15.7	10	0.1093
State of Mexico	Apaxco	Chapter 10 (Diseases of the respiratory system)	0-4	8.6	8	0.3776
State of Mexico	Apaxco	Chapter 5 (Mental and behavioural disorders)	65+	1.7	7	0.9730
State of Mexico	Atenco	Chapter 9 (Diseases of the circulatory system)	45-54	13.2	10	0.2126
State of Mexico	Atenco	Chapter 9 (Diseases of the circulatory system)	55-64	6.8	10	0.7412
State of Mexico	Atenco	Chapter 9 (Diseases of the circulatory system)	65+	5.2	10	0.8754
State of Mexico	Atenco	Chapter 9 (Diseases of the circulatory system)	35-44	4.4	4	0.3502
State of Mexico	Atenco	Chapter 9 (Diseases of the circulatory system)	15-24	3.8	4	0.4369
State of Mexico	Atenco	Chapter 4 (Endocrine, nutritional and metabolic diseases)	45-54	6.2	10	0.7977
State of Mexico	Atenco	Chapter 4 (Endocrine, nutritional and metabolic diseases)	55-64	22.3	10	**0.0138***
State of Mexico	Atenco	Chapter 4 (Endocrine, nutritional and metabolic diseases)	65+	11.6	10	0.3147
State of Mexico	Atenco	Chapter 4 (Endocrine, nutritional and metabolic diseases)	35-44	8.6	10	0.5671
State of Mexico	Atenco	Chapter 6 (Diseases of the nervous system)	65+	12.2	10	0.2689
State of Mexico	Atenco	Chapter 10 (Diseases of the respiratory system)	0-4	5.3	10	0.8701
State of Mexico	Atenco	Chapter 10 (Diseases of the respiratory system)	55-64	2.2	8	0.9728
State of Mexico	Atenco	Chapter 10 (Diseases of the respiratory system)	65+	12.5	10	0.2511
State of Mexico	Atenco	Chapter 10 (Diseases of the respiratory system)	45-54	1.4	4	0.8364
State of Mexico	Atenco	Chapter 5 (Mental and behavioural disorders)	65+	4.1	5	0.5334
State of Mexico	Atizapán de Zaragoza	Chapter 9 (Diseases of the circulatory system)	0-4	7.4	10	0.6894
State of Mexico	Atizapán de Zaragoza	Chapter 9 (Diseases of the circulatory system)	15-24	10.6	10	0.3919
State of Mexico	Atizapán de Zaragoza	Chapter 9 (Diseases of the circulatory system)	25-34	8.7	10	0.5586
State of Mexico	Atizapán de Zaragoza	Chapter 9 (Diseases of the circulatory system)	35-44	10.6	10	0.3880
State of Mexico	Atizapán de Zaragoza	Chapter 9 (Diseases of the circulatory system)	45-54	14.4	10	0.1547
State of Mexico	Atizapán de Zaragoza	Chapter 9 (Diseases of the circulatory system)	55-64	13.1	10	0.2198
State of Mexico	Atizapán de Zaragoza	Chapter 9 (Diseases of the circulatory system)	65+	8.3	10	0.5978
State of Mexico	Atizapán de Zaragoza	Chapter 9 (Diseases of the circulatory system)	5-14	3.3	4	0.5071
State of Mexico	Atizapán de Zaragoza	Chapter 4 (Endocrine, nutritional and metabolic diseases)	0-4	10.0	10	0.4409
State of Mexico	Atizapán de Zaragoza	Chapter 4 (Endocrine, nutritional and metabolic diseases)	5-14	9.0	8	0.3436
State of Mexico	Atizapán de Zaragoza	Chapter 4 (Endocrine, nutritional and metabolic diseases)	15-24	4.8	6	0.5716
State of Mexico	Atizapán de Zaragoza	Chapter 4 (Endocrine, nutritional and metabolic diseases)	35-44	9.2	10	0.5136
State of Mexico	Atizapán de Zaragoza	Chapter 4 (Endocrine, nutritional and metabolic diseases)	45-54	11.9	10	0.2893
State of Mexico	Atizapán de Zaragoza	Chapter 4 (Endocrine, nutritional and metabolic diseases)	55-64	11.1	10	0.3498
State of Mexico	Atizapán de Zaragoza	Chapter 4 (Endocrine, nutritional and metabolic diseases)	65+	13.3	10	0.2070
State of Mexico	Atizapán de Zaragoza	Chapter 4 (Endocrine, nutritional and metabolic diseases)	25-34	12.7	10	0.2380
State of Mexico	Atizapán de Zaragoza	Chapter 6 (Diseases of the nervous system)	0-4	8.3	10	0.5993
State of Mexico	Atizapán de Zaragoza	Chapter 6 (Diseases of the nervous system)	5-14	8.9	10	0.5392
State of Mexico	Atizapán de Zaragoza	Chapter 6 (Diseases of the nervous system)	15-24	18.1	10	0.0540
State of Mexico	Atizapán de Zaragoza	Chapter 6 (Diseases of the nervous system)	25-34	6.8	10	0.7453
State of Mexico	Atizapán de Zaragoza	Chapter 6 (Diseases of the nervous system)	35-44	11.5	10	0.3207
State of Mexico	Atizapán de Zaragoza	Chapter 6 (Diseases of the nervous system)	65+	13.1	10	0.2169

State of Mexico	Atizapán de Zaragoza	Chapter 6 (Diseases of the nervous system)	45-54	7.3	10	0.6970
State of Mexico	Atizapán de Zaragoza	Chapter 6 (Diseases of the nervous system)	55-64	12.0	10	0.2863
State of Mexico	Atizapán de Zaragoza	Chapter 10 (Diseases of the respiratory system)	0-4	17.0	10	0.0737
State of Mexico	Atizapán de Zaragoza	Chapter 10 (Diseases of the respiratory system)	25-34	12.3	10	0.2668
State of Mexico	Atizapán de Zaragoza	Chapter 10 (Diseases of the respiratory system)	35-44	9.9	10	0.4484
State of Mexico	Atizapán de Zaragoza	Chapter 10 (Diseases of the respiratory system)	45-54	9.9	10	0.4532
State of Mexico	Atizapán de Zaragoza	Chapter 10 (Diseases of the respiratory system)	55-64	6.7	10	0.7513
State of Mexico	Atizapán de Zaragoza	Chapter 10 (Diseases of the respiratory system)	65+	8.4	10	0.5921
State of Mexico	Atizapán de Zaragoza	Chapter 10 (Diseases of the respiratory system)	5-14	3.4	8	0.9070
State of Mexico	Atizapán de Zaragoza	Chapter 10 (Diseases of the respiratory system)	15-24	5.9	10	0.8249
State of Mexico	Atizapán de Zaragoza	Chapter 5 (Mental and behavioural disorders)	35-44	18.1	10	0.0533
State of Mexico	Atizapán de Zaragoza	Chapter 5 (Mental and behavioural disorders)	45-54	12.5	10	0.2557
State of Mexico	Atizapán de Zaragoza	Chapter 5 (Mental and behavioural disorders)	55-64	8.1	10	0.6214
State of Mexico	Atizapán de Zaragoza	Chapter 5 (Mental and behavioural disorders)	65+	8.7	10	0.5560
State of Mexico	Atizapán de Zaragoza	Chapter 5 (Mental and behavioural disorders)	25-34	8.0	6	0.2400
State of Mexico	Atlautla	Chapter 9 (Diseases of the circulatory system)	45-54	13.0	10	0.2229
State of Mexico	Atlautla	Chapter 9 (Diseases of the circulatory system)	55-64	13.6	10	0.1911
State of Mexico	Atlautla	Chapter 9 (Diseases of the circulatory system)	65+	8.7	10	0.5611
State of Mexico	Atlautla	Chapter 9 (Diseases of the circulatory system)	35-44	2.6	7	0.9168
State of Mexico	Atlautla	Chapter 4 (Endocrine, nutritional and metabolic diseases)	25-34	20.2	6	**0.0026**
State of Mexico	Atlautla	Chapter 4 (Endocrine, nutritional and metabolic diseases)	35-44	9.9	10	0.4526
State of Mexico	Atlautla	Chapter 4 (Endocrine, nutritional and metabolic diseases)	45-54	9.9	10	0.4504
State of Mexico	Atlautla	Chapter 4 (Endocrine, nutritional and metabolic diseases)	55-64	10.5	10	0.3989
State of Mexico	Atlautla	Chapter 4 (Endocrine, nutritional and metabolic diseases)	65+	9.3	10	0.5026
State of Mexico	Atlautla	Chapter 6 (Diseases of the nervous system)	25-34	10.3	5	0.0676
State of Mexico	Atlautla	Chapter 6 (Diseases of the nervous system)	65+	5.6	6	0.4660
State of Mexico	Atlautla	Chapter 10 (Diseases of the respiratory system)	0-4	8.2	10	0.6117
State of Mexico	Atlautla	Chapter 10 (Diseases of the respiratory system)	45-54	2.0	4	0.7329
State of Mexico	Atlautla	Chapter 10 (Diseases of the respiratory system)	55-64	10.2	6	0.1175
State of Mexico	Atlautla	Chapter 10 (Diseases of the respiratory system)	65+	19.6	10	**0.0333***
State of Mexico	Atlautla	Chapter 5 (Mental and behavioural disorders)	65+	6.5	4	0.1622
State of Mexico	Axapusco	Chapter 9 (Diseases of the circulatory system)	45-54	4.5	10	0.9224
State of Mexico	Axapusco	Chapter 9 (Diseases of the circulatory system)	55-64	8.4	10	0.5869
State of Mexico	Axapusco	Chapter 9 (Diseases of the circulatory system)	65+	28.2	10	**0.0016***
State of Mexico	Axapusco	Chapter 9 (Diseases of the circulatory system)	35-44	7.8	6	0.2501
State of Mexico	Axapusco	Chapter 4 (Endocrine, nutritional and metabolic diseases)	45-54	8.3	10	0.5977
State of Mexico	Axapusco	Chapter 4 (Endocrine, nutritional and metabolic diseases)	55-64	4.3	10	0.9316
State of Mexico	Axapusco	Chapter 4 (Endocrine, nutritional and metabolic diseases)	65+	13.4	10	0.2005
State of Mexico	Axapusco	Chapter 4 (Endocrine, nutritional and metabolic diseases)	25-34	1.7	7	0.9738
State of Mexico	Axapusco	Chapter 4 (Endocrine, nutritional and metabolic diseases)	35-44	2.9	4	0.5790
State of Mexico	Axapusco	Chapter 6 (Diseases of the nervous system)	35-44	5.1	4	0.2748
State of Mexico	Axapusco	Chapter 6 (Diseases of the nervous system)	65+	15.0	7	**0.0354***
State of Mexico	Axapusco	Chapter 10 (Diseases of the respiratory system)	0-4	5.5	7	0.6030

State of Mexico	Axapusco	Chapter 10 (Diseases of the respiratory system)	65+	23.6	10	**0.0087***
State of Mexico	Axapusco	Chapter 10 (Diseases of the respiratory system)	55-64	1.8	5	0.8794
State of Mexico	Axapusco	Chapter 5 (Mental and behavioural disorders)	65+	9.4	6	0.1529
State of Mexico	Axapusco	Chapter 5 (Mental and behavioural disorders)	35-44	2.8	4	0.5951
State of Mexico	Ayapango	Chapter 9 (Diseases of the circulatory system)	65+	8.7	10	0.5579
State of Mexico	Ayapango	Chapter 9 (Diseases of the circulatory system)	55-64	7.7	5	0.1745
State of Mexico	Ayapango	Chapter 4 (Endocrine, nutritional and metabolic diseases)	65+	4.8	10	0.9039
State of Mexico	Ayapango	Chapter 4 (Endocrine, nutritional and metabolic diseases)	55-64	12.2	8	0.1414
State of Mexico	Ayapango	Chapter 6 (Diseases of the nervous system)	65+	6.5	6	0.3655
State of Mexico	Ayapango	Chapter 10 (Diseases of the respiratory system)	0-4	5.8	4	0.2118
State of Mexico	Ayapango	Chapter 10 (Diseases of the respiratory system)	65+	5.9	10	0.8229
State of Mexico	Coacalco de Berriozábal	Chapter 9 (Diseases of the circulatory system)	15-24	17.3	10	0.0682
State of Mexico	Coacalco de Berriozábal	Chapter 9 (Diseases of the circulatory system)	45-54	6.2	10	0.7967
State of Mexico	Coacalco de Berriozábal	Chapter 9 (Diseases of the circulatory system)	55-64	15.1	10	0.1282
State of Mexico	Coacalco de Berriozábal	Chapter 9 (Diseases of the circulatory system)	65+	9.1	10	0.5236
State of Mexico	Coacalco de Berriozábal	Chapter 9 (Diseases of the circulatory system)	25-34	8.1	10	0.6143
State of Mexico	Coacalco de Berriozábal	Chapter 9 (Diseases of the circulatory system)	35-44	12.3	10	0.2685
State of Mexico	Coacalco de Berriozábal	Chapter 9 (Diseases of the circulatory system)	0-4	8.5	6	0.2041
State of Mexico	Coacalco de Berriozábal	Chapter 4 (Endocrine, nutritional and metabolic diseases)	15-24	1.7	6	0.9417
State of Mexico	Coacalco de Berriozábal	Chapter 4 (Endocrine, nutritional and metabolic diseases)	25-34	5.7	10	0.8404
State of Mexico	Coacalco de Berriozábal	Chapter 4 (Endocrine, nutritional and metabolic diseases)	35-44	15.3	10	0.1206
State of Mexico	Coacalco de Berriozábal	Chapter 4 (Endocrine, nutritional and metabolic diseases)	45-54	12.0	10	0.2874
State of Mexico	Coacalco de Berriozábal	Chapter 4 (Endocrine, nutritional and metabolic diseases)	55-64	19.4	10	**0.0355***
State of Mexico	Coacalco de Berriozábal	Chapter 4 (Endocrine, nutritional and metabolic diseases)	65+	9.1	10	0.5265
State of Mexico	Coacalco de Berriozábal	Chapter 4 (Endocrine, nutritional and metabolic diseases)	0-4	12.2	8	0.1423
State of Mexico	Coacalco de Berriozábal	Chapter 6 (Diseases of the nervous system)	35-44	9.9	10	0.4514
State of Mexico	Coacalco de Berriozábal	Chapter 6 (Diseases of the nervous system)	45-54	6.5	10	0.7700
State of Mexico	Coacalco de Berriozábal	Chapter 6 (Diseases of the nervous system)	55-64	6.9	10	0.7344
State of Mexico	Coacalco de Berriozábal	Chapter 6 (Diseases of the nervous system)	65+	7.4	10	0.6892
State of Mexico	Coacalco de Berriozábal	Chapter 6 (Diseases of the nervous system)	5-14	1.4	9	0.9976
State of Mexico	Coacalco de Berriozábal	Chapter 6 (Diseases of the nervous system)	0-4	6.9	4	0.1439
State of Mexico	Coacalco de Berriozábal	Chapter 6 (Diseases of the nervous system)	15-24	13.7	10	0.1853
State of Mexico	Coacalco de Berriozábal	Chapter 6 (Diseases of the nervous system)	25-34	11.3	7	0.1270
State of Mexico	Coacalco de Berriozábal	Chapter 10 (Diseases of the respiratory system)	0-4	10.9	10	0.3644
State of Mexico	Coacalco de Berriozábal	Chapter 10 (Diseases of the respiratory system)	25-34	7.9	10	0.6400
State of Mexico	Coacalco de Berriozábal	Chapter 10 (Diseases of the respiratory system)	35-44	14.8	10	0.1403
State of Mexico	Coacalco de Berriozábal	Chapter 10 (Diseases of the respiratory system)	45-54	5.9	10	0.8275
State of Mexico	Coacalco de Berriozábal	Chapter 10 (Diseases of the respiratory system)	55-64	8.7	10	0.5610
State of Mexico	Coacalco de Berriozábal	Chapter 10 (Diseases of the respiratory system)	65+	28.4	10	**0.0015***
State of Mexico	Coacalco de Berriozábal	Chapter 10 (Diseases of the respiratory system)	5-14	7.3	5	0.1981
State of Mexico	Coacalco de Berriozábal	Chapter 10 (Diseases of the respiratory system)	15-24	4.2	6	0.6487
State of Mexico	Coacalco de Berriozábal	Chapter 5 (Mental and behavioural disorders)	45-54	3.3	10	0.9722
State of Mexico	Coacalco de Berriozábal	Chapter 5 (Mental and behavioural disorders)	65+	16.1	10	0.0967

State of Mexico	Coacalco de Berriozábal	Chapter 5 (Mental and behavioural disorders)	55-64	6.7	7	0.4603
State of Mexico	Coacalco de Berriozábal	Chapter 5 (Mental and behavioural disorders)	35-44	8.9	6	0.1786
State of Mexico	Cocotitlán	Chapter 9 (Diseases of the circulatory system)	35-44	2.7	4	0.6018
State of Mexico	Cocotitlán	Chapter 9 (Diseases of the circulatory system)	55-64	20.0	10	**0.0291***
State of Mexico	Cocotitlán	Chapter 9 (Diseases of the circulatory system)	65+	7.4	10	0.6913
State of Mexico	Cocotitlán	Chapter 9 (Diseases of the circulatory system)	45-54	4.1	5	0.5327
State of Mexico	Cocotitlán	Chapter 4 (Endocrine, nutritional and metabolic diseases)	45-54	8.0	10	0.6298
State of Mexico	Cocotitlán	Chapter 4 (Endocrine, nutritional and metabolic diseases)	55-64	12.3	10	0.2665
State of Mexico	Cocotitlán	Chapter 4 (Endocrine, nutritional and metabolic diseases)	65+	6.5	10	0.7696
State of Mexico	Cocotitlán	Chapter 4 (Endocrine, nutritional and metabolic diseases)	35-44	4.0	5	0.5474
State of Mexico	Cocotitlán	Chapter 10 (Diseases of the respiratory system)	0-4	9.6	9	0.3875
State of Mexico	Cocotitlán	Chapter 10 (Diseases of the respiratory system)	65+	7.1	10	0.7129
State of Mexico	Cocotitlán	Chapter 10 (Diseases of the respiratory system)	55-64	1.9	4	0.7596
State of Mexico	Coyotepec	Chapter 9 (Diseases of the circulatory system)	45-54	10.3	10	0.4147
State of Mexico	Coyotepec	Chapter 9 (Diseases of the circulatory system)	55-64	21.0	10	**0.0213***
State of Mexico	Coyotepec	Chapter 9 (Diseases of the circulatory system)	65+	7.2	10	0.7034
State of Mexico	Coyotepec	Chapter 9 (Diseases of the circulatory system)	35-44	2.3	6	0.8862
State of Mexico	Coyotepec	Chapter 9 (Diseases of the circulatory system)	25-34	14.5	10	0.1504
State of Mexico	Coyotepec	Chapter 4 (Endocrine, nutritional and metabolic diseases)	55-64	8.3	10	0.5987
State of Mexico	Coyotepec	Chapter 4 (Endocrine, nutritional and metabolic diseases)	65+	5.2	10	0.8801
State of Mexico	Coyotepec	Chapter 4 (Endocrine, nutritional and metabolic diseases)	35-44	2.4	8	0.9678
State of Mexico	Coyotepec	Chapter 4 (Endocrine, nutritional and metabolic diseases)	45-54	5.7	10	0.8409
State of Mexico	Coyotepec	Chapter 4 (Endocrine, nutritional and metabolic diseases)	0-4	6.3	5	0.2818
State of Mexico	Coyotepec	Chapter 4 (Endocrine, nutritional and metabolic diseases)	25-34	2.9	6	0.8265
State of Mexico	Coyotepec	Chapter 6 (Diseases of the nervous system)	65+	8.0	7	0.3301
State of Mexico	Coyotepec	Chapter 10 (Diseases of the respiratory system)	0-4	17.2	10	0.0693
State of Mexico	Coyotepec	Chapter 10 (Diseases of the respiratory system)	45-54	8.3	5	0.1426
State of Mexico	Coyotepec	Chapter 10 (Diseases of the respiratory system)	55-64	16.9	9	0.0509
State of Mexico	Coyotepec	Chapter 10 (Diseases of the respiratory system)	65+	12.4	10	0.2606
State of Mexico	Cuautitlán	Chapter 9 (Diseases of the circulatory system)	35-44	9.1	10	0.5242
State of Mexico	Cuautitlán	Chapter 9 (Diseases of the circulatory system)	45-54	6.4	10	0.7799
State of Mexico	Cuautitlán	Chapter 9 (Diseases of the circulatory system)	55-64	5.2	10	0.8797
State of Mexico	Cuautitlán	Chapter 9 (Diseases of the circulatory system)	65+	11.8	10	0.2999
State of Mexico	Cuautitlán	Chapter 9 (Diseases of the circulatory system)	25-34	25.8	10	**0.0040***
State of Mexico	Cuautitlán	Chapter 9 (Diseases of the circulatory system)	0-4	4.2	5	0.5161
State of Mexico	Cuautitlán	Chapter 9 (Diseases of the circulatory system)	15-24	11.8	7	0.1084
State of Mexico	Cuautitlán	Chapter 4 (Endocrine, nutritional and metabolic diseases)	0-4	11.1	10	0.3477
State of Mexico	Cuautitlán	Chapter 4 (Endocrine, nutritional and metabolic diseases)	25-34	18.3	10	0.0507
State of Mexico	Cuautitlán	Chapter 4 (Endocrine, nutritional and metabolic diseases)	35-44	6.6	10	0.7606
State of Mexico	Cuautitlán	Chapter 4 (Endocrine, nutritional and metabolic diseases)	45-54	7.9	10	0.6415
State of Mexico	Cuautitlán	Chapter 4 (Endocrine, nutritional and metabolic diseases)	55-64	8.8	10	0.5481
State of Mexico	Cuautitlán	Chapter 4 (Endocrine, nutritional and metabolic diseases)	65+	6.7	10	0.7531
State of Mexico	Cuautitlán	Chapter 6 (Diseases of the nervous system)	5-14	2.2	9	0.9880

State of Mexico	Cuautitlán	Chapter 6 (Diseases of the nervous system)	65+	7.9	10	0.6432
State of Mexico	Cuautitlán	Chapter 6 (Diseases of the nervous system)	0-4	3.1	6	0.8004
State of Mexico	Cuautitlán	Chapter 6 (Diseases of the nervous system)	25-34	3.0	4	0.5615
State of Mexico	Cuautitlán	Chapter 6 (Diseases of the nervous system)	55-64	2.2	6	0.9010
State of Mexico	Cuautitlán	Chapter 10 (Diseases of the respiratory system)	0-4	6.0	10	0.8192
State of Mexico	Cuautitlán	Chapter 10 (Diseases of the respiratory system)	45-54	8.0	10	0.6270
State of Mexico	Cuautitlán	Chapter 10 (Diseases of the respiratory system)	55-64	4.0	10	0.9470
State of Mexico	Cuautitlán	Chapter 10 (Diseases of the respiratory system)	65+	20.6	10	**0.0240***
State of Mexico	Cuautitlán	Chapter 10 (Diseases of the respiratory system)	35-44	6.0	7	0.5415
State of Mexico	Cuautitlán	Chapter 10 (Diseases of the respiratory system)	25-34	6.6	8	0.5790
State of Mexico	Cuautitlán	Chapter 5 (Mental and behavioural disorders)	35-44	3.1	4	0.5336
State of Mexico	Cuautitlán	Chapter 5 (Mental and behavioural disorders)	65+	2.1	8	0.9783
State of Mexico	Chalco	Chapter 9 (Diseases of the circulatory system)	15-24	5.2	10	0.8797
State of Mexico	Chalco	Chapter 9 (Diseases of the circulatory system)	35-44	16.1	10	0.0968
State of Mexico	Chalco	Chapter 9 (Diseases of the circulatory system)	45-54	6.3	10	0.7920
State of Mexico	Chalco	Chapter 9 (Diseases of the circulatory system)	55-64	9.7	10	0.4640
State of Mexico	Chalco	Chapter 9 (Diseases of the circulatory system)	65+	13.8	10	0.1844
State of Mexico	Chalco	Chapter 9 (Diseases of the circulatory system)	25-34	17.3	10	0.0688
State of Mexico	Chalco	Chapter 9 (Diseases of the circulatory system)	0-4	8.8	8	0.3572
State of Mexico	Chalco	Chapter 4 (Endocrine, nutritional and metabolic diseases)	0-4	15.5	10	0.1139
State of Mexico	Chalco	Chapter 4 (Endocrine, nutritional and metabolic diseases)	15-24	15.4	10	0.1192
State of Mexico	Chalco	Chapter 4 (Endocrine, nutritional and metabolic diseases)	25-34	40.0	10	**0.0000***
State of Mexico	Chalco	Chapter 4 (Endocrine, nutritional and metabolic diseases)	35-44	9.4	10	0.4962
State of Mexico	Chalco	Chapter 4 (Endocrine, nutritional and metabolic diseases)	45-54	12.9	10	0.2269
State of Mexico	Chalco	Chapter 4 (Endocrine, nutritional and metabolic diseases)	55-64	12.9	10	0.2297
State of Mexico	Chalco	Chapter 4 (Endocrine, nutritional and metabolic diseases)	65+	18.7	10	**0.0442***
State of Mexico	Chalco	Chapter 4 (Endocrine, nutritional and metabolic diseases)	5-14	2.4	4	0.6698
State of Mexico	Chalco	Chapter 6 (Diseases of the nervous system)	0-4	10.2	10	0.4217
State of Mexico	Chalco	Chapter 6 (Diseases of the nervous system)	5-14	8.0	10	0.6315
State of Mexico	Chalco	Chapter 6 (Diseases of the nervous system)	15-24	9.6	10	0.4735
State of Mexico	Chalco	Chapter 6 (Diseases of the nervous system)	35-44	9.6	10	0.4728
State of Mexico	Chalco	Chapter 6 (Diseases of the nervous system)	45-54	9.7	10	0.4666
State of Mexico	Chalco	Chapter 6 (Diseases of the nervous system)	55-64	9.0	10	0.5318
State of Mexico	Chalco	Chapter 6 (Diseases of the nervous system)	25-34	4.8	10	0.9015
State of Mexico	Chalco	Chapter 6 (Diseases of the nervous system)	65+	7.6	10	0.6638
State of Mexico	Chalco	Chapter 10 (Diseases of the respiratory system)	0-4	24.8	10	**0.0056***
State of Mexico	Chalco	Chapter 10 (Diseases of the respiratory system)	15-24	20.9	10	**0.0220***
State of Mexico	Chalco	Chapter 10 (Diseases of the respiratory system)	25-34	6.6	10	0.7604
State of Mexico	Chalco	Chapter 10 (Diseases of the respiratory system)	45-54	9.7	10	0.4686
State of Mexico	Chalco	Chapter 10 (Diseases of the respiratory system)	55-64	4.7	10	0.9111
State of Mexico	Chalco	Chapter 10 (Diseases of the respiratory system)	65+	12.9	10	0.2269
State of Mexico	Chalco	Chapter 10 (Diseases of the respiratory system)	35-44	7.0	10	0.7280
State of Mexico	Chalco	Chapter 10 (Diseases of the respiratory system)	5-14	3.0	5	0.7066

State of Mexico	Chalco	Chapter 5 (Mental and behavioural disorders)	25-34	4.6	8	0.8023
State of Mexico	Chalco	Chapter 5 (Mental and behavioural disorders)	45-54	15.5	10	0.1136
State of Mexico	Chalco	Chapter 5 (Mental and behavioural disorders)	65+	13.4	10	0.2018
State of Mexico	Chalco	Chapter 5 (Mental and behavioural disorders)	35-44	10.9	10	0.3670
State of Mexico	Chalco	Chapter 5 (Mental and behavioural disorders)	55-64	15.4	10	0.1182
State of Mexico	Chalco	Chapter 5 (Mental and behavioural disorders)	15-24	6.1	4	0.1916
State of Mexico	Chiautla	Chapter 9 (Diseases of the circulatory system)	15-24	4.9	4	0.3015
State of Mexico	Chiautla	Chapter 9 (Diseases of the circulatory system)	25-34	4.1	5	0.5365
State of Mexico	Chiautla	Chapter 9 (Diseases of the circulatory system)	35-44	2.1	4	0.7112
State of Mexico	Chiautla	Chapter 9 (Diseases of the circulatory system)	65+	7.9	10	0.6420
State of Mexico	Chiautla	Chapter 9 (Diseases of the circulatory system)	45-54	10.1	10	0.4316
State of Mexico	Chiautla	Chapter 9 (Diseases of the circulatory system)	55-64	5.5	10	0.8563
State of Mexico	Chiautla	Chapter 4 (Endocrine, nutritional and metabolic diseases)	45-54	11.9	10	0.2926
State of Mexico	Chiautla	Chapter 4 (Endocrine, nutritional and metabolic diseases)	55-64	13.1	10	0.2173
State of Mexico	Chiautla	Chapter 4 (Endocrine, nutritional and metabolic diseases)	65+	9.1	10	0.5181
State of Mexico	Chiautla	Chapter 4 (Endocrine, nutritional and metabolic diseases)	35-44	5.4	9	0.7959
State of Mexico	Chiautla	Chapter 6 (Diseases of the nervous system)	65+	2.9	6	0.8218
State of Mexico	Chiautla	Chapter 10 (Diseases of the respiratory system)	0-4	4.7	9	0.8631
State of Mexico	Chiautla	Chapter 10 (Diseases of the respiratory system)	65+	11.3	10	0.3330
State of Mexico	Chiautla	Chapter 10 (Diseases of the respiratory system)	55-64	2.4	6	0.8842
State of Mexico	Chiautla	Chapter 10 (Diseases of the respiratory system)	45-54	6.9	5	0.2296
State of Mexico	Chiautla	Chapter 5 (Mental and behavioural disorders)	65+	2.5	7	0.9249
State of Mexico	Chicoloapan	Chapter 9 (Diseases of the circulatory system)	45-54	10.4	10	0.4080
State of Mexico	Chicoloapan	Chapter 9 (Diseases of the circulatory system)	55-64	5.7	10	0.8430
State of Mexico	Chicoloapan	Chapter 9 (Diseases of the circulatory system)	65+	7.6	10	0.6668
State of Mexico	Chicoloapan	Chapter 9 (Diseases of the circulatory system)	35-44	15.2	10	0.1257
State of Mexico	Chicoloapan	Chapter 9 (Diseases of the circulatory system)	25-34	7.4	10	0.6861
State of Mexico	Chicoloapan	Chapter 9 (Diseases of the circulatory system)	15-24	4.8	8	0.7791
State of Mexico	Chicoloapan	Chapter 4 (Endocrine, nutritional and metabolic diseases)	25-34	15.3	10	0.1206
State of Mexico	Chicoloapan	Chapter 4 (Endocrine, nutritional and metabolic diseases)	45-54	7.5	10	0.6754
State of Mexico	Chicoloapan	Chapter 4 (Endocrine, nutritional and metabolic diseases)	55-64	12.6	10	0.2476
State of Mexico	Chicoloapan	Chapter 4 (Endocrine, nutritional and metabolic diseases)	65+	14.5	10	0.1501
State of Mexico	Chicoloapan	Chapter 4 (Endocrine, nutritional and metabolic diseases)	0-4	7.2	9	0.6201
State of Mexico	Chicoloapan	Chapter 4 (Endocrine, nutritional and metabolic diseases)	35-44	23.2	10	**0.0100***
State of Mexico	Chicoloapan	Chapter 6 (Diseases of the nervous system)	15-24	20.9	10	**0.0216***
State of Mexico	Chicoloapan	Chapter 6 (Diseases of the nervous system)	0-4	3.4	5	0.6311
State of Mexico	Chicoloapan	Chapter 6 (Diseases of the nervous system)	25-34	14.9	7	**0.0369***
State of Mexico	Chicoloapan	Chapter 6 (Diseases of the nervous system)	45-54	3.4	7	0.8505
State of Mexico	Chicoloapan	Chapter 6 (Diseases of the nervous system)	35-44	8.0	6	0.2359
State of Mexico	Chicoloapan	Chapter 6 (Diseases of the nervous system)	65+	15.5	10	0.1137
State of Mexico	Chicoloapan	Chapter 6 (Diseases of the nervous system)	5-14	3.3	4	0.5129
State of Mexico	Chicoloapan	Chapter 10 (Diseases of the respiratory system)	0-4	5.0	10	0.8943
State of Mexico	Chicoloapan	Chapter 10 (Diseases of the respiratory system)	15-24	3.1	6	0.7940

State of Mexico	Chicoloapan	Chapter 10 (Diseases of the respiratory system)	45-54	18.5	10	**0.0470***
State of Mexico	Chicoloapan	Chapter 10 (Diseases of the respiratory system)	65+	11.6	10	0.3123
State of Mexico	Chicoloapan	Chapter 10 (Diseases of the respiratory system)	55-64	6.3	10	0.7854
State of Mexico	Chicoloapan	Chapter 10 (Diseases of the respiratory system)	35-44	8.0	7	0.3348
State of Mexico	Chicoloapan	Chapter 10 (Diseases of the respiratory system)	25-34	8.3	6	0.2200
State of Mexico	Chicoloapan	Chapter 5 (Mental and behavioural disorders)	35-44	20.6	9	**0.0146***
State of Mexico	Chicoloapan	Chapter 5 (Mental and behavioural disorders)	65+	6.2	9	0.7211
State of Mexico	Chicoloapan	Chapter 5 (Mental and behavioural disorders)	45-54	6.4	6	0.3808
State of Mexico	Chicoloapan	Chapter 5 (Mental and behavioural disorders)	55-64	14.6	8	0.0684
State of Mexico	Chiconcuac	Chapter 9 (Diseases of the circulatory system)	25-34	2.2	8	0.9751
State of Mexico	Chiconcuac	Chapter 9 (Diseases of the circulatory system)	55-64	10.8	10	0.3716
State of Mexico	Chiconcuac	Chapter 9 (Diseases of the circulatory system)	65+	7.8	10	0.6501
State of Mexico	Chiconcuac	Chapter 9 (Diseases of the circulatory system)	35-44	8.9	5	0.1112
State of Mexico	Chiconcuac	Chapter 9 (Diseases of the circulatory system)	45-54	5.5	9	0.7863
State of Mexico	Chiconcuac	Chapter 4 (Endocrine, nutritional and metabolic diseases)	45-54	6.0	10	0.8182
State of Mexico	Chiconcuac	Chapter 4 (Endocrine, nutritional and metabolic diseases)	55-64	7.4	10	0.6912
State of Mexico	Chiconcuac	Chapter 4 (Endocrine, nutritional and metabolic diseases)	65+	18.4	10	0.0488
State of Mexico	Chiconcuac	Chapter 4 (Endocrine, nutritional and metabolic diseases)	35-44	10.8	10	0.3737
State of Mexico	Chiconcuac	Chapter 6 (Diseases of the nervous system)	65+	5.4	5	0.3675
State of Mexico	Chiconcuac	Chapter 10 (Diseases of the respiratory system)	0-4	16.6	10	0.0843
State of Mexico	Chiconcuac	Chapter 10 (Diseases of the respiratory system)	65+	13.1	10	0.2179
State of Mexico	Chiconcuac	Chapter 10 (Diseases of the respiratory system)	55-64	4.1	5	0.5395
State of Mexico	Chiconcuac	Chapter 10 (Diseases of the respiratory system)	45-54	7.4	4	0.1166
State of Mexico	Chimalhuacán	Chapter 9 (Diseases of the circulatory system)	5-14	1.7	7	0.9743
State of Mexico	Chimalhuacán	Chapter 9 (Diseases of the circulatory system)	25-34	9.7	10	0.4657
State of Mexico	Chimalhuacán	Chapter 9 (Diseases of the circulatory system)	35-44	29.8	10	**0.0009***
State of Mexico	Chimalhuacán	Chapter 9 (Diseases of the circulatory system)	45-54	9.0	10	0.5307
State of Mexico	Chimalhuacán	Chapter 9 (Diseases of the circulatory system)	55-64	6.5	10	0.7756
State of Mexico	Chimalhuacán	Chapter 9 (Diseases of the circulatory system)	65+	10.8	10	0.3733
State of Mexico	Chimalhuacán	Chapter 9 (Diseases of the circulatory system)	15-24	11.1	10	0.3463
State of Mexico	Chimalhuacán	Chapter 9 (Diseases of the circulatory system)	0-4	7.2	10	0.7042
State of Mexico	Chimalhuacán	Chapter 4 (Endocrine, nutritional and metabolic diseases)	0-4	4.7	10	0.9101
State of Mexico	Chimalhuacán	Chapter 4 (Endocrine, nutritional and metabolic diseases)	5-14	20.4	10	**0.0261***
State of Mexico	Chimalhuacán	Chapter 4 (Endocrine, nutritional and metabolic diseases)	25-34	5.3	10	0.8689
State of Mexico	Chimalhuacán	Chapter 4 (Endocrine, nutritional and metabolic diseases)	35-44	10.8	10	0.3742
State of Mexico	Chimalhuacán	Chapter 4 (Endocrine, nutritional and metabolic diseases)	45-54	7.5	10	0.6817
State of Mexico	Chimalhuacán	Chapter 4 (Endocrine, nutritional and metabolic diseases)	55-64	6.5	10	0.7704
State of Mexico	Chimalhuacán	Chapter 4 (Endocrine, nutritional and metabolic diseases)	65+	10.3	10	0.4186
State of Mexico	Chimalhuacán	Chapter 4 (Endocrine, nutritional and metabolic diseases)	15-24	19.2	10	**0.0373***
State of Mexico	Chimalhuacán	Chapter 6 (Diseases of the nervous system)	0-4	13.9	10	0.1760
State of Mexico	Chimalhuacán	Chapter 6 (Diseases of the nervous system)	15-24	11.3	10	0.3350
State of Mexico	Chimalhuacán	Chapter 6 (Diseases of the nervous system)	25-34	13.0	10	0.2252
State of Mexico	Chimalhuacán	Chapter 6 (Diseases of the nervous system)	45-54	9.4	10	0.4969

State of Mexico	Chimalhuacán	Chapter 6 (Diseases of the nervous system)	65+	24.0	10	**0.0076***
State of Mexico	Chimalhuacán	Chapter 6 (Diseases of the nervous system)	5-14	8.7	10	0.5641
State of Mexico	Chimalhuacán	Chapter 6 (Diseases of the nervous system)	35-44	6.6	10	0.7625
State of Mexico	Chimalhuacán	Chapter 6 (Diseases of the nervous system)	55-64	9.0	10	0.5301
State of Mexico	Chimalhuacán	Chapter 10 (Diseases of the respiratory system)	0-4	7.1	10	0.7146
State of Mexico	Chimalhuacán	Chapter 10 (Diseases of the respiratory system)	5-14	8.4	9	0.4903
State of Mexico	Chimalhuacán	Chapter 10 (Diseases of the respiratory system)	15-24	13.3	10	0.2096
State of Mexico	Chimalhuacán	Chapter 10 (Diseases of the respiratory system)	25-34	11.7	10	0.3091
State of Mexico	Chimalhuacán	Chapter 10 (Diseases of the respiratory system)	35-44	12.9	10	0.2318
State of Mexico	Chimalhuacán	Chapter 10 (Diseases of the respiratory system)	45-54	2.7	10	0.9877
State of Mexico	Chimalhuacán	Chapter 10 (Diseases of the respiratory system)	55-64	2.7	10	0.9869
State of Mexico	Chimalhuacán	Chapter 10 (Diseases of the respiratory system)	65+	13.6	10	0.1939
State of Mexico	Chimalhuacán	Chapter 5 (Mental and behavioural disorders)	25-34	3.1	10	0.9796
State of Mexico	Chimalhuacán	Chapter 5 (Mental and behavioural disorders)	35-44	6.3	10	0.7853
State of Mexico	Chimalhuacán	Chapter 5 (Mental and behavioural disorders)	45-54	13.2	10	0.2111
State of Mexico	Chimalhuacán	Chapter 5 (Mental and behavioural disorders)	65+	5.0	10	0.8880
State of Mexico	Chimalhuacán	Chapter 5 (Mental and behavioural disorders)	15-24	9.7	10	0.4657
State of Mexico	Chimalhuacán	Chapter 5 (Mental and behavioural disorders)	55-64	7.4	10	0.6888
State of Mexico	Ecatepec de Morelos	Chapter 9 (Diseases of the circulatory system)	0-4	18.1	10	0.0526
State of Mexico	Ecatepec de Morelos	Chapter 9 (Diseases of the circulatory system)	15-24	9.9	10	0.4452
State of Mexico	Ecatepec de Morelos	Chapter 9 (Diseases of the circulatory system)	25-34	24.6	10	**0.0062***
State of Mexico	Ecatepec de Morelos	Chapter 9 (Diseases of the circulatory system)	35-44	3.5	10	0.9678
State of Mexico	Ecatepec de Morelos	Chapter 9 (Diseases of the circulatory system)	45-54	9.0	10	0.5291
State of Mexico	Ecatepec de Morelos	Chapter 9 (Diseases of the circulatory system)	55-64	38.0	10	**0.0000***
State of Mexico	Ecatepec de Morelos	Chapter 9 (Diseases of the circulatory system)	65+	21.0	10	**0.0214***
State of Mexico	Ecatepec de Morelos	Chapter 9 (Diseases of the circulatory system)	5-14	10.2	8	0.2503
State of Mexico	Ecatepec de Morelos	Chapter 4 (Endocrine, nutritional and metabolic diseases)	0-4	11.3	10	0.3344
State of Mexico	Ecatepec de Morelos	Chapter 4 (Endocrine, nutritional and metabolic diseases)	5-14	7.6	10	0.6630
State of Mexico	Ecatepec de Morelos	Chapter 4 (Endocrine, nutritional and metabolic diseases)	15-24	11.3	10	0.3383
State of Mexico	Ecatepec de Morelos	Chapter 4 (Endocrine, nutritional and metabolic diseases)	25-34	17.9	10	0.0561
State of Mexico	Ecatepec de Morelos	Chapter 4 (Endocrine, nutritional and metabolic diseases)	35-44	8.5	10	0.5756
State of Mexico	Ecatepec de Morelos	Chapter 4 (Endocrine, nutritional and metabolic diseases)	45-54	15.2	10	0.1248
State of Mexico	Ecatepec de Morelos	Chapter 4 (Endocrine, nutritional and metabolic diseases)	55-64	12.4	10	0.2582
State of Mexico	Ecatepec de Morelos	Chapter 4 (Endocrine, nutritional and metabolic diseases)	65+	15.2	10	0.1256
State of Mexico	Ecatepec de Morelos	Chapter 6 (Diseases of the nervous system)	0-4	14.4	10	0.1573
State of Mexico	Ecatepec de Morelos	Chapter 6 (Diseases of the nervous system)	5-14	18.7	10	**0.0439***
State of Mexico	Ecatepec de Morelos	Chapter 6 (Diseases of the nervous system)	15-24	10.5	10	0.4000
State of Mexico	Ecatepec de Morelos	Chapter 6 (Diseases of the nervous system)	25-34	5.0	10	0.8943
State of Mexico	Ecatepec de Morelos	Chapter 6 (Diseases of the nervous system)	35-44	8.7	10	0.5608
State of Mexico	Ecatepec de Morelos	Chapter 6 (Diseases of the nervous system)	45-54	13.0	10	0.2243
State of Mexico	Ecatepec de Morelos	Chapter 6 (Diseases of the nervous system)	55-64	7.2	10	0.7110
State of Mexico	Ecatepec de Morelos	Chapter 6 (Diseases of the nervous system)	65+	4.9	10	0.8984
State of Mexico	Ecatepec de Morelos	Chapter 10 (Diseases of the respiratory system)	0-4	15.1	10	0.1280

State of Mexico	Ecatepec de Morelos	Chapter 10 (Diseases of the respiratory system)	15-24	7.0	10	0.7250
State of Mexico	Ecatepec de Morelos	Chapter 10 (Diseases of the respiratory system)	25-34	11.3	10	0.3312
State of Mexico	Ecatepec de Morelos	Chapter 10 (Diseases of the respiratory system)	35-44	9.0	10	0.5326
State of Mexico	Ecatepec de Morelos	Chapter 10 (Diseases of the respiratory system)	45-54	19.3	10	**0.0364***
State of Mexico	Ecatepec de Morelos	Chapter 10 (Diseases of the respiratory system)	55-64	4.9	10	0.8953
State of Mexico	Ecatepec de Morelos	Chapter 10 (Diseases of the respiratory system)	65+	9.6	10	0.4728
State of Mexico	Ecatepec de Morelos	Chapter 10 (Diseases of the respiratory system)	5-14	18.9	10	**0.0420***
State of Mexico	Ecatepec de Morelos	Chapter 5 (Mental and behavioural disorders)	15-24	5.2	9	0.8148
State of Mexico	Ecatepec de Morelos	Chapter 5 (Mental and behavioural disorders)	25-34	12.9	10	0.2313
State of Mexico	Ecatepec de Morelos	Chapter 5 (Mental and behavioural disorders)	35-44	8.4	10	0.5927
State of Mexico	Ecatepec de Morelos	Chapter 5 (Mental and behavioural disorders)	45-54	10.3	10	0.4159
State of Mexico	Ecatepec de Morelos	Chapter 5 (Mental and behavioural disorders)	55-64	8.1	10	0.6224
State of Mexico	Ecatepec de Morelos	Chapter 5 (Mental and behavioural disorders)	65+	9.2	10	0.5132
State of Mexico	Ecatepec de Morelos	Chapter 5 (Mental and behavioural disorders)	5-14	7.8	4	0.1002
State of Mexico	Ecatzingo	Chapter 9 (Diseases of the circulatory system)	45-54	6.5	7	0.4880
State of Mexico	Ecatzingo	Chapter 9 (Diseases of the circulatory system)	65+	5.0	10	0.8927
State of Mexico	Ecatzingo	Chapter 9 (Diseases of the circulatory system)	55-64	5.3	6	0.5110
State of Mexico	Ecatzingo	Chapter 4 (Endocrine, nutritional and metabolic diseases)	0-4	2.9	4	0.5784
State of Mexico	Ecatzingo	Chapter 4 (Endocrine, nutritional and metabolic diseases)	55-64	7.4	8	0.4950
State of Mexico	Ecatzingo	Chapter 4 (Endocrine, nutritional and metabolic diseases)	65+	14.0	10	0.1724
State of Mexico	Ecatzingo	Chapter 4 (Endocrine, nutritional and metabolic diseases)	45-54	2.8	5	0.7309
State of Mexico	Ecatzingo	Chapter 10 (Diseases of the respiratory system)	0-4	9.5	7	0.2212
State of Mexico	Ecatzingo	Chapter 10 (Diseases of the respiratory system)	65+	6.8	10	0.7421
State of Mexico	Huehuetoca	Chapter 9 (Diseases of the circulatory system)	35-44	8.0	10	0.6248
State of Mexico	Huehuetoca	Chapter 9 (Diseases of the circulatory system)	45-54	4.7	10	0.9074
State of Mexico	Huehuetoca	Chapter 9 (Diseases of the circulatory system)	55-64	17.1	10	0.0721
State of Mexico	Huehuetoca	Chapter 9 (Diseases of the circulatory system)	65+	7.7	10	0.6541
State of Mexico	Huehuetoca	Chapter 9 (Diseases of the circulatory system)	15-24	12.5	5	**0.0288***
State of Mexico	Huehuetoca	Chapter 9 (Diseases of the circulatory system)	25-34	14.0	9	0.1212
State of Mexico	Huehuetoca	Chapter 4 (Endocrine, nutritional and metabolic diseases)	0-4	7.6	9	0.5789
State of Mexico	Huehuetoca	Chapter 4 (Endocrine, nutritional and metabolic diseases)	45-54	9.5	10	0.4849
State of Mexico	Huehuetoca	Chapter 4 (Endocrine, nutritional and metabolic diseases)	55-64	12.9	10	0.2268
State of Mexico	Huehuetoca	Chapter 4 (Endocrine, nutritional and metabolic diseases)	65+	12.8	10	0.2363
State of Mexico	Huehuetoca	Chapter 4 (Endocrine, nutritional and metabolic diseases)	25-34	5.7	6	0.4565
State of Mexico	Huehuetoca	Chapter 4 (Endocrine, nutritional and metabolic diseases)	35-44	7.6	10	0.6700
State of Mexico	Huehuetoca	Chapter 6 (Diseases of the nervous system)	35-44	5.2	4	0.2644
State of Mexico	Huehuetoca	Chapter 6 (Diseases of the nervous system)	45-54	3.3	5	0.6488
State of Mexico	Huehuetoca	Chapter 6 (Diseases of the nervous system)	15-24	8.2	7	0.3131
State of Mexico	Huehuetoca	Chapter 6 (Diseases of the nervous system)	65+	8.2	6	0.2247
State of Mexico	Huehuetoca	Chapter 6 (Diseases of the nervous system)	5-14	4.0	5	0.5438
State of Mexico	Huehuetoca	Chapter 10 (Diseases of the respiratory system)	0-4	15.0	10	0.1310
State of Mexico	Huehuetoca	Chapter 10 (Diseases of the respiratory system)	65+	17.4	10	0.0657
State of Mexico	Huehuetoca	Chapter 10 (Diseases of the respiratory system)	55-64	14.2	8	0.0768

State of Mexico	Huehuetoca	Chapter 10 (Diseases of the respiratory system)	45-54	6.5	5	0.2576
State of Mexico	Huehuetoca	Chapter 5 (Mental and behavioural disorders)	65+	2.3	4	0.6766
State of Mexico	Huehuetoca	Chapter 5 (Mental and behavioural disorders)	55-64	2.2	4	0.7038
State of Mexico	Hueypoxtla	Chapter 9 (Diseases of the circulatory system)	45-54	15.7	10	0.1078
State of Mexico	Hueypoxtla	Chapter 9 (Diseases of the circulatory system)	55-64	10.2	10	0.4215
State of Mexico	Hueypoxtla	Chapter 9 (Diseases of the circulatory system)	65+	6.3	10	0.7886
State of Mexico	Hueypoxtla	Chapter 9 (Diseases of the circulatory system)	35-44	4.6	8	0.7989
State of Mexico	Hueypoxtla	Chapter 4 (Endocrine, nutritional and metabolic diseases)	0-4	9.9	8	0.2727
State of Mexico	Hueypoxtla	Chapter 4 (Endocrine, nutritional and metabolic diseases)	35-44	4.7	9	0.8588
State of Mexico	Hueypoxtla	Chapter 4 (Endocrine, nutritional and metabolic diseases)	45-54	12.6	10	0.2464
State of Mexico	Hueypoxtla	Chapter 4 (Endocrine, nutritional and metabolic diseases)	55-64	6.1	10	0.8031
State of Mexico	Hueypoxtla	Chapter 4 (Endocrine, nutritional and metabolic diseases)	65+	11.1	10	0.3491
State of Mexico	Hueypoxtla	Chapter 6 (Diseases of the nervous system)	55-64	3.5	4	0.4779
State of Mexico	Hueypoxtla	Chapter 6 (Diseases of the nervous system)	65+	7.4	8	0.4971
State of Mexico	Hueypoxtla	Chapter 10 (Diseases of the respiratory system)	0-4	8.2	10	0.6073
State of Mexico	Hueypoxtla	Chapter 10 (Diseases of the respiratory system)	65+	12.3	10	0.2657
State of Mexico	Hueypoxtla	Chapter 10 (Diseases of the respiratory system)	25-34	6.4	5	0.2706
State of Mexico	Hueypoxtla	Chapter 10 (Diseases of the respiratory system)	45-54	4.6	5	0.4696
State of Mexico	Hueypoxtla	Chapter 10 (Diseases of the respiratory system)	55-64	3.1	9	0.9598
State of Mexico	Hueypoxtla	Chapter 5 (Mental and behavioural disorders)	65+	5.7	7	0.5760
State of Mexico	Huixquilucan	Chapter 9 (Diseases of the circulatory system)	0-4	7.4	8	0.4892
State of Mexico	Huixquilucan	Chapter 9 (Diseases of the circulatory system)	15-24	7.8	10	0.6463
State of Mexico	Huixquilucan	Chapter 9 (Diseases of the circulatory system)	35-44	12.7	10	0.2380
State of Mexico	Huixquilucan	Chapter 9 (Diseases of the circulatory system)	45-54	15.3	10	0.1228
State of Mexico	Huixquilucan	Chapter 9 (Diseases of the circulatory system)	55-64	8.2	10	0.6138
State of Mexico	Huixquilucan	Chapter 9 (Diseases of the circulatory system)	65+	19.5	10	**0.0338***
State of Mexico	Huixquilucan	Chapter 9 (Diseases of the circulatory system)	25-34	11.1	10	0.3464
State of Mexico	Huixquilucan	Chapter 4 (Endocrine, nutritional and metabolic diseases)	25-34	9.1	10	0.5187
State of Mexico	Huixquilucan	Chapter 4 (Endocrine, nutritional and metabolic diseases)	35-44	6.5	10	0.7705
State of Mexico	Huixquilucan	Chapter 4 (Endocrine, nutritional and metabolic diseases)	45-54	10.9	10	0.3675
State of Mexico	Huixquilucan	Chapter 4 (Endocrine, nutritional and metabolic diseases)	55-64	15.9	10	0.1024
State of Mexico	Huixquilucan	Chapter 4 (Endocrine, nutritional and metabolic diseases)	65+	5.8	10	0.8335
State of Mexico	Huixquilucan	Chapter 4 (Endocrine, nutritional and metabolic diseases)	0-4	3.3	10	0.9737
State of Mexico	Huixquilucan	Chapter 4 (Endocrine, nutritional and metabolic diseases)	15-24	5.2	4	0.2658
State of Mexico	Huixquilucan	Chapter 6 (Diseases of the nervous system)	5-14	1.4	9	0.9980
State of Mexico	Huixquilucan	Chapter 6 (Diseases of the nervous system)	35-44	9.6	9	0.3815
State of Mexico	Huixquilucan	Chapter 6 (Diseases of the nervous system)	15-24	6.1	7	0.5307
State of Mexico	Huixquilucan	Chapter 6 (Diseases of the nervous system)	65+	18.4	10	0.0492
State of Mexico	Huixquilucan	Chapter 6 (Diseases of the nervous system)	0-4	8.6	10	0.5680
State of Mexico	Huixquilucan	Chapter 6 (Diseases of the nervous system)	55-64	16.1	7	**0.0240***
State of Mexico	Huixquilucan	Chapter 6 (Diseases of the nervous system)	45-54	8.8	8	0.3574
State of Mexico	Huixquilucan	Chapter 6 (Diseases of the nervous system)	25-34	4.9	7	0.6670
State of Mexico	Huixquilucan	Chapter 10 (Diseases of the respiratory system)	0-4	8.9	10	0.5451

State of Mexico	Huixquilucan	Chapter 10 (Diseases of the respiratory system)	35-44	8.2	10	0.6139
State of Mexico	Huixquilucan	Chapter 10 (Diseases of the respiratory system)	45-54	3.5	8	0.9016
State of Mexico	Huixquilucan	Chapter 10 (Diseases of the respiratory system)	55-64	8.7	10	0.5643
State of Mexico	Huixquilucan	Chapter 10 (Diseases of the respiratory system)	65+	12.2	10	0.2693
State of Mexico	Huixquilucan	Chapter 10 (Diseases of the respiratory system)	15-24	7.3	5	0.2000
State of Mexico	Huixquilucan	Chapter 10 (Diseases of the respiratory system)	25-34	7.3	7	0.3973
State of Mexico	Huixquilucan	Chapter 5 (Mental and behavioural disorders)	45-54	10.8	10	0.3728
State of Mexico	Huixquilucan	Chapter 5 (Mental and behavioural disorders)	65+	10.4	10	0.4039
State of Mexico	Huixquilucan	Chapter 5 (Mental and behavioural disorders)	25-34	6.8	5	0.2370
State of Mexico	Huixquilucan	Chapter 5 (Mental and behavioural disorders)	55-64	6.4	4	0.1730
State of Mexico	Isidro Fabela	Chapter 9 (Diseases of the circulatory system)	65+	14.2	10	0.1646
State of Mexico	Isidro Fabela	Chapter 4 (Endocrine, nutritional and metabolic diseases)	45-54	1.4	7	0.9845
State of Mexico	Isidro Fabela	Chapter 4 (Endocrine, nutritional and metabolic diseases)	65+	11.6	10	0.3161
State of Mexico	Isidro Fabela	Chapter 4 (Endocrine, nutritional and metabolic diseases)	55-64	13.7	10	0.1888
State of Mexico	Isidro Fabela	Chapter 10 (Diseases of the respiratory system)	0-4	12.5	6	0.0509
State of Mexico	Isidro Fabela	Chapter 10 (Diseases of the respiratory system)	65+	6.0	10	0.8185
State of Mexico	Isidro Fabela	Chapter 10 (Diseases of the respiratory system)	55-64	4.0	4	0.4120
State of Mexico	Ixtapaluca	Chapter 9 (Diseases of the circulatory system)	35-44	5.5	10	0.8559
State of Mexico	Ixtapaluca	Chapter 9 (Diseases of the circulatory system)	45-54	6.1	10	0.8040
State of Mexico	Ixtapaluca	Chapter 9 (Diseases of the circulatory system)	55-64	13.5	10	0.1987
State of Mexico	Ixtapaluca	Chapter 9 (Diseases of the circulatory system)	65+	7.5	10	0.6776
State of Mexico	Ixtapaluca	Chapter 9 (Diseases of the circulatory system)	15-24	9.7	10	0.4636
State of Mexico	Ixtapaluca	Chapter 9 (Diseases of the circulatory system)	5-14	5.1	6	0.5356
State of Mexico	Ixtapaluca	Chapter 9 (Diseases of the circulatory system)	25-34	11.5	10	0.3196
State of Mexico	Ixtapaluca	Chapter 9 (Diseases of the circulatory system)	0-4	3.4	7	0.8494
State of Mexico	Ixtapaluca	Chapter 4 (Endocrine, nutritional and metabolic diseases)	0-4	6.4	10	0.7801
State of Mexico	Ixtapaluca	Chapter 4 (Endocrine, nutritional and metabolic diseases)	45-54	10.5	10	0.3938
State of Mexico	Ixtapaluca	Chapter 4 (Endocrine, nutritional and metabolic diseases)	55-64	10.8	10	0.3707
State of Mexico	Ixtapaluca	Chapter 4 (Endocrine, nutritional and metabolic diseases)	65+	19.5	10	**0.0348***
State of Mexico	Ixtapaluca	Chapter 4 (Endocrine, nutritional and metabolic diseases)	5-14	2.0	5	0.8516
State of Mexico	Ixtapaluca	Chapter 4 (Endocrine, nutritional and metabolic diseases)	15-24	17.6	10	0.0625
State of Mexico	Ixtapaluca	Chapter 4 (Endocrine, nutritional and metabolic diseases)	25-34	6.4	10	0.7840
State of Mexico	Ixtapaluca	Chapter 4 (Endocrine, nutritional and metabolic diseases)	35-44	6.4	10	0.7834
State of Mexico	Ixtapaluca	Chapter 6 (Diseases of the nervous system)	0-4	10.7	10	0.3842
State of Mexico	Ixtapaluca	Chapter 6 (Diseases of the nervous system)	5-14	10.7	10	0.3779
State of Mexico	Ixtapaluca	Chapter 6 (Diseases of the nervous system)	25-34	7.7	10	0.6616
State of Mexico	Ixtapaluca	Chapter 6 (Diseases of the nervous system)	35-44	10.0	10	0.4362
State of Mexico	Ixtapaluca	Chapter 6 (Diseases of the nervous system)	45-54	27.8	10	**0.0019***
State of Mexico	Ixtapaluca	Chapter 6 (Diseases of the nervous system)	65+	12.9	10	0.2281
State of Mexico	Ixtapaluca	Chapter 6 (Diseases of the nervous system)	15-24	13.6	10	0.1906
State of Mexico	Ixtapaluca	Chapter 6 (Diseases of the nervous system)	55-64	8.3	9	0.5007
State of Mexico	Ixtapaluca	Chapter 10 (Diseases of the respiratory system)	0-4	13.9	10	0.1776
State of Mexico	Ixtapaluca	Chapter 10 (Diseases of the respiratory system)	45-54	7.0	10	0.7268

State of Mexico	Ixtapaluca	Chapter 10 (Diseases of the respiratory system)	55-64	11.4	10	0.3260
State of Mexico	Ixtapaluca	Chapter 10 (Diseases of the respiratory system)	65+	5.4	10	0.8635
State of Mexico	Ixtapaluca	Chapter 10 (Diseases of the respiratory system)	5-14	11.1	9	0.2667
State of Mexico	Ixtapaluca	Chapter 10 (Diseases of the respiratory system)	15-24	3.5	9	0.9386
State of Mexico	Ixtapaluca	Chapter 10 (Diseases of the respiratory system)	25-34	11.3	10	0.3371
State of Mexico	Ixtapaluca	Chapter 10 (Diseases of the respiratory system)	35-44	14.8	10	0.1392
State of Mexico	Ixtapaluca	Chapter 5 (Mental and behavioural disorders)	55-64	14.6	10	0.1458
State of Mexico	Ixtapaluca	Chapter 5 (Mental and behavioural disorders)	15-24	14.1	7	0.0502
State of Mexico	Ixtapaluca	Chapter 5 (Mental and behavioural disorders)	35-44	27.8	10	**0.0020***
State of Mexico	Ixtapaluca	Chapter 5 (Mental and behavioural disorders)	45-54	6.7	10	0.7504
State of Mexico	Ixtapaluca	Chapter 5 (Mental and behavioural disorders)	65+	13.8	10	0.1830
State of Mexico	Ixtapaluca	Chapter 5 (Mental and behavioural disorders)	25-34	4.1	4	0.3950
State of Mexico	Jaltenco	Chapter 9 (Diseases of the circulatory system)	35-44	8.1	6	0.2323
State of Mexico	Jaltenco	Chapter 9 (Diseases of the circulatory system)	45-54	6.4	10	0.7777
State of Mexico	Jaltenco	Chapter 9 (Diseases of the circulatory system)	55-64	12.2	10	0.2701
State of Mexico	Jaltenco	Chapter 9 (Diseases of the circulatory system)	65+	8.0	10	0.6294
State of Mexico	Jaltenco	Chapter 4 (Endocrine, nutritional and metabolic diseases)	45-54	9.6	7	0.2115
State of Mexico	Jaltenco	Chapter 4 (Endocrine, nutritional and metabolic diseases)	55-64	10.9	10	0.3634
State of Mexico	Jaltenco	Chapter 4 (Endocrine, nutritional and metabolic diseases)	65+	20.7	10	**0.0233***
State of Mexico	Jaltenco	Chapter 6 (Diseases of the nervous system)	0-4	3.7	4	0.4486
State of Mexico	Jaltenco	Chapter 10 (Diseases of the respiratory system)	0-4	14.8	10	0.1385
State of Mexico	Jaltenco	Chapter 10 (Diseases of the respiratory system)	65+	16.0	10	0.1010
State of Mexico	Jaltenco	Chapter 10 (Diseases of the respiratory system)	45-54	3.9	4	0.4262
State of Mexico	Jaltenco	Chapter 10 (Diseases of the respiratory system)	55-64	6.4	4	0.1741
State of Mexico	Jilotzingo	Chapter 9 (Diseases of the circulatory system)	55-64	13.4	10	0.2007
State of Mexico	Jilotzingo	Chapter 9 (Diseases of the circulatory system)	65+	8.1	10	0.6195
State of Mexico	Jilotzingo	Chapter 9 (Diseases of the circulatory system)	45-54	2.2	4	0.7018
State of Mexico	Jilotzingo	Chapter 4 (Endocrine, nutritional and metabolic diseases)	45-54	9.6	9	0.3836
State of Mexico	Jilotzingo	Chapter 4 (Endocrine, nutritional and metabolic diseases)	55-64	8.0	10	0.6336
State of Mexico	Jilotzingo	Chapter 4 (Endocrine, nutritional and metabolic diseases)	65+	10.0	10	0.4373
State of Mexico	Jilotzingo	Chapter 6 (Diseases of the nervous system)	65+	6.2	5	0.2887
State of Mexico	Jilotzingo	Chapter 10 (Diseases of the respiratory system)	0-4	6.2	8	0.6278
State of Mexico	Jilotzingo	Chapter 10 (Diseases of the respiratory system)	65+	8.5	10	0.5818
State of Mexico	Jilotzingo	Chapter 10 (Diseases of the respiratory system)	55-64	2.1	4	0.7143
State of Mexico	Juchitepec	Chapter 9 (Diseases of the circulatory system)	65+	12.2	10	0.2719
State of Mexico	Juchitepec	Chapter 9 (Diseases of the circulatory system)	45-54	6.8	10	0.7439
State of Mexico	Juchitepec	Chapter 9 (Diseases of the circulatory system)	55-64	19.0	10	**0.0399***
State of Mexico	Juchitepec	Chapter 9 (Diseases of the circulatory system)	35-44	9.6	9	0.3807
State of Mexico	Juchitepec	Chapter 4 (Endocrine, nutritional and metabolic diseases)	45-54	10.2	10	0.4253
State of Mexico	Juchitepec	Chapter 4 (Endocrine, nutritional and metabolic diseases)	55-64	9.8	10	0.4581
State of Mexico	Juchitepec	Chapter 4 (Endocrine, nutritional and metabolic diseases)	65+	5.3	10	0.8729
State of Mexico	Juchitepec	Chapter 4 (Endocrine, nutritional and metabolic diseases)	35-44	11.3	10	0.3326
State of Mexico	Juchitepec	Chapter 6 (Diseases of the nervous system)	65+	4.3	5	0.5092

State of Mexico	Juchitepec	Chapter 10 (Diseases of the respiratory system)	0-4	13.4	10	0.2014
State of Mexico	Juchitepec	Chapter 10 (Diseases of the respiratory system)	65+	9.5	10	0.4887
State of Mexico	Juchitepec	Chapter 10 (Diseases of the respiratory system)	55-64	2.2	5	0.8162
State of Mexico	Melchor Ocampo	Chapter 9 (Diseases of the circulatory system)	45-54	7.1	10	0.7121
State of Mexico	Melchor Ocampo	Chapter 9 (Diseases of the circulatory system)	55-64	15.0	10	0.1318
State of Mexico	Melchor Ocampo	Chapter 9 (Diseases of the circulatory system)	65+	8.7	10	0.5643
State of Mexico	Melchor Ocampo	Chapter 9 (Diseases of the circulatory system)	35-44	4.3	7	0.7441
State of Mexico	Melchor Ocampo	Chapter 9 (Diseases of the circulatory system)	25-34	2.9	10	0.9845
State of Mexico	Melchor Ocampo	Chapter 4 (Endocrine, nutritional and metabolic diseases)	35-44	10.5	10	0.4011
State of Mexico	Melchor Ocampo	Chapter 4 (Endocrine, nutritional and metabolic diseases)	45-54	8.5	10	0.5812
State of Mexico	Melchor Ocampo	Chapter 4 (Endocrine, nutritional and metabolic diseases)	55-64	4.9	10	0.9002
State of Mexico	Melchor Ocampo	Chapter 4 (Endocrine, nutritional and metabolic diseases)	65+	6.8	10	0.7401
State of Mexico	Melchor Ocampo	Chapter 4 (Endocrine, nutritional and metabolic diseases)	0-4	6.8	4	0.1483
State of Mexico	Melchor Ocampo	Chapter 4 (Endocrine, nutritional and metabolic diseases)	25-34	3.5	4	0.4787
State of Mexico	Melchor Ocampo	Chapter 6 (Diseases of the nervous system)	65+	17.7	10	0.0608
State of Mexico	Melchor Ocampo	Chapter 6 (Diseases of the nervous system)	15-24	6.2	6	0.4051
State of Mexico	Melchor Ocampo	Chapter 6 (Diseases of the nervous system)	45-54	4.8	6	0.5653
State of Mexico	Melchor Ocampo	Chapter 10 (Diseases of the respiratory system)	0-4	7.6	10	0.6641
State of Mexico	Melchor Ocampo	Chapter 10 (Diseases of the respiratory system)	65+	8.2	10	0.6092
State of Mexico	Melchor Ocampo	Chapter 10 (Diseases of the respiratory system)	55-64	13.5	7	0.0610
State of Mexico	Naucalpan de Juárez	Chapter 9 (Diseases of the circulatory system)	0-4	28.3	10	**0.0016**
State of Mexico	Naucalpan de Juárez	Chapter 9 (Diseases of the circulatory system)	5-14	8.9	8	0.3543
State of Mexico	Naucalpan de Juárez	Chapter 9 (Diseases of the circulatory system)	15-24	7.8	10	0.6495
State of Mexico	Naucalpan de Juárez	Chapter 9 (Diseases of the circulatory system)	25-34	6.5	10	0.7746
State of Mexico	Naucalpan de Juárez	Chapter 9 (Diseases of the circulatory system)	35-44	6.8	10	0.7472
State of Mexico	Naucalpan de Juárez	Chapter 9 (Diseases of the circulatory system)	45-54	16.3	10	0.0912
State of Mexico	Naucalpan de Juárez	Chapter 9 (Diseases of the circulatory system)	55-64	9.9	10	0.4489
State of Mexico	Naucalpan de Juárez	Chapter 9 (Diseases of the circulatory system)	65+	8.7	10	0.5575
State of Mexico	Naucalpan de Juárez	Chapter 4 (Endocrine, nutritional and metabolic diseases)	0-4	8.6	10	0.5717
State of Mexico	Naucalpan de Juárez	Chapter 4 (Endocrine, nutritional and metabolic diseases)	5-14	10.2	10	0.4244
State of Mexico	Naucalpan de Juárez	Chapter 4 (Endocrine, nutritional and metabolic diseases)	15-24	4.0	10	0.9493
State of Mexico	Naucalpan de Juárez	Chapter 4 (Endocrine, nutritional and metabolic diseases)	25-34	22.6	10	**0.0122***
State of Mexico	Naucalpan de Juárez	Chapter 4 (Endocrine, nutritional and metabolic diseases)	35-44	6.2	10	0.8010
State of Mexico	Naucalpan de Juárez	Chapter 4 (Endocrine, nutritional and metabolic diseases)	45-54	11.4	10	0.3304
State of Mexico	Naucalpan de Juárez	Chapter 4 (Endocrine, nutritional and metabolic diseases)	55-64	9.5	10	0.4827
State of Mexico	Naucalpan de Juárez	Chapter 4 (Endocrine, nutritional and metabolic diseases)	65+	7.7	10	0.6574
State of Mexico	Naucalpan de Juárez	Chapter 6 (Diseases of the nervous system)	0-4	7.1	10	0.7130
State of Mexico	Naucalpan de Juárez	Chapter 6 (Diseases of the nervous system)	5-14	10.2	10	0.4249
State of Mexico	Naucalpan de Juárez	Chapter 6 (Diseases of the nervous system)	15-24	8.5	10	0.5754
State of Mexico	Naucalpan de Juárez	Chapter 6 (Diseases of the nervous system)	25-34	17.6	10	0.0626
State of Mexico	Naucalpan de Juárez	Chapter 6 (Diseases of the nervous system)	35-44	12.3	10	0.2637
State of Mexico	Naucalpan de Juárez	Chapter 6 (Diseases of the nervous system)	45-54	7.9	10	0.6377
State of Mexico	Naucalpan de Juárez	Chapter 6 (Diseases of the nervous system)	55-64	3.3	10	0.9726

State of Mexico	Naucalpan de Juárez	Chapter 6 (Diseases of the nervous system)	65+	5.9	10	0.8199
State of Mexico	Naucalpan de Juárez	Chapter 10 (Diseases of the respiratory system)	0-4	18.4	10	0.0487
State of Mexico	Naucalpan de Juárez	Chapter 10 (Diseases of the respiratory system)	5-14	19.3	10	**0.0368***
State of Mexico	Naucalpan de Juárez	Chapter 10 (Diseases of the respiratory system)	15-24	12.9	10	0.2299
State of Mexico	Naucalpan de Juárez	Chapter 10 (Diseases of the respiratory system)	25-34	13.3	10	0.2100
State of Mexico	Naucalpan de Juárez	Chapter 10 (Diseases of the respiratory system)	35-44	6.6	10	0.7664
State of Mexico	Naucalpan de Juárez	Chapter 10 (Diseases of the respiratory system)	45-54	8.1	10	0.6181
State of Mexico	Naucalpan de Juárez	Chapter 10 (Diseases of the respiratory system)	55-64	13.6	10	0.1941
State of Mexico	Naucalpan de Juárez	Chapter 10 (Diseases of the respiratory system)	65+	7.3	10	0.7015
State of Mexico	Naucalpan de Juárez	Chapter 5 (Mental and behavioural disorders)	15-24	4.9	7	0.6716
State of Mexico	Naucalpan de Juárez	Chapter 5 (Mental and behavioural disorders)	25-34	11.2	10	0.3454
State of Mexico	Naucalpan de Juárez	Chapter 5 (Mental and behavioural disorders)	35-44	11.0	10	0.3588
State of Mexico	Naucalpan de Juárez	Chapter 5 (Mental and behavioural disorders)	45-54	9.8	10	0.4623
State of Mexico	Naucalpan de Juárez	Chapter 5 (Mental and behavioural disorders)	55-64	14.4	10	0.1575
State of Mexico	Naucalpan de Juárez	Chapter 5 (Mental and behavioural disorders)	65+	10.3	10	0.4115
State of Mexico	Nezahualcóyotl	Chapter 9 (Diseases of the circulatory system)	0-4	11.8	10	0.3010
State of Mexico	Nezahualcóyotl	Chapter 9 (Diseases of the circulatory system)	15-24	7.8	10	0.6469
State of Mexico	Nezahualcóyotl	Chapter 9 (Diseases of the circulatory system)	25-34	14.3	10	0.1610
State of Mexico	Nezahualcóyotl	Chapter 9 (Diseases of the circulatory system)	35-44	10.2	10	0.4196
State of Mexico	Nezahualcóyotl	Chapter 9 (Diseases of the circulatory system)	45-54	43.8	10	**0.0000***
State of Mexico	Nezahualcóyotl	Chapter 9 (Diseases of the circulatory system)	55-64	7.2	10	0.7046
State of Mexico	Nezahualcóyotl	Chapter 9 (Diseases of the circulatory system)	65+	4.1	10	0.9421
State of Mexico	Nezahualcóyotl	Chapter 9 (Diseases of the circulatory system)	5-14	4.9	6	0.5587
State of Mexico	Nezahualcóyotl	Chapter 4 (Endocrine, nutritional and metabolic diseases)	0-4	30.6	10	**0.0007***
State of Mexico	Nezahualcóyotl	Chapter 4 (Endocrine, nutritional and metabolic diseases)	5-14	10.7	10	0.3837
State of Mexico	Nezahualcóyotl	Chapter 4 (Endocrine, nutritional and metabolic diseases)	15-24	9.6	10	0.4733
State of Mexico	Nezahualcóyotl	Chapter 4 (Endocrine, nutritional and metabolic diseases)	25-34	9.9	10	0.4530
State of Mexico	Nezahualcóyotl	Chapter 4 (Endocrine, nutritional and metabolic diseases)	35-44	32.2	10	**0.0004***
State of Mexico	Nezahualcóyotl	Chapter 4 (Endocrine, nutritional and metabolic diseases)	45-54	11.0	10	0.3571
State of Mexico	Nezahualcóyotl	Chapter 4 (Endocrine, nutritional and metabolic diseases)	55-64	11.2	10	0.3454
State of Mexico	Nezahualcóyotl	Chapter 4 (Endocrine, nutritional and metabolic diseases)	65+	5.0	10	0.8909
State of Mexico	Nezahualcóyotl	Chapter 6 (Diseases of the nervous system)	0-4	22.7	10	**0.0120***
State of Mexico	Nezahualcóyotl	Chapter 6 (Diseases of the nervous system)	5-14	5.6	10	0.8476
State of Mexico	Nezahualcóyotl	Chapter 6 (Diseases of the nervous system)	15-24	9.8	10	0.4565
State of Mexico	Nezahualcóyotl	Chapter 6 (Diseases of the nervous system)	25-34	18.7	10	**0.0442***
State of Mexico	Nezahualcóyotl	Chapter 6 (Diseases of the nervous system)	35-44	10.0	10	0.4406
State of Mexico	Nezahualcóyotl	Chapter 6 (Diseases of the nervous system)	45-54	21.3	10	**0.0188***
State of Mexico	Nezahualcóyotl	Chapter 6 (Diseases of the nervous system)	55-64	9.4	10	0.4974
State of Mexico	Nezahualcóyotl	Chapter 6 (Diseases of the nervous system)	65+	10.9	10	0.3661
State of Mexico	Nezahualcóyotl	Chapter 10 (Diseases of the respiratory system)	0-4	10.7	10	0.3819
State of Mexico	Nezahualcóyotl	Chapter 10 (Diseases of the respiratory system)	5-14	4.5	10	0.9246
State of Mexico	Nezahualcóyotl	Chapter 10 (Diseases of the respiratory system)	15-24	12.8	10	0.2328
State of Mexico	Nezahualcóyotl	Chapter 10 (Diseases of the respiratory system)	25-34	5.2	10	0.8772

State of Mexico	Nezahualcóyotl	Chapter 10 (Diseases of the respiratory system)	35-44	10.5	10	0.3973
State of Mexico	Nezahualcóyotl	Chapter 10 (Diseases of the respiratory system)	45-54	15.4	10	0.1188
State of Mexico	Nezahualcóyotl	Chapter 10 (Diseases of the respiratory system)	55-64	4.8	10	0.9060
State of Mexico	Nezahualcóyotl	Chapter 10 (Diseases of the respiratory system)	65+	21.0	10	**0.0213***
State of Mexico	Nezahualcóyotl	Chapter 5 (Mental and behavioural disorders)	25-34	9.5	10	0.4869
State of Mexico	Nezahualcóyotl	Chapter 5 (Mental and behavioural disorders)	35-44	7.9	10	0.6341
State of Mexico	Nezahualcóyotl	Chapter 5 (Mental and behavioural disorders)	45-54	9.4	10	0.4928
State of Mexico	Nezahualcóyotl	Chapter 5 (Mental and behavioural disorders)	55-64	18.4	10	0.0491
State of Mexico	Nezahualcóyotl	Chapter 5 (Mental and behavioural disorders)	65+	11.3	10	0.3341
State of Mexico	Nezahualcóyotl	Chapter 5 (Mental and behavioural disorders)	15-24	7.8	7	0.3487
State of Mexico	Nextlalpan	Chapter 9 (Diseases of the circulatory system)	65+	14.5	10	0.1515
State of Mexico	Nextlalpan	Chapter 9 (Diseases of the circulatory system)	45-54	7.7	10	0.6578
State of Mexico	Nextlalpan	Chapter 9 (Diseases of the circulatory system)	35-44	3.4	4	0.4959
State of Mexico	Nextlalpan	Chapter 9 (Diseases of the circulatory system)	55-64	12.8	10	0.2344
State of Mexico	Nextlalpan	Chapter 4 (Endocrine, nutritional and metabolic diseases)	55-64	7.7	10	0.6550
State of Mexico	Nextlalpan	Chapter 4 (Endocrine, nutritional and metabolic diseases)	65+	6.5	10	0.7725
State of Mexico	Nextlalpan	Chapter 4 (Endocrine, nutritional and metabolic diseases)	0-4	4.0	8	0.8605
State of Mexico	Nextlalpan	Chapter 4 (Endocrine, nutritional and metabolic diseases)	45-54	5.0	8	0.7547
State of Mexico	Nextlalpan	Chapter 4 (Endocrine, nutritional and metabolic diseases)	35-44	1.6	10	0.9987
State of Mexico	Nextlalpan	Chapter 10 (Diseases of the respiratory system)	0-4	10.2	10	0.4271
State of Mexico	Nextlalpan	Chapter 10 (Diseases of the respiratory system)	45-54	4.9	4	0.2969
State of Mexico	Nextlalpan	Chapter 10 (Diseases of the respiratory system)	65+	12.7	10	0.2403
State of Mexico	Nicolás Romero	Chapter 9 (Diseases of the circulatory system)	5-14	3.2	7	0.8671
State of Mexico	Nicolás Romero	Chapter 9 (Diseases of the circulatory system)	15-24	20.0	10	**0.0292***
State of Mexico	Nicolás Romero	Chapter 9 (Diseases of the circulatory system)	25-34	6.7	10	0.7563
State of Mexico	Nicolás Romero	Chapter 9 (Diseases of the circulatory system)	35-44	10.2	10	0.4224
State of Mexico	Nicolás Romero	Chapter 9 (Diseases of the circulatory system)	45-54	6.0	10	0.8183
State of Mexico	Nicolás Romero	Chapter 9 (Diseases of the circulatory system)	55-64	3.9	10	0.9521
State of Mexico	Nicolás Romero	Chapter 9 (Diseases of the circulatory system)	65+	13.0	10	0.2258
State of Mexico	Nicolás Romero	Chapter 4 (Endocrine, nutritional and metabolic diseases)	0-4	9.2	10	0.5157
State of Mexico	Nicolás Romero	Chapter 4 (Endocrine, nutritional and metabolic diseases)	5-14	4.4	5	0.4880
State of Mexico	Nicolás Romero	Chapter 4 (Endocrine, nutritional and metabolic diseases)	15-24	11.2	9	0.2608
State of Mexico	Nicolás Romero	Chapter 4 (Endocrine, nutritional and metabolic diseases)	25-34	18.8	10	**0.0434***
State of Mexico	Nicolás Romero	Chapter 4 (Endocrine, nutritional and metabolic diseases)	35-44	7.2	10	0.7082
State of Mexico	Nicolás Romero	Chapter 4 (Endocrine, nutritional and metabolic diseases)	45-54	13.9	10	0.1775
State of Mexico	Nicolás Romero	Chapter 4 (Endocrine, nutritional and metabolic diseases)	55-64	18.5	10	**0.0464***
State of Mexico	Nicolás Romero	Chapter 4 (Endocrine, nutritional and metabolic diseases)	65+	5.0	10	0.8921
State of Mexico	Nicolás Romero	Chapter 6 (Diseases of the nervous system)	0-4	15.0	10	0.1306
State of Mexico	Nicolás Romero	Chapter 6 (Diseases of the nervous system)	25-34	7.0	10	0.7268
State of Mexico	Nicolás Romero	Chapter 6 (Diseases of the nervous system)	35-44	14.9	10	0.1343
State of Mexico	Nicolás Romero	Chapter 6 (Diseases of the nervous system)	65+	25.7	10	**0.0042***
State of Mexico	Nicolás Romero	Chapter 6 (Diseases of the nervous system)	5-14	8.3	10	0.5964
State of Mexico	Nicolás Romero	Chapter 6 (Diseases of the nervous system)	15-24	5.0	10	0.8923

State of Mexico	Nicolás Romero	Chapter 6 (Diseases of the nervous system)	55-64	17.5	10	0.0643
State of Mexico	Nicolás Romero	Chapter 6 (Diseases of the nervous system)	45-54	9.2	10	0.5128
State of Mexico	Nicolás Romero	Chapter 10 (Diseases of the respiratory system)	0-4	14.0	10	0.1708
State of Mexico	Nicolás Romero	Chapter 10 (Diseases of the respiratory system)	5-14	5.7	6	0.4555
State of Mexico	Nicolás Romero	Chapter 10 (Diseases of the respiratory system)	25-34	12.8	10	0.2353
State of Mexico	Nicolás Romero	Chapter 10 (Diseases of the respiratory system)	35-44	23.1	10	**0.0104***
State of Mexico	Nicolás Romero	Chapter 10 (Diseases of the respiratory system)	45-54	10.1	10	0.4347
State of Mexico	Nicolás Romero	Chapter 10 (Diseases of the respiratory system)	55-64	9.7	10	0.4697
State of Mexico	Nicolás Romero	Chapter 10 (Diseases of the respiratory system)	65+	7.5	10	0.6775
State of Mexico	Nicolás Romero	Chapter 10 (Diseases of the respiratory system)	15-24	13.2	8	0.1047
State of Mexico	Nicolás Romero	Chapter 5 (Mental and behavioural disorders)	25-34	6.1	7	0.5333
State of Mexico	Nicolás Romero	Chapter 5 (Mental and behavioural disorders)	35-44	15.3	10	0.1221
State of Mexico	Nicolás Romero	Chapter 5 (Mental and behavioural disorders)	45-54	9.9	10	0.4471
State of Mexico	Nicolás Romero	Chapter 5 (Mental and behavioural disorders)	65+	21.2	10	**0.0195***
State of Mexico	Nicolás Romero	Chapter 5 (Mental and behavioural disorders)	55-64	7.8	10	0.6518
State of Mexico	Nopaltepec	Chapter 9 (Diseases of the circulatory system)	55-64	25.5	9	**0.0024***
State of Mexico	Nopaltepec	Chapter 9 (Diseases of the circulatory system)	65+	12.5	10	0.2517
State of Mexico	Nopaltepec	Chapter 4 (Endocrine, nutritional and metabolic diseases)	65+	21.1	10	**0.0204***
State of Mexico	Nopaltepec	Chapter 4 (Endocrine, nutritional and metabolic diseases)	55-64	1.8	6	0.9403
State of Mexico	Nopaltepec	Chapter 4 (Endocrine, nutritional and metabolic diseases)	45-54	1.6	7	0.9797
State of Mexico	Nopaltepec	Chapter 10 (Diseases of the respiratory system)	65+	14.6	10	0.1475
State of Mexico	Nopaltepec	Chapter 10 (Diseases of the respiratory system)	0-4	6.8	6	0.3353
State of Mexico	Otumba	Chapter 9 (Diseases of the circulatory system)	35-44	8.0	10	0.6272
State of Mexico	Otumba	Chapter 9 (Diseases of the circulatory system)	65+	4.4	10	0.9271
State of Mexico	Otumba	Chapter 9 (Diseases of the circulatory system)	45-54	7.9	10	0.6402
State of Mexico	Otumba	Chapter 9 (Diseases of the circulatory system)	25-34	4.4	6	0.6286
State of Mexico	Otumba	Chapter 9 (Diseases of the circulatory system)	55-64	7.5	10	0.6780
State of Mexico	Otumba	Chapter 4 (Endocrine, nutritional and metabolic diseases)	25-34	3.7	4	0.4529
State of Mexico	Otumba	Chapter 4 (Endocrine, nutritional and metabolic diseases)	55-64	12.1	10	0.2798
State of Mexico	Otumba	Chapter 4 (Endocrine, nutritional and metabolic diseases)	65+	7.7	10	0.6539
State of Mexico	Otumba	Chapter 4 (Endocrine, nutritional and metabolic diseases)	35-44	2.3	9	0.9854
State of Mexico	Otumba	Chapter 4 (Endocrine, nutritional and metabolic diseases)	45-54	17.4	10	0.0666
State of Mexico	Otumba	Chapter 6 (Diseases of the nervous system)	65+	4.1	4	0.3962
State of Mexico	Otumba	Chapter 10 (Diseases of the respiratory system)	0-4	10.1	10	0.4328
State of Mexico	Otumba	Chapter 10 (Diseases of the respiratory system)	5-14	1.9	4	0.7455
State of Mexico	Otumba	Chapter 10 (Diseases of the respiratory system)	65+	23.3	10	**0.0097***
State of Mexico	Otumba	Chapter 10 (Diseases of the respiratory system)	25-34	2.5	4	0.6460
State of Mexico	Otumba	Chapter 10 (Diseases of the respiratory system)	55-64	4.1	5	0.5295
State of Mexico	Otumba	Chapter 5 (Mental and behavioural disorders)	65+	9.1	7	0.2435
State of Mexico	Ozumba	Chapter 9 (Diseases of the circulatory system)	45-54	12.9	10	0.2316
State of Mexico	Ozumba	Chapter 9 (Diseases of the circulatory system)	55-64	5.9	10	0.8197
State of Mexico	Ozumba	Chapter 9 (Diseases of the circulatory system)	65+	5.4	10	0.8609
State of Mexico	Ozumba	Chapter 9 (Diseases of the circulatory system)	35-44	9.3	8	0.3149

State of Mexico	Ozumba	Chapter 4 (Endocrine, nutritional and metabolic diseases)	55-64	11.9	10	0.2922
State of Mexico	Ozumba	Chapter 4 (Endocrine, nutritional and metabolic diseases)	65+	13.5	10	0.1989
State of Mexico	Ozumba	Chapter 4 (Endocrine, nutritional and metabolic diseases)	35-44	9.4	7	0.2239
State of Mexico	Ozumba	Chapter 4 (Endocrine, nutritional and metabolic diseases)	45-54	4.1	10	0.9405
State of Mexico	Ozumba	Chapter 4 (Endocrine, nutritional and metabolic diseases)	25-34	1.6	7	0.9788
State of Mexico	Ozumba	Chapter 6 (Diseases of the nervous system)	65+	5.3	7	0.6269
State of Mexico	Ozumba	Chapter 10 (Diseases of the respiratory system)	0-4	11.6	10	0.3108
State of Mexico	Ozumba	Chapter 10 (Diseases of the respiratory system)	65+	12.8	10	0.2352
State of Mexico	Ozumba	Chapter 10 (Diseases of the respiratory system)	45-54	8.1	5	0.1483
State of Mexico	Ozumba	Chapter 10 (Diseases of the respiratory system)	55-64	5.3	6	0.5095
State of Mexico	Papalotla	Chapter 9 (Diseases of the circulatory system)	65+	3.2	10	0.9772
State of Mexico	Papalotla	Chapter 9 (Diseases of the circulatory system)	55-64	2.0	5	0.8536
State of Mexico	Papalotla	Chapter 4 (Endocrine, nutritional and metabolic diseases)	65+	4.3	10	0.9343
State of Mexico	Papalotla	Chapter 4 (Endocrine, nutritional and metabolic diseases)	55-64	8.1	6	0.2309
State of Mexico	Papalotla	Chapter 4 (Endocrine, nutritional and metabolic diseases)	45-54	3.7	5	0.5909
State of Mexico	Papalotla	Chapter 10 (Diseases of the respiratory system)	0-4	1.9	5	0.8626
State of Mexico	Papalotla	Chapter 10 (Diseases of the respiratory system)	65+	7.5	10	0.6749
State of Mexico	La Paz	Chapter 9 (Diseases of the circulatory system)	15-24	2.5	10	0.9908
State of Mexico	La Paz	Chapter 9 (Diseases of the circulatory system)	35-44	5.5	10	0.8542
State of Mexico	La Paz	Chapter 9 (Diseases of the circulatory system)	45-54	8.8	10	0.5497
State of Mexico	La Paz	Chapter 9 (Diseases of the circulatory system)	55-64	4.8	10	0.9037
State of Mexico	La Paz	Chapter 9 (Diseases of the circulatory system)	65+	5.9	10	0.8229
State of Mexico	La Paz	Chapter 9 (Diseases of the circulatory system)	0-4	3.1	4	0.5404
State of Mexico	La Paz	Chapter 9 (Diseases of the circulatory system)	25-34	9.9	10	0.4494
State of Mexico	La Paz	Chapter 4 (Endocrine, nutritional and metabolic diseases)	0-4	8.5	10	0.5786
State of Mexico	La Paz	Chapter 4 (Endocrine, nutritional and metabolic diseases)	15-24	4.1	6	0.6608
State of Mexico	La Paz	Chapter 4 (Endocrine, nutritional and metabolic diseases)	25-34	15.7	10	0.1100
State of Mexico	La Paz	Chapter 4 (Endocrine, nutritional and metabolic diseases)	35-44	6.2	10	0.7955
State of Mexico	La Paz	Chapter 4 (Endocrine, nutritional and metabolic diseases)	45-54	9.5	10	0.4854
State of Mexico	La Paz	Chapter 4 (Endocrine, nutritional and metabolic diseases)	55-64	10.5	10	0.3956
State of Mexico	La Paz	Chapter 4 (Endocrine, nutritional and metabolic diseases)	65+	6.2	10	0.7980
State of Mexico	La Paz	Chapter 6 (Diseases of the nervous system)	0-4	12.4	10	0.2610
State of Mexico	La Paz	Chapter 6 (Diseases of the nervous system)	15-24	7.2	10	0.7036
State of Mexico	La Paz	Chapter 6 (Diseases of the nervous system)	25-34	2.5	10	0.9904
State of Mexico	La Paz	Chapter 6 (Diseases of the nervous system)	45-54	9.1	10	0.5192
State of Mexico	La Paz	Chapter 6 (Diseases of the nervous system)	55-64	9.5	10	0.4887
State of Mexico	La Paz	Chapter 6 (Diseases of the nervous system)	65+	10.0	10	0.4399
State of Mexico	La Paz	Chapter 6 (Diseases of the nervous system)	5-14	10.1	8	0.2613
State of Mexico	La Paz	Chapter 6 (Diseases of the nervous system)	35-44	5.0	10	0.8878
State of Mexico	La Paz	Chapter 10 (Diseases of the respiratory system)	0-4	9.2	10	0.5169
State of Mexico	La Paz	Chapter 10 (Diseases of the respiratory system)	25-34	3.2	10	0.9752
State of Mexico	La Paz	Chapter 10 (Diseases of the respiratory system)	45-54	6.7	10	0.7505
State of Mexico	La Paz	Chapter 10 (Diseases of the respiratory system)	55-64	7.8	10	0.6453

State of Mexico	La Paz	Chapter 10 (Diseases of the respiratory system)	65+	14.0	10	0.1742
State of Mexico	La Paz	Chapter 10 (Diseases of the respiratory system)	5-14	10.1	6	0.1190
State of Mexico	La Paz	Chapter 10 (Diseases of the respiratory system)	35-44	4.0	9	0.9111
State of Mexico	La Paz	Chapter 5 (Mental and behavioural disorders)	25-34	6.2	7	0.5152
State of Mexico	La Paz	Chapter 5 (Mental and behavioural disorders)	45-54	9.1	10	0.5235
State of Mexico	La Paz	Chapter 5 (Mental and behavioural disorders)	65+	7.6	10	0.6726
State of Mexico	La Paz	Chapter 5 (Mental and behavioural disorders)	35-44	9.7	10	0.4691
State of Mexico	La Paz	Chapter 5 (Mental and behavioural disorders)	55-64	4.3	10	0.9350
State of Mexico	San Martín de las Pirámides	Chapter 9 (Diseases of the circulatory system)	55-64	14.8	10	0.1386
State of Mexico	San Martín de las Pirámides	Chapter 9 (Diseases of the circulatory system)	65+	19.4	10	**0.0354***
State of Mexico	San Martín de las Pirámides	Chapter 9 (Diseases of the circulatory system)	45-54	4.9	9	0.8433
State of Mexico	San Martín de las Pirámides	Chapter 9 (Diseases of the circulatory system)	35-44	4.7	5	0.4504
State of Mexico	San Martín de las Pirámides	Chapter 4 (Endocrine, nutritional and metabolic diseases)	55-64	14.4	10	0.1570
State of Mexico	San Martín de las Pirámides	Chapter 4 (Endocrine, nutritional and metabolic diseases)	65+	12.0	10	0.2818
State of Mexico	San Martín de las Pirámides	Chapter 4 (Endocrine, nutritional and metabolic diseases)	35-44	10.4	8	0.2387
State of Mexico	San Martín de las Pirámides	Chapter 4 (Endocrine, nutritional and metabolic diseases)	45-54	12.9	10	0.2292
State of Mexico	San Martín de las Pirámides	Chapter 6 (Diseases of the nervous system)	65+	2.4	5	0.7915
State of Mexico	San Martín de las Pirámides	Chapter 10 (Diseases of the respiratory system)	0-4	8.9	9	0.4446
State of Mexico	San Martín de las Pirámides	Chapter 10 (Diseases of the respiratory system)	55-64	4.6	4	0.3342
State of Mexico	San Martín de las Pirámides	Chapter 10 (Diseases of the respiratory system)	65+	8.8	10	0.5530
State of Mexico	Tecámac	Chapter 9 (Diseases of the circulatory system)	25-34	6.9	10	0.7307
State of Mexico	Tecámac	Chapter 9 (Diseases of the circulatory system)	35-44	10.8	10	0.3711
State of Mexico	Tecámac	Chapter 9 (Diseases of the circulatory system)	45-54	6.6	10	0.7629
State of Mexico	Tecámac	Chapter 9 (Diseases of the circulatory system)	55-64	13.7	10	0.1884
State of Mexico	Tecámac	Chapter 9 (Diseases of the circulatory system)	65+	10.1	10	0.4338
State of Mexico	Tecámac	Chapter 9 (Diseases of the circulatory system)	15-24	7.3	10	0.7016
State of Mexico	Tecámac	Chapter 4 (Endocrine, nutritional and metabolic diseases)	0-4	15.3	10	0.1231
State of Mexico	Tecámac	Chapter 4 (Endocrine, nutritional and metabolic diseases)	35-44	18.0	10	0.0543
State of Mexico	Tecámac	Chapter 4 (Endocrine, nutritional and metabolic diseases)	45-54	10.0	10	0.4374
State of Mexico	Tecámac	Chapter 4 (Endocrine, nutritional and metabolic diseases)	55-64	4.0	10	0.9484
State of Mexico	Tecámac	Chapter 4 (Endocrine, nutritional and metabolic diseases)	65+	7.3	10	0.6966
State of Mexico	Tecámac	Chapter 4 (Endocrine, nutritional and metabolic diseases)	15-24	2.2	8	0.9747
State of Mexico	Tecámac	Chapter 4 (Endocrine, nutritional and metabolic diseases)	25-34	10.8	10	0.3723
State of Mexico	Tecámac	Chapter 6 (Diseases of the nervous system)	0-4	10.9	10	0.3632
State of Mexico	Tecámac	Chapter 6 (Diseases of the nervous system)	15-24	3.5	10	0.9664
State of Mexico	Tecámac	Chapter 6 (Diseases of the nervous system)	65+	5.4	10	0.8603
State of Mexico	Tecámac	Chapter 6 (Diseases of the nervous system)	5-14	3.6	7	0.8205
State of Mexico	Tecámac	Chapter 6 (Diseases of the nervous system)	25-34	9.4	10	0.4918
State of Mexico	Tecámac	Chapter 6 (Diseases of the nervous system)	55-64	15.0	9	0.0912
State of Mexico	Tecámac	Chapter 6 (Diseases of the nervous system)	35-44	7.6	10	0.6662
State of Mexico	Tecámac	Chapter 6 (Diseases of the nervous system)	45-54	11.7	9	0.2326
State of Mexico	Tecámac	Chapter 10 (Diseases of the respiratory system)	0-4	13.0	10	0.2257
State of Mexico	Tecámac	Chapter 10 (Diseases of the respiratory system)	55-64	6.9	10	0.7335

State of Mexico	Tecámac	Chapter 10 (Diseases of the respiratory system)	65+	16.9	10	0.0776
State of Mexico	Tecámac	Chapter 10 (Diseases of the respiratory system)	5-14	5.1	5	0.4081
State of Mexico	Tecámac	Chapter 10 (Diseases of the respiratory system)	35-44	18.3	9	**0.0318***
State of Mexico	Tecámac	Chapter 10 (Diseases of the respiratory system)	15-24	15.6	10	0.1104
State of Mexico	Tecámac	Chapter 10 (Diseases of the respiratory system)	25-34	8.9	10	0.5380
State of Mexico	Tecámac	Chapter 10 (Diseases of the respiratory system)	45-54	7.8	10	0.6504
State of Mexico	Tecámac	Chapter 5 (Mental and behavioural disorders)	55-64	1.8	8	0.9870
State of Mexico	Tecámac	Chapter 5 (Mental and behavioural disorders)	65+	4.0	10	0.9463
State of Mexico	Tecámac	Chapter 5 (Mental and behavioural disorders)	35-44	11.4	7	0.1209
State of Mexico	Tecámac	Chapter 5 (Mental and behavioural disorders)	45-54	3.1	7	0.8774
State of Mexico	Temamatla	Chapter 9 (Diseases of the circulatory system)	55-64	7.2	10	0.7111
State of Mexico	Temamatla	Chapter 9 (Diseases of the circulatory system)	65+	3.7	10	0.9616
State of Mexico	Temamatla	Chapter 9 (Diseases of the circulatory system)	35-44	6.1	5	0.3008
State of Mexico	Temamatla	Chapter 9 (Diseases of the circulatory system)	45-54	7.1	5	0.2162
State of Mexico	Temamatla	Chapter 4 (Endocrine, nutritional and metabolic diseases)	65+	5.8	10	0.8341
State of Mexico	Temamatla	Chapter 4 (Endocrine, nutritional and metabolic diseases)	45-54	12.2	10	0.2689
State of Mexico	Temamatla	Chapter 4 (Endocrine, nutritional and metabolic diseases)	55-64	8.6	10	0.5731
State of Mexico	Temamatla	Chapter 10 (Diseases of the respiratory system)	0-4	8.4	8	0.3960
State of Mexico	Temamatla	Chapter 10 (Diseases of the respiratory system)	65+	37.7	10	**0.0000***
State of Mexico	Temascalapa	Chapter 9 (Diseases of the circulatory system)	65+	13.5	10	0.1965
State of Mexico	Temascalapa	Chapter 9 (Diseases of the circulatory system)	35-44	13.2	7	0.0671
State of Mexico	Temascalapa	Chapter 9 (Diseases of the circulatory system)	55-64	7.8	10	0.6447
State of Mexico	Temascalapa	Chapter 9 (Diseases of the circulatory system)	45-54	5.2	9	0.8134
State of Mexico	Temascalapa	Chapter 4 (Endocrine, nutritional and metabolic diseases)	55-64	21.6	10	**0.0172***
State of Mexico	Temascalapa	Chapter 4 (Endocrine, nutritional and metabolic diseases)	65+	6.5	10	0.7672
State of Mexico	Temascalapa	Chapter 4 (Endocrine, nutritional and metabolic diseases)	45-54	10.9	10	0.3649
State of Mexico	Temascalapa	Chapter 4 (Endocrine, nutritional and metabolic diseases)	35-44	7.6	9	0.5783
State of Mexico	Temascalapa	Chapter 6 (Diseases of the nervous system)	25-34	4.4	4	0.3595
State of Mexico	Temascalapa	Chapter 6 (Diseases of the nervous system)	65+	12.8	10	0.2327
State of Mexico	Temascalapa	Chapter 6 (Diseases of the nervous system)	35-44	1.8	5	0.8714
State of Mexico	Temascalapa	Chapter 6 (Diseases of the nervous system)	15-24	4.7	4	0.3232
State of Mexico	Temascalapa	Chapter 10 (Diseases of the respiratory system)	0-4	10.0	10	0.4419
State of Mexico	Temascalapa	Chapter 10 (Diseases of the respiratory system)	65+	6.5	10	0.7740
State of Mexico	Temascalapa	Chapter 10 (Diseases of the respiratory system)	55-64	6.2	5	0.2894
State of Mexico	Tenango del Aire	Chapter 9 (Diseases of the circulatory system)	65+	9.2	10	0.5093
State of Mexico	Tenango del Aire	Chapter 9 (Diseases of the circulatory system)	45-54	1.9	7	0.9673
State of Mexico	Tenango del Aire	Chapter 9 (Diseases of the circulatory system)	55-64	11.8	9	0.2266
State of Mexico	Tenango del Aire	Chapter 4 (Endocrine, nutritional and metabolic diseases)	45-54	8.7	10	0.5630
State of Mexico	Tenango del Aire	Chapter 4 (Endocrine, nutritional and metabolic diseases)	65+	12.3	10	0.2667
State of Mexico	Tenango del Aire	Chapter 4 (Endocrine, nutritional and metabolic diseases)	55-64	8.9	10	0.5436
State of Mexico	Tenango del Aire	Chapter 10 (Diseases of the respiratory system)	65+	6.9	10	0.7349
State of Mexico	Tenango del Aire	Chapter 10 (Diseases of the respiratory system)	0-4	9.8	7	0.2003
State of Mexico	Teoloyucan	Chapter 9 (Diseases of the circulatory system)	55-64	5.9	10	0.8273

State of Mexico	Teoloyucan	Chapter 9 (Diseases of the circulatory system)	65+	10.9	10	0.3670
State of Mexico	Teoloyucan	Chapter 9 (Diseases of the circulatory system)	15-24	12.3	7	0.0925
State of Mexico	Teoloyucan	Chapter 9 (Diseases of the circulatory system)	35-44	12.6	10	0.2485
State of Mexico	Teoloyucan	Chapter 9 (Diseases of the circulatory system)	45-54	7.6	10	0.6724
State of Mexico	Teoloyucan	Chapter 9 (Diseases of the circulatory system)	25-34	11.1	9	0.2702
State of Mexico	Teoloyucan	Chapter 4 (Endocrine, nutritional and metabolic diseases)	35-44	7.2	10	0.7039
State of Mexico	Teoloyucan	Chapter 4 (Endocrine, nutritional and metabolic diseases)	45-54	18.0	10	0.0548
State of Mexico	Teoloyucan	Chapter 4 (Endocrine, nutritional and metabolic diseases)	55-64	18.8	10	**0.0428***
State of Mexico	Teoloyucan	Chapter 4 (Endocrine, nutritional and metabolic diseases)	65+	7.0	10	0.7252
State of Mexico	Teoloyucan	Chapter 4 (Endocrine, nutritional and metabolic diseases)	0-4	5.2	4	0.2694
State of Mexico	Teoloyucan	Chapter 6 (Diseases of the nervous system)	5-14	8.1	6	0.2299
State of Mexico	Teoloyucan	Chapter 6 (Diseases of the nervous system)	65+	19.9	10	**0.0298***
State of Mexico	Teoloyucan	Chapter 6 (Diseases of the nervous system)	15-24	3.4	5	0.6406
State of Mexico	Teoloyucan	Chapter 10 (Diseases of the respiratory system)	0-4	12.1	10	0.2806
State of Mexico	Teoloyucan	Chapter 10 (Diseases of the respiratory system)	55-64	4.9	10	0.8976
State of Mexico	Teoloyucan	Chapter 10 (Diseases of the respiratory system)	65+	8.0	10	0.6277
State of Mexico	Teoloyucan	Chapter 10 (Diseases of the respiratory system)	45-54	4.2	4	0.3805
State of Mexico	Teoloyucan	Chapter 5 (Mental and behavioural disorders)	65+	10.5	8	0.2312
State of Mexico	Teotihuacán	Chapter 9 (Diseases of the circulatory system)	35-44	13.6	10	0.1924
State of Mexico	Teotihuacán	Chapter 9 (Diseases of the circulatory system)	45-54	7.3	10	0.6968
State of Mexico	Teotihuacán	Chapter 9 (Diseases of the circulatory system)	55-64	14.6	10	0.1463
State of Mexico	Teotihuacán	Chapter 9 (Diseases of the circulatory system)	65+	6.9	10	0.7363
State of Mexico	Teotihuacán	Chapter 4 (Endocrine, nutritional and metabolic diseases)	35-44	6.7	10	0.7510
State of Mexico	Teotihuacán	Chapter 4 (Endocrine, nutritional and metabolic diseases)	45-54	6.6	10	0.7611
State of Mexico	Teotihuacán	Chapter 4 (Endocrine, nutritional and metabolic diseases)	55-64	9.1	10	0.5240
State of Mexico	Teotihuacán	Chapter 4 (Endocrine, nutritional and metabolic diseases)	65+	11.8	10	0.3013
State of Mexico	Teotihuacán	Chapter 4 (Endocrine, nutritional and metabolic diseases)	25-34	17.0	9	0.0492
State of Mexico	Teotihuacán	Chapter 6 (Diseases of the nervous system)	65+	11.8	10	0.2982
State of Mexico	Teotihuacán	Chapter 10 (Diseases of the respiratory system)	0-4	10.6	10	0.3900
State of Mexico	Teotihuacán	Chapter 10 (Diseases of the respiratory system)	65+	17.0	10	0.0754
State of Mexico	Teotihuacán	Chapter 10 (Diseases of the respiratory system)	55-64	2.5	5	0.7706
State of Mexico	Teotihuacán	Chapter 5 (Mental and behavioural disorders)	65+	2.0	4	0.7409
State of Mexico	Teotihuacán	Chapter 5 (Mental and behavioural disorders)	55-64	2.8	4	0.5911
State of Mexico	Tepetlaoxtoc	Chapter 9 (Diseases of the circulatory system)	45-54	6.6	9	0.6787
State of Mexico	Tepetlaoxtoc	Chapter 9 (Diseases of the circulatory system)	55-64	12.5	10	0.2508
State of Mexico	Tepetlaoxtoc	Chapter 9 (Diseases of the circulatory system)	65+	15.8	10	0.1067
State of Mexico	Tepetlaoxtoc	Chapter 9 (Diseases of the circulatory system)	25-34	7.3	6	0.2900
State of Mexico	Tepetlaoxtoc	Chapter 9 (Diseases of the circulatory system)	35-44	2.2	5	0.8192
State of Mexico	Tepetlaoxtoc	Chapter 4 (Endocrine, nutritional and metabolic diseases)	25-34	3.6	5	0.6125
State of Mexico	Tepetlaoxtoc	Chapter 4 (Endocrine, nutritional and metabolic diseases)	35-44	8.1	6	0.2306
State of Mexico	Tepetlaoxtoc	Chapter 4 (Endocrine, nutritional and metabolic diseases)	55-64	13.0	10	0.2260
State of Mexico	Tepetlaoxtoc	Chapter 4 (Endocrine, nutritional and metabolic diseases)	65+	12.4	10	0.2587
State of Mexico	Tepetlaoxtoc	Chapter 4 (Endocrine, nutritional and metabolic diseases)	45-54	8.7	10	0.5592

State of Mexico	Tepetlaoxtoc	Chapter 6 (Diseases of the nervous system)	35-44	6.9	4	0.1402
State of Mexico	Tepetlaoxtoc	Chapter 6 (Diseases of the nervous system)	65+	4.1	4	0.3959
State of Mexico	Tepetlaoxtoc	Chapter 10 (Diseases of the respiratory system)	0-4	12.6	10	0.2496
State of Mexico	Tepetlaoxtoc	Chapter 10 (Diseases of the respiratory system)	65+	20.8	10	**0.0229***
State of Mexico	Tepetlaoxtoc	Chapter 10 (Diseases of the respiratory system)	55-64	6.5	6	0.3653
State of Mexico	Tepetlixpa	Chapter 9 (Diseases of the circulatory system)	65+	8.1	10	0.6151
State of Mexico	Tepetlixpa	Chapter 9 (Diseases of the circulatory system)	35-44	8.0	5	0.1566
State of Mexico	Tepetlixpa	Chapter 9 (Diseases of the circulatory system)	55-64	16.7	9	0.0541
State of Mexico	Tepetlixpa	Chapter 9 (Diseases of the circulatory system)	45-54	4.2	7	0.7544
State of Mexico	Tepetlixpa	Chapter 4 (Endocrine, nutritional and metabolic diseases)	35-44	13.2	10	0.2133
State of Mexico	Tepetlixpa	Chapter 4 (Endocrine, nutritional and metabolic diseases)	55-64	28.8	10	**0.0013***
State of Mexico	Tepetlixpa	Chapter 4 (Endocrine, nutritional and metabolic diseases)	65+	13.8	10	0.1802
State of Mexico	Tepetlixpa	Chapter 4 (Endocrine, nutritional and metabolic diseases)	45-54	10.1	10	0.4314
State of Mexico	Tepetlixpa	Chapter 6 (Diseases of the nervous system)	65+	1.9	10	0.9968
State of Mexico	Tepetlixpa	Chapter 10 (Diseases of the respiratory system)	0-4	23.3	10	**0.0096***
State of Mexico	Tepetlixpa	Chapter 10 (Diseases of the respiratory system)	65+	15.8	10	0.1070
State of Mexico	Tepetlixpa	Chapter 5 (Mental and behavioural disorders)	65+	9.9	8	0.2700
State of Mexico	Tepotzotlán	Chapter 9 (Diseases of the circulatory system)	35-44	12.2	9	0.1998
State of Mexico	Tepotzotlán	Chapter 9 (Diseases of the circulatory system)	45-54	12.0	10	0.2883
State of Mexico	Tepotzotlán	Chapter 9 (Diseases of the circulatory system)	55-64	18.6	10	**0.0453***
State of Mexico	Tepotzotlán	Chapter 9 (Diseases of the circulatory system)	65+	12.2	10	0.2706
State of Mexico	Tepotzotlán	Chapter 9 (Diseases of the circulatory system)	25-34	3.0	7	0.8882
State of Mexico	Tepotzotlán	Chapter 4 (Endocrine, nutritional and metabolic diseases)	25-34	8.0	7	0.3283
State of Mexico	Tepotzotlán	Chapter 4 (Endocrine, nutritional and metabolic diseases)	35-44	11.9	10	0.2931
State of Mexico	Tepotzotlán	Chapter 4 (Endocrine, nutritional and metabolic diseases)	45-54	9.8	10	0.4553
State of Mexico	Tepotzotlán	Chapter 4 (Endocrine, nutritional and metabolic diseases)	55-64	8.6	10	0.5741
State of Mexico	Tepotzotlán	Chapter 4 (Endocrine, nutritional and metabolic diseases)	65+	5.9	10	0.8272
State of Mexico	Tepotzotlán	Chapter 6 (Diseases of the nervous system)	15-24	1.8	7	0.9715
State of Mexico	Tepotzotlán	Chapter 6 (Diseases of the nervous system)	25-34	4.3	4	0.3665
State of Mexico	Tepotzotlán	Chapter 6 (Diseases of the nervous system)	5-14	1.6	4	0.8146
State of Mexico	Tepotzotlán	Chapter 6 (Diseases of the nervous system)	55-64	7.5	5	0.1884
State of Mexico	Tepotzotlán	Chapter 6 (Diseases of the nervous system)	65+	13.6	10	0.1942
State of Mexico	Tepotzotlán	Chapter 6 (Diseases of the nervous system)	45-54	3.2	4	0.5295
State of Mexico	Tepotzotlán	Chapter 10 (Diseases of the respiratory system)	0-4	12.1	10	0.2762
State of Mexico	Tepotzotlán	Chapter 10 (Diseases of the respiratory system)	55-64	10.4	10	0.4039
State of Mexico	Tepotzotlán	Chapter 10 (Diseases of the respiratory system)	65+	19.0	10	**0.0401***
State of Mexico	Tepotzotlán	Chapter 10 (Diseases of the respiratory system)	35-44	4.5	4	0.3454
State of Mexico	Tepotzotlán	Chapter 5 (Mental and behavioural disorders)	65+	11.4	9	0.2467
State of Mexico	Tequixquiac	Chapter 9 (Diseases of the circulatory system)	45-54	26.2	10	**0.0035***
State of Mexico	Tequixquiac	Chapter 9 (Diseases of the circulatory system)	55-64	5.6	10	0.8515
State of Mexico	Tequixquiac	Chapter 9 (Diseases of the circulatory system)	65+	11.6	10	0.3151
State of Mexico	Tequixquiac	Chapter 9 (Diseases of the circulatory system)	35-44	3.8	8	0.8741
State of Mexico	Tequixquiac	Chapter 4 (Endocrine, nutritional and metabolic diseases)	55-64	15.2	10	0.1237

State of Mexico	Tequixquiac	Chapter 4 (Endocrine, nutritional and metabolic diseases)	65+	11.0	10	0.3543
State of Mexico	Tequixquiac	Chapter 4 (Endocrine, nutritional and metabolic diseases)	45-54	7.8	10	0.6437
State of Mexico	Tequixquiac	Chapter 4 (Endocrine, nutritional and metabolic diseases)	35-44	11.7	9	0.2328
State of Mexico	Tequixquiac	Chapter 6 (Diseases of the nervous system)	65+	10.3	9	0.3278
State of Mexico	Tequixquiac	Chapter 10 (Diseases of the respiratory system)	65+	28.0	10	**0.0018***
State of Mexico	Tequixquiac	Chapter 10 (Diseases of the respiratory system)	0-4	3.8	10	0.9548
State of Mexico	Tequixquiac	Chapter 10 (Diseases of the respiratory system)	55-64	3.0	4	0.5578
State of Mexico	Tequixquiac	Chapter 5 (Mental and behavioural disorders)	65+	7.8	6	0.2547
State of Mexico	Texcoco	Chapter 9 (Diseases of the circulatory system)	25-34	16.4	10	0.0898
State of Mexico	Texcoco	Chapter 9 (Diseases of the circulatory system)	35-44	7.5	10	0.6727
State of Mexico	Texcoco	Chapter 9 (Diseases of the circulatory system)	45-54	22.5	10	**0.0129***
State of Mexico	Texcoco	Chapter 9 (Diseases of the circulatory system)	55-64	12.6	10	0.2458
State of Mexico	Texcoco	Chapter 9 (Diseases of the circulatory system)	65+	11.5	10	0.3209
State of Mexico	Texcoco	Chapter 9 (Diseases of the circulatory system)	0-4	5.5	10	0.8582
State of Mexico	Texcoco	Chapter 9 (Diseases of the circulatory system)	15-24	7.4	10	0.6918
State of Mexico	Texcoco	Chapter 4 (Endocrine, nutritional and metabolic diseases)	0-4	9.2	10	0.5163
State of Mexico	Texcoco	Chapter 4 (Endocrine, nutritional and metabolic diseases)	25-34	10.5	10	0.3978
State of Mexico	Texcoco	Chapter 4 (Endocrine, nutritional and metabolic diseases)	35-44	8.1	10	0.6214
State of Mexico	Texcoco	Chapter 4 (Endocrine, nutritional and metabolic diseases)	45-54	9.1	10	0.5227
State of Mexico	Texcoco	Chapter 4 (Endocrine, nutritional and metabolic diseases)	55-64	9.2	10	0.5147
State of Mexico	Texcoco	Chapter 4 (Endocrine, nutritional and metabolic diseases)	65+	6.3	10	0.7906
State of Mexico	Texcoco	Chapter 4 (Endocrine, nutritional and metabolic diseases)	15-24	3.4	10	0.9695
State of Mexico	Texcoco	Chapter 4 (Endocrine, nutritional and metabolic diseases)	5-14	6.6	6	0.3580
State of Mexico	Texcoco	Chapter 6 (Diseases of the nervous system)	15-24	5.9	10	0.8262
State of Mexico	Texcoco	Chapter 6 (Diseases of the nervous system)	65+	6.0	10	0.8117
State of Mexico	Texcoco	Chapter 6 (Diseases of the nervous system)	25-34	15.2	10	0.1266
State of Mexico	Texcoco	Chapter 6 (Diseases of the nervous system)	35-44	5.1	10	0.8817
State of Mexico	Texcoco	Chapter 6 (Diseases of the nervous system)	45-54	9.3	10	0.5048
State of Mexico	Texcoco	Chapter 6 (Diseases of the nervous system)	55-64	17.7	10	0.0600
State of Mexico	Texcoco	Chapter 6 (Diseases of the nervous system)	0-4	10.4	10	0.4028
State of Mexico	Texcoco	Chapter 6 (Diseases of the nervous system)	5-14	6.2	6	0.4019
State of Mexico	Texcoco	Chapter 10 (Diseases of the respiratory system)	0-4	15.3	10	0.1213
State of Mexico	Texcoco	Chapter 10 (Diseases of the respiratory system)	15-24	1.6	7	0.9786
State of Mexico	Texcoco	Chapter 10 (Diseases of the respiratory system)	35-44	10.6	10	0.3870
State of Mexico	Texcoco	Chapter 10 (Diseases of the respiratory system)	45-54	7.6	10	0.6708
State of Mexico	Texcoco	Chapter 10 (Diseases of the respiratory system)	55-64	5.6	10	0.8477
State of Mexico	Texcoco	Chapter 10 (Diseases of the respiratory system)	65+	10.8	10	0.3703
State of Mexico	Texcoco	Chapter 10 (Diseases of the respiratory system)	25-34	7.6	10	0.6652
State of Mexico	Texcoco	Chapter 10 (Diseases of the respiratory system)	5-14	9.3	8	0.3146
State of Mexico	Texcoco	Chapter 5 (Mental and behavioural disorders)	35-44	10.4	10	0.4042
State of Mexico	Texcoco	Chapter 5 (Mental and behavioural disorders)	25-34	5.6	5	0.3450
State of Mexico	Texcoco	Chapter 5 (Mental and behavioural disorders)	45-54	7.2	10	0.7049
State of Mexico	Texcoco	Chapter 5 (Mental and behavioural disorders)	55-64	8.3	10	0.5966

State of Mexico	Texcoco	Chapter 5 (Mental and behavioural disorders)	65+	6.9	10	0.7384
State of Mexico	Tezoyuca	Chapter 9 (Diseases of the circulatory system)	65+	13.8	10	0.1829
State of Mexico	Tezoyuca	Chapter 9 (Diseases of the circulatory system)	35-44	9.0	6	0.1725
State of Mexico	Tezoyuca	Chapter 9 (Diseases of the circulatory system)	45-54	8.2	10	0.6115
State of Mexico	Tezoyuca	Chapter 9 (Diseases of the circulatory system)	55-64	11.1	10	0.3470
State of Mexico	Tezoyuca	Chapter 4 (Endocrine, nutritional and metabolic diseases)	55-64	13.1	10	0.2192
State of Mexico	Tezoyuca	Chapter 4 (Endocrine, nutritional and metabolic diseases)	65+	7.5	10	0.6757
State of Mexico	Tezoyuca	Chapter 4 (Endocrine, nutritional and metabolic diseases)	45-54	7.3	10	0.6944
State of Mexico	Tezoyuca	Chapter 6 (Diseases of the nervous system)	65+	8.4	7	0.2996
State of Mexico	Tezoyuca	Chapter 10 (Diseases of the respiratory system)	0-4	13.2	10	0.2145
State of Mexico	Tezoyuca	Chapter 10 (Diseases of the respiratory system)	65+	7.6	10	0.6648
State of Mexico	Tezoyuca	Chapter 10 (Diseases of the respiratory system)	55-64	5.3	4	0.2555
State of Mexico	Tlalmanalco	Chapter 9 (Diseases of the circulatory system)	55-64	19.6	10	**0.0330***
State of Mexico	Tlalmanalco	Chapter 9 (Diseases of the circulatory system)	65+	11.0	10	0.3589
State of Mexico	Tlalmanalco	Chapter 9 (Diseases of the circulatory system)	35-44	19.3	10	**0.0368***
State of Mexico	Tlalmanalco	Chapter 9 (Diseases of the circulatory system)	45-54	7.5	10	0.6730
State of Mexico	Tlalmanalco	Chapter 4 (Endocrine, nutritional and metabolic diseases)	35-44	9.4	10	0.4925
State of Mexico	Tlalmanalco	Chapter 4 (Endocrine, nutritional and metabolic diseases)	45-54	6.9	10	0.7331
State of Mexico	Tlalmanalco	Chapter 4 (Endocrine, nutritional and metabolic diseases)	55-64	6.2	10	0.7999
State of Mexico	Tlalmanalco	Chapter 4 (Endocrine, nutritional and metabolic diseases)	65+	14.2	10	0.1642
State of Mexico	Tlalmanalco	Chapter 4 (Endocrine, nutritional and metabolic diseases)	25-34	8.1	5	0.1520
State of Mexico	Tlalmanalco	Chapter 6 (Diseases of the nervous system)	65+	20.6	10	**0.0244***
State of Mexico	Tlalmanalco	Chapter 6 (Diseases of the nervous system)	35-44	11.8	4	**0.0189***
State of Mexico	Tlalmanalco	Chapter 6 (Diseases of the nervous system)	15-24	7.8	5	0.1702
State of Mexico	Tlalmanalco	Chapter 10 (Diseases of the respiratory system)	0-4	10.2	10	0.4271
State of Mexico	Tlalmanalco	Chapter 10 (Diseases of the respiratory system)	55-64	9.1	10	0.5210
State of Mexico	Tlalmanalco	Chapter 10 (Diseases of the respiratory system)	65+	9.9	10	0.4454
State of Mexico	Tlalmanalco	Chapter 10 (Diseases of the respiratory system)	15-24	2.3	4	0.6757
State of Mexico	Tlalmanalco	Chapter 10 (Diseases of the respiratory system)	35-44	5.5	5	0.3599
State of Mexico	Tlalmanalco	Chapter 5 (Mental and behavioural disorders)	55-64	8.7	5	0.1201
State of Mexico	Tlalmanalco	Chapter 5 (Mental and behavioural disorders)	25-34	5.2	4	0.2695
State of Mexico	Tlalmanalco	Chapter 5 (Mental and behavioural disorders)	35-44	6.8	5	0.2391
State of Mexico	Tlalmanalco	Chapter 5 (Mental and behavioural disorders)	65+	6.5	5	0.2629
State of Mexico	Tlalnepantla de Baz	Chapter 9 (Diseases of the circulatory system)	0-4	5.6	10	0.8511
State of Mexico	Tlalnepantla de Baz	Chapter 9 (Diseases of the circulatory system)	25-34	10.4	10	0.4054
State of Mexico	Tlalnepantla de Baz	Chapter 9 (Diseases of the circulatory system)	35-44	33.1	10	**0.0003***
State of Mexico	Tlalnepantla de Baz	Chapter 9 (Diseases of the circulatory system)	45-54	9.4	10	0.4951
State of Mexico	Tlalnepantla de Baz	Chapter 9 (Diseases of the circulatory system)	55-64	6.8	10	0.7446
State of Mexico	Tlalnepantla de Baz	Chapter 9 (Diseases of the circulatory system)	65+	15.7	10	0.1095
State of Mexico	Tlalnepantla de Baz	Chapter 9 (Diseases of the circulatory system)	15-24	12.0	10	0.2851
State of Mexico	Tlalnepantla de Baz	Chapter 9 (Diseases of the circulatory system)	5-14	7.5	6	0.2762
State of Mexico	Tlalnepantla de Baz	Chapter 4 (Endocrine, nutritional and metabolic diseases)	0-4	12.9	10	0.2265
State of Mexico	Tlalnepantla de Baz	Chapter 4 (Endocrine, nutritional and metabolic diseases)	5-14	2.9	7	0.8918

State of Mexico	Tlalnepantla de Baz	Chapter 4 (Endocrine, nutritional and metabolic diseases)	15-24	11.3	10	0.3371
State of Mexico	Tlalnepantla de Baz	Chapter 4 (Endocrine, nutritional and metabolic diseases)	25-34	22.8	10	**0.0114***
State of Mexico	Tlalnepantla de Baz	Chapter 4 (Endocrine, nutritional and metabolic diseases)	35-44	11.4	10	0.3286
State of Mexico	Tlalnepantla de Baz	Chapter 4 (Endocrine, nutritional and metabolic diseases)	45-54	13.2	10	0.2141
State of Mexico	Tlalnepantla de Baz	Chapter 4 (Endocrine, nutritional and metabolic diseases)	55-64	8.0	10	0.6271
State of Mexico	Tlalnepantla de Baz	Chapter 4 (Endocrine, nutritional and metabolic diseases)	65+	6.8	10	0.7481
State of Mexico	Tlalnepantla de Baz	Chapter 6 (Diseases of the nervous system)	0-4	11.3	10	0.3312
State of Mexico	Tlalnepantla de Baz	Chapter 6 (Diseases of the nervous system)	5-14	13.2	10	0.2123
State of Mexico	Tlalnepantla de Baz	Chapter 6 (Diseases of the nervous system)	25-34	8.3	10	0.5977
State of Mexico	Tlalnepantla de Baz	Chapter 6 (Diseases of the nervous system)	35-44	4.6	10	0.9171
State of Mexico	Tlalnepantla de Baz	Chapter 6 (Diseases of the nervous system)	45-54	7.5	10	0.6783
State of Mexico	Tlalnepantla de Baz	Chapter 6 (Diseases of the nervous system)	55-64	8.7	10	0.5606
State of Mexico	Tlalnepantla de Baz	Chapter 6 (Diseases of the nervous system)	65+	8.5	10	0.5760
State of Mexico	Tlalnepantla de Baz	Chapter 6 (Diseases of the nervous system)	15-24	3.6	10	0.9631
State of Mexico	Tlalnepantla de Baz	Chapter 10 (Diseases of the respiratory system)	0-4	16.1	10	0.0970
State of Mexico	Tlalnepantla de Baz	Chapter 10 (Diseases of the respiratory system)	15-24	3.6	10	0.9642
State of Mexico	Tlalnepantla de Baz	Chapter 10 (Diseases of the respiratory system)	25-34	17.4	10	0.0663
State of Mexico	Tlalnepantla de Baz	Chapter 10 (Diseases of the respiratory system)	35-44	11.1	10	0.3486
State of Mexico	Tlalnepantla de Baz	Chapter 10 (Diseases of the respiratory system)	45-54	11.5	10	0.3179
State of Mexico	Tlalnepantla de Baz	Chapter 10 (Diseases of the respiratory system)	55-64	26.0	10	**0.0037***
State of Mexico	Tlalnepantla de Baz	Chapter 10 (Diseases of the respiratory system)	65+	25.6	10	**0.0044***
State of Mexico	Tlalnepantla de Baz	Chapter 10 (Diseases of the respiratory system)	5-14	5.0	8	0.7628
State of Mexico	Tlalnepantla de Baz	Chapter 5 (Mental and behavioural disorders)	15-24	3.0	4	0.5602
State of Mexico	Tlalnepantla de Baz	Chapter 5 (Mental and behavioural disorders)	25-34	6.5	8	0.5906
State of Mexico	Tlalnepantla de Baz	Chapter 5 (Mental and behavioural disorders)	35-44	9.9	10	0.4495
State of Mexico	Tlalnepantla de Baz	Chapter 5 (Mental and behavioural disorders)	45-54	12.2	10	0.2730
State of Mexico	Tlalnepantla de Baz	Chapter 5 (Mental and behavioural disorders)	65+	6.4	10	0.7795
State of Mexico	Tlalnepantla de Baz	Chapter 5 (Mental and behavioural disorders)	55-64	6.2	10	0.7957
State of Mexico	Tultepec	Chapter 9 (Diseases of the circulatory system)	35-44	7.6	10	0.6660
State of Mexico	Tultepec	Chapter 9 (Diseases of the circulatory system)	55-64	6.4	10	0.7833
State of Mexico	Tultepec	Chapter 9 (Diseases of the circulatory system)	65+	9.4	10	0.4930
State of Mexico	Tultepec	Chapter 9 (Diseases of the circulatory system)	45-54	22.6	10	**0.0125**
State of Mexico	Tultepec	Chapter 9 (Diseases of the circulatory system)	15-24	2.7	6	0.8458
State of Mexico	Tultepec	Chapter 9 (Diseases of the circulatory system)	25-34	9.5	8	0.3055
State of Mexico	Tultepec	Chapter 4 (Endocrine, nutritional and metabolic diseases)	35-44	9.3	10	0.5063
State of Mexico	Tultepec	Chapter 4 (Endocrine, nutritional and metabolic diseases)	45-54	7.2	10	0.7040
State of Mexico	Tultepec	Chapter 4 (Endocrine, nutritional and metabolic diseases)	55-64	7.3	10	0.6921
State of Mexico	Tultepec	Chapter 4 (Endocrine, nutritional and metabolic diseases)	65+	11.3	10	0.3354
State of Mexico	Tultepec	Chapter 4 (Endocrine, nutritional and metabolic diseases)	25-34	9.4	7	0.2221
State of Mexico	Tultepec	Chapter 4 (Endocrine, nutritional and metabolic diseases)	0-4	2.8	4	0.5958
State of Mexico	Tultepec	Chapter 6 (Diseases of the nervous system)	35-44	2.6	5	0.7660
State of Mexico	Tultepec	Chapter 6 (Diseases of the nervous system)	65+	8.9	10	0.5377
State of Mexico	Tultepec	Chapter 6 (Diseases of the nervous system)	5-14	2.1	4	0.7234

State of Mexico	Tultepec	Chapter 6 (Diseases of the nervous system)	15-24	2.6	6	0.8619
State of Mexico	Tultepec	Chapter 6 (Diseases of the nervous system)	25-34	1.8	8	0.9862
State of Mexico	Tultepec	Chapter 6 (Diseases of the nervous system)	55-64	4.3	4	0.3668
State of Mexico	Tultepec	Chapter 10 (Diseases of the respiratory system)	0-4	25.2	10	**0.0049***
State of Mexico	Tultepec	Chapter 10 (Diseases of the respiratory system)	45-54	12.9	10	0.2304
State of Mexico	Tultepec	Chapter 10 (Diseases of the respiratory system)	55-64	12.5	10	0.2507
State of Mexico	Tultepec	Chapter 10 (Diseases of the respiratory system)	65+	11.4	10	0.3279
State of Mexico	Tultepec	Chapter 10 (Diseases of the respiratory system)	35-44	5.6	4	0.2297
State of Mexico	Tultepec	Chapter 5 (Mental and behavioural disorders)	55-64	13.6	6	**0.0341***
State of Mexico	Tultepec	Chapter 5 (Mental and behavioural disorders)	65+	7.1	10	0.7197
State of Mexico	Tultitlán	Chapter 9 (Diseases of the circulatory system)	15-24	6.4	9	0.6974
State of Mexico	Tultitlán	Chapter 9 (Diseases of the circulatory system)	25-34	13.3	10	0.2059
State of Mexico	Tultitlán	Chapter 9 (Diseases of the circulatory system)	35-44	5.9	10	0.8238
State of Mexico	Tultitlán	Chapter 9 (Diseases of the circulatory system)	45-54	14.6	10	0.1464
State of Mexico	Tultitlán	Chapter 9 (Diseases of the circulatory system)	55-64	10.9	10	0.3663
State of Mexico	Tultitlán	Chapter 9 (Diseases of the circulatory system)	65+	9.2	10	0.5158
State of Mexico	Tultitlán	Chapter 9 (Diseases of the circulatory system)	0-4	5.6	4	0.2274
State of Mexico	Tultitlán	Chapter 4 (Endocrine, nutritional and metabolic diseases)	25-34	8.2	10	0.6135
State of Mexico	Tultitlán	Chapter 4 (Endocrine, nutritional and metabolic diseases)	35-44	5.8	10	0.8293
State of Mexico	Tultitlán	Chapter 4 (Endocrine, nutritional and metabolic diseases)	45-54	8.9	10	0.5382
State of Mexico	Tultitlán	Chapter 4 (Endocrine, nutritional and metabolic diseases)	55-64	9.0	10	0.5321
State of Mexico	Tultitlán	Chapter 4 (Endocrine, nutritional and metabolic diseases)	65+	14.5	10	0.1500
State of Mexico	Tultitlán	Chapter 4 (Endocrine, nutritional and metabolic diseases)	0-4	18.8	10	**0.0435***
State of Mexico	Tultitlán	Chapter 4 (Endocrine, nutritional and metabolic diseases)	15-24	11.3	10	0.3375
State of Mexico	Tultitlán	Chapter 6 (Diseases of the nervous system)	0-4	3.4	10	0.9714
State of Mexico	Tultitlán	Chapter 6 (Diseases of the nervous system)	5-14	18.5	10	**0.0470***
State of Mexico	Tultitlán	Chapter 6 (Diseases of the nervous system)	15-24	8.8	10	0.5493
State of Mexico	Tultitlán	Chapter 6 (Diseases of the nervous system)	25-34	7.5	10	0.6778
State of Mexico	Tultitlán	Chapter 6 (Diseases of the nervous system)	35-44	9.4	10	0.4903
State of Mexico	Tultitlán	Chapter 6 (Diseases of the nervous system)	65+	12.8	10	0.2332
State of Mexico	Tultitlán	Chapter 6 (Diseases of the nervous system)	45-54	15.9	9	0.0690
State of Mexico	Tultitlán	Chapter 6 (Diseases of the nervous system)	55-64	8.5	10	0.5848
State of Mexico	Tultitlán	Chapter 10 (Diseases of the respiratory system)	0-4	9.8	10	0.4569
State of Mexico	Tultitlán	Chapter 10 (Diseases of the respiratory system)	15-24	10.8	10	0.3707
State of Mexico	Tultitlán	Chapter 10 (Diseases of the respiratory system)	25-34	18.6	10	**0.0462***
State of Mexico	Tultitlán	Chapter 10 (Diseases of the respiratory system)	35-44	11.0	10	0.3599
State of Mexico	Tultitlán	Chapter 10 (Diseases of the respiratory system)	55-64	14.4	10	0.1566
State of Mexico	Tultitlán	Chapter 10 (Diseases of the respiratory system)	65+	12.5	10	0.2513
State of Mexico	Tultitlán	Chapter 10 (Diseases of the respiratory system)	45-54	17.1	10	0.0726
State of Mexico	Tultitlán	Chapter 10 (Diseases of the respiratory system)	5-14	12.5	8	0.1321
State of Mexico	Tultitlán	Chapter 5 (Mental and behavioural disorders)	35-44	10.6	9	0.3068
State of Mexico	Tultitlán	Chapter 5 (Mental and behavioural disorders)	55-64	13.7	8	0.0896
State of Mexico	Tultitlán	Chapter 5 (Mental and behavioural disorders)	65+	16.0	10	0.1006

State of Mexico	Tultitlán	Chapter 5 (Mental and behavioural disorders)	45-54	15.4	10	0.1195
State of Mexico	Tultitlán	Chapter 5 (Mental and behavioural disorders)	25-34	4.7	7	0.6967
State of Mexico	Villa del Carbón	Chapter 9 (Diseases of the circulatory system)	45-54	14.2	10	0.1644
State of Mexico	Villa del Carbón	Chapter 9 (Diseases of the circulatory system)	55-64	16.3	10	0.0924
State of Mexico	Villa del Carbón	Chapter 9 (Diseases of the circulatory system)	65+	14.2	10	0.1630
State of Mexico	Villa del Carbón	Chapter 9 (Diseases of the circulatory system)	25-34	11.3	8	0.1846
State of Mexico	Villa del Carbón	Chapter 9 (Diseases of the circulatory system)	35-44	19.0	10	**0.0401***
State of Mexico	Villa del Carbón	Chapter 4 (Endocrine, nutritional and metabolic diseases)	0-4	2.8	10	0.9859
State of Mexico	Villa del Carbón	Chapter 4 (Endocrine, nutritional and metabolic diseases)	35-44	1.9	8	0.9829
State of Mexico	Villa del Carbón	Chapter 4 (Endocrine, nutritional and metabolic diseases)	65+	10.2	10	0.4273
State of Mexico	Villa del Carbón	Chapter 4 (Endocrine, nutritional and metabolic diseases)	55-64	16.7	10	0.0811
State of Mexico	Villa del Carbón	Chapter 4 (Endocrine, nutritional and metabolic diseases)	45-54	3.5	10	0.9681
State of Mexico	Villa del Carbón	Chapter 6 (Diseases of the nervous system)	5-14	3.5	6	0.7464
State of Mexico	Villa del Carbón	Chapter 6 (Diseases of the nervous system)	15-24	3.4	4	0.4976
State of Mexico	Villa del Carbón	Chapter 6 (Diseases of the nervous system)	65+	2.7	6	0.8478
State of Mexico	Villa del Carbón	Chapter 10 (Diseases of the respiratory system)	0-4	7.2	10	0.7062
State of Mexico	Villa del Carbón	Chapter 10 (Diseases of the respiratory system)	45-54	1.9	9	0.9925
State of Mexico	Villa del Carbón	Chapter 10 (Diseases of the respiratory system)	55-64	7.5	10	0.6813
State of Mexico	Villa del Carbón	Chapter 10 (Diseases of the respiratory system)	65+	3.1	10	0.9801
State of Mexico	Villa del Carbón	Chapter 5 (Mental and behavioural disorders)	55-64	3.8	8	0.8775
State of Mexico	Villa del Carbón	Chapter 5 (Mental and behavioural disorders)	65+	9.3	4	0.0538
State of Mexico	Zumpango	Chapter 9 (Diseases of the circulatory system)	25-34	9.7	10	0.4639
State of Mexico	Zumpango	Chapter 9 (Diseases of the circulatory system)	35-44	11.6	10	0.3142
State of Mexico	Zumpango	Chapter 9 (Diseases of the circulatory system)	45-54	12.1	10	0.2805
State of Mexico	Zumpango	Chapter 9 (Diseases of the circulatory system)	55-64	12.1	10	0.2753
State of Mexico	Zumpango	Chapter 9 (Diseases of the circulatory system)	65+	6.0	10	0.8178
State of Mexico	Zumpango	Chapter 9 (Diseases of the circulatory system)	0-4	7.2	5	0.2084
State of Mexico	Zumpango	Chapter 9 (Diseases of the circulatory system)	15-24	6.4	7	0.4964
State of Mexico	Zumpango	Chapter 4 (Endocrine, nutritional and metabolic diseases)	0-4	14.8	10	0.1400
State of Mexico	Zumpango	Chapter 4 (Endocrine, nutritional and metabolic diseases)	35-44	9.0	10	0.5313
State of Mexico	Zumpango	Chapter 4 (Endocrine, nutritional and metabolic diseases)	45-54	6.2	10	0.7960
State of Mexico	Zumpango	Chapter 4 (Endocrine, nutritional and metabolic diseases)	55-64	16.4	10	0.0891
State of Mexico	Zumpango	Chapter 4 (Endocrine, nutritional and metabolic diseases)	65+	14.7	10	0.1429
State of Mexico	Zumpango	Chapter 4 (Endocrine, nutritional and metabolic diseases)	15-24	2.7	5	0.7430
State of Mexico	Zumpango	Chapter 4 (Endocrine, nutritional and metabolic diseases)	25-34	12.8	9	0.1721
State of Mexico	Zumpango	Chapter 6 (Diseases of the nervous system)	0-4	5.0	6	0.5466
State of Mexico	Zumpango	Chapter 6 (Diseases of the nervous system)	25-34	15.3	10	0.1225
State of Mexico	Zumpango	Chapter 6 (Diseases of the nervous system)	65+	6.4	10	0.7801
State of Mexico	Zumpango	Chapter 6 (Diseases of the nervous system)	15-24	6.3	7	0.5048
State of Mexico	Zumpango	Chapter 6 (Diseases of the nervous system)	35-44	4.1	10	0.9435
State of Mexico	Zumpango	Chapter 6 (Diseases of the nervous system)	45-54	22.4	9	**0.0077***
State of Mexico	Zumpango	Chapter 6 (Diseases of the nervous system)	55-64	13.2	7	0.0675
State of Mexico	Zumpango	Chapter 6 (Diseases of the nervous system)	5-14	2.8	8	0.9467

State of Mexico	Zumpango	Chapter 10 (Diseases of the respiratory system)	0-4	12.8	10	0.2322
State of Mexico	Zumpango	Chapter 10 (Diseases of the respiratory system)	5-14	2.7	4	0.6025
State of Mexico	Zumpango	Chapter 10 (Diseases of the respiratory system)	45-54	11.4	10	0.3276
State of Mexico	Zumpango	Chapter 10 (Diseases of the respiratory system)	65+	14.1	10	0.1698
State of Mexico	Zumpango	Chapter 10 (Diseases of the respiratory system)	55-64	16.1	10	0.0964
State of Mexico	Zumpango	Chapter 10 (Diseases of the respiratory system)	35-44	5.7	10	0.8416
State of Mexico	Zumpango	Chapter 10 (Diseases of the respiratory system)	25-34	4.5	5	0.4800
State of Mexico	Zumpango	Chapter 5 (Mental and behavioural disorders)	35-44	8.6	8	0.3755
State of Mexico	Zumpango	Chapter 5 (Mental and behavioural disorders)	45-54	6.5	4	0.1641
State of Mexico	Zumpango	Chapter 5 (Mental and behavioural disorders)	65+	31.4	10	**0.0005***
State of Mexico	Cuautitlán Izcalli	Chapter 9 (Diseases of the circulatory system)	15-24	6.5	10	0.7700
State of Mexico	Cuautitlán Izcalli	Chapter 9 (Diseases of the circulatory system)	25-34	7.0	10	0.7210
State of Mexico	Cuautitlán Izcalli	Chapter 9 (Diseases of the circulatory system)	35-44	10.6	10	0.3928
State of Mexico	Cuautitlán Izcalli	Chapter 9 (Diseases of the circulatory system)	45-54	15.7	10	0.1076
State of Mexico	Cuautitlán Izcalli	Chapter 9 (Diseases of the circulatory system)	55-64	9.4	10	0.4972
State of Mexico	Cuautitlán Izcalli	Chapter 9 (Diseases of the circulatory system)	65+	14.1	10	0.1702
State of Mexico	Cuautitlán Izcalli	Chapter 9 (Diseases of the circulatory system)	0-4	15.5	7	**0.0299***
State of Mexico	Cuautitlán Izcalli	Chapter 9 (Diseases of the circulatory system)	5-14	3.4	5	0.6384
State of Mexico	Cuautitlán Izcalli	Chapter 4 (Endocrine, nutritional and metabolic diseases)	25-34	11.6	10	0.3107
State of Mexico	Cuautitlán Izcalli	Chapter 4 (Endocrine, nutritional and metabolic diseases)	35-44	16.4	10	0.0888
State of Mexico	Cuautitlán Izcalli	Chapter 4 (Endocrine, nutritional and metabolic diseases)	45-54	13.6	10	0.1914
State of Mexico	Cuautitlán Izcalli	Chapter 4 (Endocrine, nutritional and metabolic diseases)	55-64	9.7	10	0.4692
State of Mexico	Cuautitlán Izcalli	Chapter 4 (Endocrine, nutritional and metabolic diseases)	65+	7.5	10	0.6805
State of Mexico	Cuautitlán Izcalli	Chapter 4 (Endocrine, nutritional and metabolic diseases)	0-4	16.0	10	0.0987
State of Mexico	Cuautitlán Izcalli	Chapter 4 (Endocrine, nutritional and metabolic diseases)	15-24	7.2	10	0.7038
State of Mexico	Cuautitlán Izcalli	Chapter 6 (Diseases of the nervous system)	0-4	14.3	10	0.1608
State of Mexico	Cuautitlán Izcalli	Chapter 6 (Diseases of the nervous system)	5-14	14.1	10	0.1670
State of Mexico	Cuautitlán Izcalli	Chapter 6 (Diseases of the nervous system)	15-24	7.6	10	0.6673
State of Mexico	Cuautitlán Izcalli	Chapter 6 (Diseases of the nervous system)	25-34	7.4	10	0.6870
State of Mexico	Cuautitlán Izcalli	Chapter 6 (Diseases of the nervous system)	35-44	6.7	10	0.7533
State of Mexico	Cuautitlán Izcalli	Chapter 6 (Diseases of the nervous system)	45-54	8.2	10	0.6053
State of Mexico	Cuautitlán Izcalli	Chapter 6 (Diseases of the nervous system)	55-64	14.1	10	0.1685
State of Mexico	Cuautitlán Izcalli	Chapter 6 (Diseases of the nervous system)	65+	6.8	10	0.7449
State of Mexico	Cuautitlán Izcalli	Chapter 10 (Diseases of the respiratory system)	0-4	8.0	10	0.6295
State of Mexico	Cuautitlán Izcalli	Chapter 10 (Diseases of the respiratory system)	5-14	3.6	5	0.6101
State of Mexico	Cuautitlán Izcalli	Chapter 10 (Diseases of the respiratory system)	15-24	10.6	10	0.3864
State of Mexico	Cuautitlán Izcalli	Chapter 10 (Diseases of the respiratory system)	35-44	10.5	10	0.3972
State of Mexico	Cuautitlán Izcalli	Chapter 10 (Diseases of the respiratory system)	45-54	12.2	10	0.2718
State of Mexico	Cuautitlán Izcalli	Chapter 10 (Diseases of the respiratory system)	55-64	9.4	10	0.4969
State of Mexico	Cuautitlán Izcalli	Chapter 10 (Diseases of the respiratory system)	65+	18.8	10	**0.0432***
State of Mexico	Cuautitlán Izcalli	Chapter 10 (Diseases of the respiratory system)	25-34	10.9	10	0.3638
State of Mexico	Cuautitlán Izcalli	Chapter 5 (Mental and behavioural disorders)	35-44	4.1	7	0.7672
State of Mexico	Cuautitlán Izcalli	Chapter 5 (Mental and behavioural disorders)	45-54	13.1	10	0.2162

State of Mexico	Cuautitlán Izcalli	Chapter 5 (Mental and behavioural disorders)	55-64	16.4	9	0.0591
State of Mexico	Cuautitlán Izcalli	Chapter 5 (Mental and behavioural disorders)	65+	10.1	10	0.4337
State of Mexico	Cuautitlán Izcalli	Chapter 5 (Mental and behavioural disorders)	25-34	2.2	4	0.6958
State of Mexico	Valle de Chalco Solidaridad	Chapter 9 (Diseases of the circulatory system)	5-14	12.2	4	**0.0161***
State of Mexico	Valle de Chalco Solidaridad	Chapter 9 (Diseases of the circulatory system)	15-24	10.4	10	0.4061
State of Mexico	Valle de Chalco Solidaridad	Chapter 9 (Diseases of the circulatory system)	25-34	22.9	10	**0.0112***
State of Mexico	Valle de Chalco Solidaridad	Chapter 9 (Diseases of the circulatory system)	35-44	16.3	10	0.0925
State of Mexico	Valle de Chalco Solidaridad	Chapter 9 (Diseases of the circulatory system)	45-54	13.8	10	0.1810
State of Mexico	Valle de Chalco Solidaridad	Chapter 9 (Diseases of the circulatory system)	55-64	7.7	10	0.6570
State of Mexico	Valle de Chalco Solidaridad	Chapter 9 (Diseases of the circulatory system)	65+	7.2	10	0.7045
State of Mexico	Valle de Chalco Solidaridad	Chapter 9 (Diseases of the circulatory system)	0-4	4.1	6	0.6614
State of Mexico	Valle de Chalco Solidaridad	Chapter 4 (Endocrine, nutritional and metabolic diseases)	0-4	11.2	10	0.3412
State of Mexico	Valle de Chalco Solidaridad	Chapter 4 (Endocrine, nutritional and metabolic diseases)	15-24	14.0	10	0.1718
State of Mexico	Valle de Chalco Solidaridad	Chapter 4 (Endocrine, nutritional and metabolic diseases)	25-34	5.3	10	0.8716
State of Mexico	Valle de Chalco Solidaridad	Chapter 4 (Endocrine, nutritional and metabolic diseases)	35-44	22.7	10	**0.0120***
State of Mexico	Valle de Chalco Solidaridad	Chapter 4 (Endocrine, nutritional and metabolic diseases)	45-54	7.9	10	0.6368
State of Mexico	Valle de Chalco Solidaridad	Chapter 4 (Endocrine, nutritional and metabolic diseases)	55-64	11.9	10	0.2919
State of Mexico	Valle de Chalco Solidaridad	Chapter 4 (Endocrine, nutritional and metabolic diseases)	65+	21.4	10	**0.0183***
State of Mexico	Valle de Chalco Solidaridad	Chapter 4 (Endocrine, nutritional and metabolic diseases)	5-14	7.8	5	0.1683
State of Mexico	Valle de Chalco Solidaridad	Chapter 6 (Diseases of the nervous system)	15-24	8.6	10	0.5717
State of Mexico	Valle de Chalco Solidaridad	Chapter 6 (Diseases of the nervous system)	25-34	13.1	10	0.2204
State of Mexico	Valle de Chalco Solidaridad	Chapter 6 (Diseases of the nervous system)	35-44	15.9	10	0.1034
State of Mexico	Valle de Chalco Solidaridad	Chapter 6 (Diseases of the nervous system)	45-54	8.0	10	0.6292
State of Mexico	Valle de Chalco Solidaridad	Chapter 6 (Diseases of the nervous system)	0-4	7.6	10	0.6644
State of Mexico	Valle de Chalco Solidaridad	Chapter 6 (Diseases of the nervous system)	5-14	8.7	10	0.5572
State of Mexico	Valle de Chalco Solidaridad	Chapter 6 (Diseases of the nervous system)	65+	8.9	10	0.5416
State of Mexico	Valle de Chalco Solidaridad	Chapter 6 (Diseases of the nervous system)	55-64	8.2	10	0.6051
State of Mexico	Valle de Chalco Solidaridad	Chapter 10 (Diseases of the respiratory system)	0-4	22.4	10	**0.0131***
State of Mexico	Valle de Chalco Solidaridad	Chapter 10 (Diseases of the respiratory system)	5-14	9.0	10	0.5338
State of Mexico	Valle de Chalco Solidaridad	Chapter 10 (Diseases of the respiratory system)	25-34	7.2	10	0.7088
State of Mexico	Valle de Chalco Solidaridad	Chapter 10 (Diseases of the respiratory system)	35-44	27.9	10	**0.0018***
State of Mexico	Valle de Chalco Solidaridad	Chapter 10 (Diseases of the respiratory system)	45-54	9.3	10	0.5048
State of Mexico	Valle de Chalco Solidaridad	Chapter 10 (Diseases of the respiratory system)	55-64	16.5	10	0.0858
State of Mexico	Valle de Chalco Solidaridad	Chapter 10 (Diseases of the respiratory system)	65+	13.4	10	0.2029
State of Mexico	Valle de Chalco Solidaridad	Chapter 10 (Diseases of the respiratory system)	15-24	11.3	10	0.3331
State of Mexico	Valle de Chalco Solidaridad	Chapter 5 (Mental and behavioural disorders)	15-24	2.7	4	0.6034
State of Mexico	Valle de Chalco Solidaridad	Chapter 5 (Mental and behavioural disorders)	25-34	7.3	6	0.2983
State of Mexico	Valle de Chalco Solidaridad	Chapter 5 (Mental and behavioural disorders)	35-44	3.2	10	0.9751
State of Mexico	Valle de Chalco Solidaridad	Chapter 5 (Mental and behavioural disorders)	45-54	10.1	10	0.4298
State of Mexico	Valle de Chalco Solidaridad	Chapter 5 (Mental and behavioural disorders)	65+	14.6	10	0.1490
State of Mexico	Valle de Chalco Solidaridad	Chapter 5 (Mental and behavioural disorders)	55-64	5.7	10	0.8398
State of Mexico	Tonanitla	Chapter 9 (Diseases of the circulatory system)	55-64	3.8	5	0.5761
State of Mexico	Tonanitla	Chapter 9 (Diseases of the circulatory system)	65+	7.6	10	0.6672

State of Mexico	Tonanitla	Chapter 9 (Diseases of the circulatory system)	45-54	1.6	6	0.9510
State of Mexico	Tonanitla	Chapter 4 (Endocrine, nutritional and metabolic diseases)	55-64	8.6	10	0.5712
State of Mexico	Tonanitla	Chapter 4 (Endocrine, nutritional and metabolic diseases)	65+	8.4	10	0.5875
State of Mexico	Tonanitla	Chapter 4 (Endocrine, nutritional and metabolic diseases)	45-54	3.0	6	0.8131
State of Mexico	Tonanitla	Chapter 10 (Diseases of the respiratory system)	65+	8.0	10	0.6244
State of Mexico	Tonanitla	Chapter 10 (Diseases of the respiratory system)	55-64	2.7	6	0.8505
